# Recent advances in critical nodes of embryo engineering technology

**DOI:** 10.7150/thno.58799

**Published:** 2021-05-25

**Authors:** Youwen Ma, Mingwei Gu, Liguo Chen, Hao Shen, Yifan Pan, Yan Pang, Sheng Miao, Ruiqing Tong, Haibo Huang, Yichen Zhu, Lining Sun

**Affiliations:** 1 School of Mechanical and Electric Engineering, Jiangsu Provincial Key Laboratory of Advanced Robotics, Soochow University, Suzhou 215123, China.; 2 Cardiology, Dushuhu Public Hospital Affiliated to Soochow University, Suzhou 215000, China.; 3 Jiangsu Key Laboratory of Neuropsychiatric Diseases and Cambridge-Suda Genomic Resource Center, Soochow University, Suzhou 215123, China.; 4 State Key Laboratory of Robotics & Systems, Harbin Institute of Technology, Harbin, China.

**Keywords:** oocytes, embryo engineering technology, micromanipulation, electrical activation, electrofusion, *in vitro* culture

## Abstract

The normal development and maturation of oocytes and sperm, the formation of fertilized ova, the implantation of early embryos, and the growth and development of foetuses are the biological basis of mammalian reproduction. Therefore, research on oocytes has always occupied a very important position in the life sciences and reproductive medicine fields. Various embryo engineering technologies for oocytes, early embryo formation and subsequent developmental stages and different target sites, such as gene editing, intracytoplasmic sperm injection (ICSI), preimplantation genetic diagnosis (PGD), and somatic cell nuclear transfer (SCNT) technologies, have all been established and widely used in industrialization. However, as research continues to deepen and target species become more advanced, embryo engineering technology has also been developing in a more complex and sophisticated direction. At the same time, the success rate also shows a declining trend, resulting in an extension of the research and development cycle and rising costs. By studying the existing embryo engineering technology process, we discovered three critical nodes that have the greatest impact on the development of oocytes and early embryos, namely, oocyte micromanipulation, oocyte electrical activation/reconstructed embryo electrofusion, and the *in vitro* culture of early embryos. This article mainly demonstrates the efforts made by researchers in the relevant technologies of these three critical nodes from an engineering perspective, analyses the shortcomings of the current technology, and proposes a plan and prospects for the development of embryo engineering technology in the future.

## 1. Introduction

Oocytes are formed by two meiotic divisions of oogonia. In the natural fertilization state, when the sperm passes through the uterus and fallopian tubes, the layer of disabling factor on the surface of the sperm secreted from the epididymis is removed; thus, the sperm has the capacity to fertilize an ovum. The sperm contacts the zona pellucida and binds to the sperm receptor (ZP3). Under the action of acrolein and N-amidase, the sperm nucleus and cytoplasm enter the cytoplasm of the oocyte through the zona pellucida. At this time, a large number of cortical granules in the outer cytoplasm below the oocyte membrane release their content into the perivitelline space, causing changes in the ZP3 glycoprotein molecules in the zona pellucida and causing the zona pellucida to lose its function of accepting sperm penetration. The reaction prevents the occurrence of multiple inseminations and polyspermy and ensures the biological characteristics of human monospermy. Under the stimulation of sperm penetration, the oocyte recovers and quickly completes the second meiosis II, forming a mature oocyte, and the second polar body is discharged into the perivitelline space. Since then, the male and female pronuclei are formed separately, and chromosomes are replicated at the same time. The double pronuclei move closer to the middle and merge to form a diploid zygote, and the fertilization process ends. Therefore, the number, morphology and mobility of live sperm and normal oocyte development are important conditions for ensuring fertilization. When one of these conditions is not met, normal fertilization cannot be completed *in vitro*. To solve this problem, embryo engineering technology aimed at improving the efficiency of *in vitro* development (IVP) has been developed.

The rapid development of embryo engineering technology has facilitated the leapfrog development of life sciences and reproductive medicine. Gene editing technology 1, cloning technology 2,3, artificial assisted reproduction 4 and other technologies have been created and widely used in industrialization. At present, various embryo engineering technologies for oocytes, early embryo formation and subsequent developmental stages and different target sites have been developed, such as *in vitro* fertilization (IVF) 5, intracytoplasmic sperm injection (ICSI) 6, preimplantation genetic diagnosis (PGD) 7, and somatic cell nuclear transfer (SCNT) 8. With the continuous deepening of research, the development of embryo engineering technology has shown two major trends. First, the target species of operation tend to be more advanced. For example, in the field of cloning technology, after more than 70 years of development, the operating objects range from amphibians 9 to lower mammals 10 to non-human primates 11, and the species level has been increasing. The acquisition and manipulation of oocytes is becoming increasingly difficult. Second, with continuous in-depth research on the formation of fertilized ova and early embryo development in the *in vitro* environment, various new technical requirements for micromanipulation have been proposed. The new technologies themselves have become increasingly sophisticated and complex, and the difficulty of implementation has also been increasing. The declining success rate has become the biggest problem facing the development of embryo engineering technology. Similarly, in the case of cloned animals, the differentiated somatic cell nucleus is transferred into the enucleated oocyte to reprogram the chromatin of the somatic cell, and then the embryo develops into a complete individual. As an important developmental biology research method, it has great potential for use in many aspects, such as agricultural breeding, biomedicine, and cherished animal protection. Somatic cells can achieve pluripotency through SCNT. However, due to various epigenetic barriers (DNA methylation, histone modification, miRNA regulation, etc.) in reprogramming, which makes somatic cell reprogramming incomplete, there is an extremely low potential for nuclear transfer embryo development. For example, the success rate of somatic cell cloning technology ranges from 5% for cattle 12 to 2% for pigs 13 and monkeys 11, and its success rate is less than 1%. This greatly limits the application prospects of this technology. The low success rate brings two major dilemmas. The first is that the research and development (R&D) cycle is getting increasingly longer, and the second is that the R&D cost is getting increasingly higher. More importantly, from the perspective of industrial development, technologies with a success rate that is too low have no industrial application value. Therefore, the low success rate has become the largest bottleneck hindering the further development of life sciences and reproductive medicine based on embryo engineering technology. Any technology that helps to effectively improve the success rate will provide tremendous help to related industries and bring very considerable economic and social benefits. This article mainly introduces how the application of various new technologies can improve the success rate of embryo engineering technology from the perspective of engineering.

**Figure 1:**
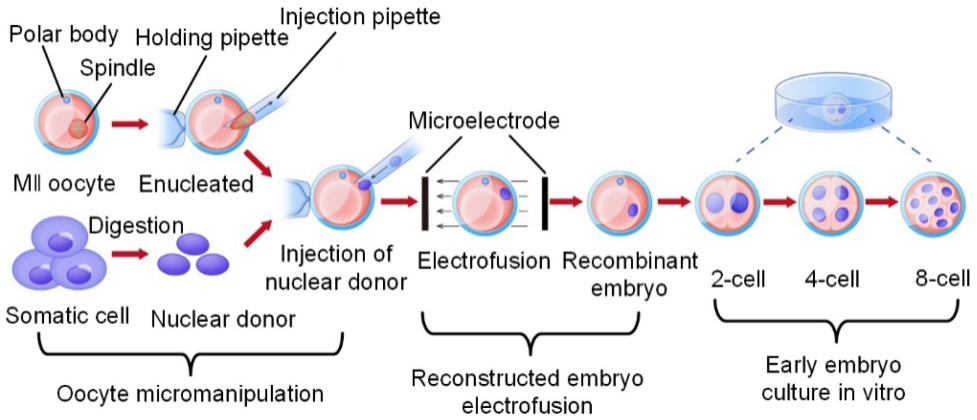
Critical nodes in the process of embryo engineering technology (take SCNT as an example).

As shown in **Figure 1**, we discovered three high-risk nodes in the embryo engineering technology process that have the greatest impact on the development of oocytes and embryos, namely, oocyte micromanipulation, oocyte electrical activation/reconstructed embryo electrofusion, and early embryo *in vitro* culture. In the whole process of embryo engineering technology, the survival rate of oocytes passing through these three critical nodes to develop smoothly to the next stage is low. Therefore, how to help oocytes pass through these high-risk nodes has become a key issue that needs to be resolved. At present, scientists have carried out much research on related aspects.

This paper focuses on the development of embryo engineering technology. The second to fourth sections of this paper describe the work that scientists have done to date in terms of the three high-risk nodes mentioned above and explain how the application of new techniques can improve the efficiency of embryo engineering technology and the survival rate through these high-risk nodes. In conclusion, the fifth section summarizes the current research status and challenges of these three critical nodes and proposes the prospect of future development directions. The fifth section analyses the research status and existing bottlenecks of these three critical nodes and proposes corresponding solutions and prospects for future development directions.

## 2. Oocyte micromanipulation

First, in the process of embryo engineering technology, micromanipulation is the first hurdle that oocytes will confront. At present, the main micromanipulations on oocytes include: The sperm tail is trimmed and the sperm head is injected into the oocyte during ICSI; The nucleus of the oocyte is extracted and a new nucleus is injected during SCNT; One cell of the 4~8-cell stage embryo or the first/second polar body of the oocyte before or after fertilization is taken to detect whether the gene is defective during PGD. These micromanipulations on oocytes involve the modification and reorganization of the internal structure and material of the oocyte, such as performing a complex “surgery” on the oocyte, which is the most damaging step to the oocyte. It is also the most critical step in determining the survival rate. The accuracy of the manipulation part, the control of the extraction/injection quantity, and how to minimize the damage to the oocytes in the process of breaking through the zona pellucida are three critical problems that we must face. We summarize these issues as follows: targeted micromanipulation, minimally invasive micromanipulation and quantitative micromanipulation. From these three perspectives, researchers have carried out substantial work.

### 2.1 Targeted micromanipulation

Targeted micromanipulation is the precise manipulation of specific parts of the oocyte. To achieve this goal, it is first necessary to identify the internal structure of the oocyte through microscopic image identification technology to find the best operating position and operating angle, adjust the oocyte to the best position suitable for manipulation and fix it to facilitate the next manipulation.

#### 2.1.1 Image identification in micromanipulation

The identification of the oocyte and the end-effector is the first problem that needs to be solved to realize targeted microinjection. The traditional method is to magnify the target area through a microscope, and then the observer visually finds and identifies the oocytes and end-effectors 14. This method requires high-level practitioners and is inefficient. At present, the automatic identification of oocytes and end-effectors by computer vision has become the mainstream 15. Generally, images are collected by a charge-coupled device (CCD) camera. After greyscale conversion, noise filtering and signal enhancement, the images are transmitted to the computer memory through an image acquisition card, which is then called and processed by an image processing algorithm, and finally, the identification of the target object (end-effector and oocyte) is completed.

**Figure 2:**
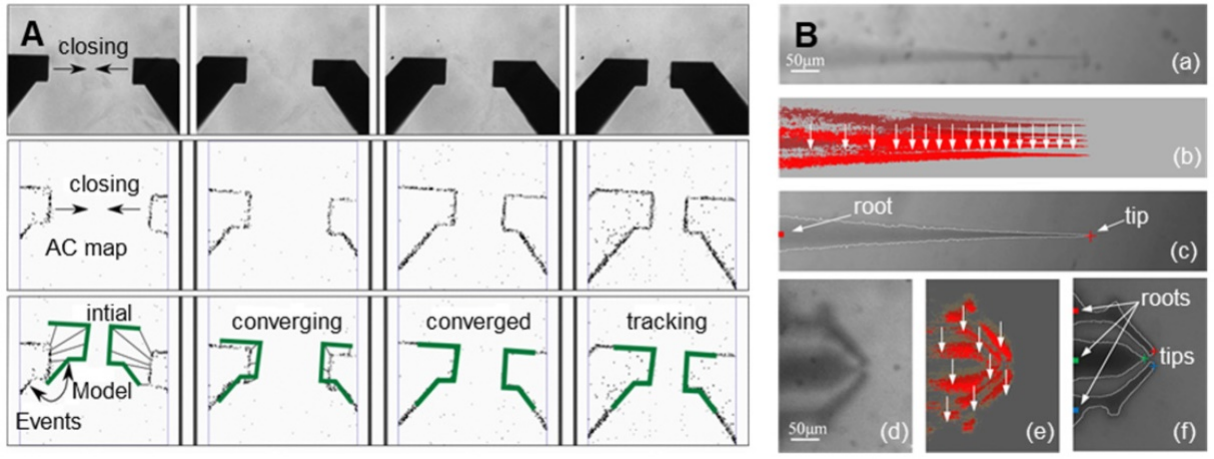
** Image recognition of the end-effector.** (A) Track the position of the microgripper through an iterative closest point algorithm. Adapted with permission from 18. (B) Recognize microinjection needles and microtweezers through algorithms based on motion history images (MHI) and active contour models. Adapted with permission from 18, copyright 2012 IEEE, and 22, copyright 2013 IEEE.

The image identification of the end-effector realizes the precise control of the end-effector by the computer and the mechanical arm 16,17. Due to the characteristics of the end-effector and movement characteristics of oocyte micromanipulation, high-speed and high-precision identification and tracking of targets is the goal of the identification algorithm. Ni et al. 18 proposed an iterative nearest point algorithm to track the position of the microgripper. **Figure 2A** shows the principle of EICP tracking. The first row shows the gradual closing process of the fixture, the middle row is the cumulative graph of events, and the last row shows the edge tracking of the gripper by the EICP model. However, the implementation of this algorithm requires the use of additional dynamic vision sensors on the hardware, which is relatively complex in terms of system integration. Anis et al. 19,20 used template matching methods to track micromanipulators with obvious features. However, in many cases, micromanipulators do not have obvious features, and template matching has real-time problems in detection. Sun et al. 21 used a sum-of-squared-differences optical flow (SSD) algorithm to track the injection needle in oocyte injection, but SSD was effective only when there were no significant changes between successive frames of images. Liu et al. 22 developed an algorithm based on the motion history images (MHIs) and an active contour model to locate the tip of the end-effector. **Figure 2B** shows the recognition of the tips of micropipettes and microgrippers using this algorithm. Figures (a) - (c) depict the original image of the micropipette, corresponding MHI of the moving micropipette, and tip detection result based on active contour, respectively. Figures (d) - (f) show the original image of the microgripper, corresponding MHI of the moving microgripper, and tip detection result based on active contour. The algorithm can accurately locate the end-effector in the defocusing state or not in the image field, but the threshold in MHI detection must be selected appropriately to balance the algorithm failure rate and manipulation efficiency, and the selection of the threshold is sensitive to light. In addition, algorithms such as support vector machines 23 and Kalman filters 24 have also been used to detect or track objects in microscopic imaging systems. These algorithms have certain pertinences and great limitations.

The image recognition of oocytes distinguishes the different organelles of oocytes to perform precise manipulations. The structure of oocytes is complex and includes dominant structures such as the zona pellucida, cell membrane, cytoplasm, and polar bodies, and recessive structures, such as the cytoskeleton, spindle, and mitochondria. Dominant structures can be observed directly under an optical microscope, while recessive structures can only be observed with the help of special techniques.

**Figure 3:**
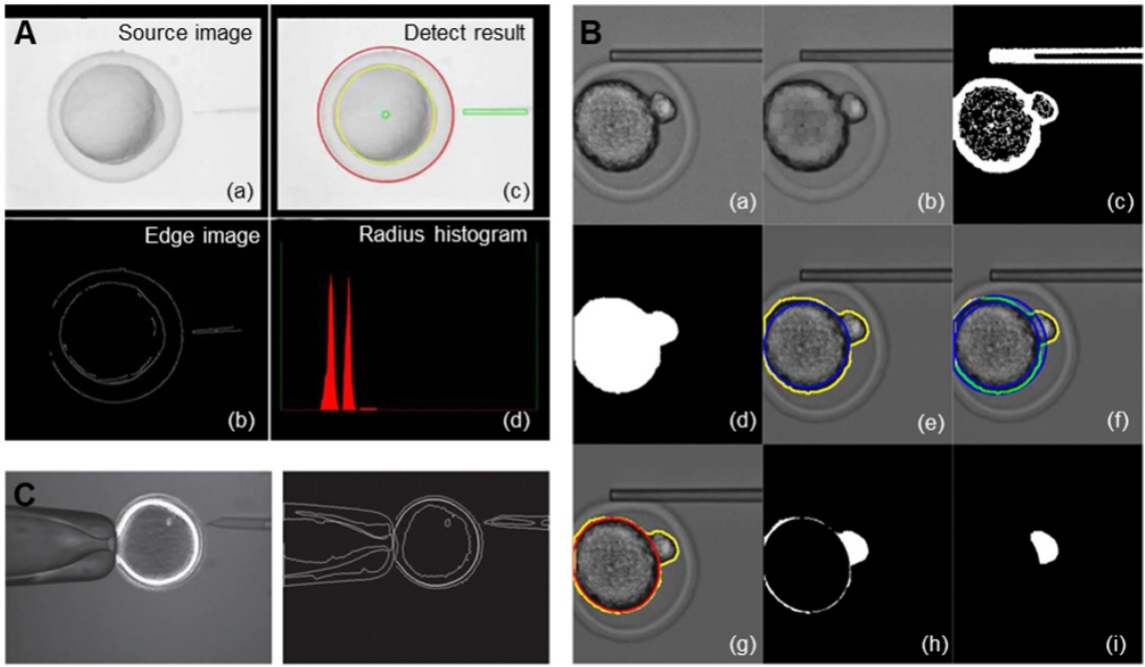
** Image recognition of different parts of oocytes.** (A) Detection of the chorion and cytoplasm of zebrafish oocytes and the edge of the microinjection needle by the Canny algorithm. (B) Detection of the existence and direction of polar bodies through image processing. (C) The fusion images from polarized light microscopy imaging systems and the traditional optical inverted microscope imaging system and the edge information of the fusion image. Adapted with permission from 34, copyright 2009 IEEE, and 39, copyright 2017 IEEE, and 48, copyright 2013 IEEE.

The recognition of oocyte contours mainly uses image segmentation technology and contour extraction technology. Image segmentation is usually performed before contour extraction to make the contour of the image more prominent 25, 26. The basis of image segmentation is based on the grey level difference between the object and the background. The minimum value between the two peaks in the grey distribution histogram is selected as the best threshold to threshold the graph 27. Since most suspended cells have a spherical or nearly spherical shape, after image segmentation is completed, the contour of the cell image can be extracted by the geometric figure extraction algorithm. Common edge detection algorithms include the Sobel algorithm 28, 29, Canny algorithm 30, Hough transform 31, 32, snake model 33, etc. Huang et al. 34 applied the Canny edge detection algorithm to automatically detect the contour of the chorion and cytoplasm of zebrafish oocytes and the edge of a microinjection needle in their experiment and adopted chord midpoint Hough transformation (CMHT) to improve the efficiency and accuracy of ellipse detection in the images. **Figure 3A** shows how the vision system captures and processes images. Among them, figure (a) is the original image of the zebrafish embryo, figure (b) and (c) are edge detection results of the original image, and the final figure (d) is the histogram of the zebrafish embryo radius.

The image identification of the oocyte polar body is mainly used to rotate the oocyte to a proper posture to avoid the polar body during microinjection. Leung et al. 35 identified the polar bodies of mouse oocytes/embryos, first using morphological manipulations to obtain the general contours of the cytoplasm and the polar body, then fitting the contours of the cytoplasm, and finally using the differences between the two contours to obtain the polar body region. Sun et al. 36 proposed a new method to automatically locate the polar bodies. This method uses contour tracking to search for oocytes and then identifies polar bodies through the support vector machine (SVM) algorithm. Later, these authors proposed a polar body detection method based on machine learning 37, which used an improved histogram of gradient (HOG) algorithm to extract polar body image features to increase the success rate. At the same time, a position prediction method was proposed to reduce the search range of the polar body and improve the efficiency. In their latest study, these researchers proposed a framework based on deep learning to realize polar body detection in oocyte rotation 38. The typical convolutional neural network for medical image segmentation is improved for polar body detection, especially for defocusing polar bodies and polar bodies of different sizes. In addition, these investigators designed a special image transformation method to simulate more oocyte rotation conditions, including oocyte and pole deformation, so that the deformed pole in oocyte rotation can be detected by this method. Wang et al. 39 realized automatic focusing on the pipette and oocytes and simultaneously introduced image processing technology, which could detect the presence and direction of the polar body when the oocyte was rotating at the tip of the pipette. As shown in **Figure 3B**, figure (a) is the original cropped image, figure (b) is the apply greyscale image opening, figure (c) is the Otsu's thresholding of (b) and Sobel edge detection of (a), figure (d) is the morphological filter and hole filling, figure (e) is the contour of the intracellular structure and its inscribed circle in yellow and blue lines, respectively, figure (f) is the overlapping part of the ring of the inscribed circle and the intracellular structure contour indicated in green, figure (g) is the ellipse fitting based on the overlapping part (the red ellipse is used to estimate the portion of cytoplasm), figure (h) is the estimated cytoplasm from (d) and figure (i) is the largest suitable blob is taken as the polar body after the binary image open operation. Mozafar et al. 40 developed a visual identification system for identifying oocytes and their polar bodies. The gradient-weighted Hough transform method is used to detect the position of the oocyte and its polar body to notify the automatic injection mechanism to avoid the polar body. In addition, considering the morphological differences between oocytes, they adopted a new elliptic fitting method to measure the size of oocytes and their polar bodies. The proposed algorithm is designed to be suitable for typical commercial inverted microscopes with different standards.

The purpose of image identification for oocyte spindles is to extract the nuclei of oocytes precisely and improve the success rate and efficiency of experiments. The traditional method is mainly to observe the spindle through the fluorescent probe method 41-43 or the polarized light imaging method 44-47. The fluorescent probe method stains oocytes with dye and then observes the chromosomes of oocytes under a fluorescence microscope. However, fluorescent dyes have toxic side effects on oocytes, and excitation light close to ultraviolet light can damage the oocytes. Polarized light microscopy imaging systems can image the spindle of oocytes without injury, but they will lose much operating environment information, such as the precise location of the end-effector and oocyte texture. Huang et al. proposed a multicue image fusion method 48,49, which fused the images from polarization optical microscopy and traditional inverted optical microscopy, retaining their respective advantages. The fusion result is shown in **Figure 3C**; the fusion result is on the left, and the edge information of the fusion result is on the right. The fused image can clearly observe the spindle in the oocyte without losing the information of the operating environment.

#### 2.1.2 The manipulation of the oocyte

After accurately identifying the internal structure of oocytes, the operating position and operating angle were determined. Since an ordinary optical microscope can only provide two-dimensional image information and the operating space is limited, the traditional way to move the end-effector to the right position is not suitable. However, it is a better choice to adjust the oocyte to the most suitable posture and then fix it through various techniques; this approach is called oocyte manipulation. In the process of oocyte micromanipulation, the techniques for adjusting oocyte posture can be classified into contact methods and noncontact methods. The contact methods mainly fix the oocytes through negative pressure holding or microtraps and then adjust the oocytes to a proper posture by operating tools. Noncontact methods involve changing the position and posture of oocytes through noncontact techniques such as microfluidics, dielectrophoresis, optical tweezers or magnetic fields and then fixing them. **Table 1** summarizes the current common contact and noncontact oocyte operating and controlling methods.

**Figure 4:**
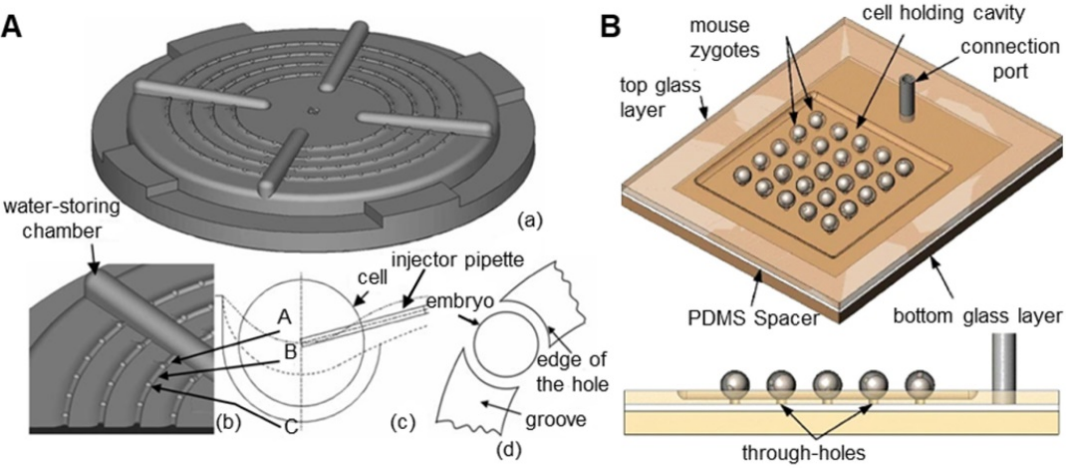
** Contact cell fixation device.** (A) The zebrafish embryo fixation device with arrayed hemispherical grooves. (B) The glass chip with arrayed round holes. Adapted with permission from 34, copyright 2009 IEEE, and 53, copyright 2009 Springer Nature.

The traditional method is to attach a microcapillary to an air pump, which generates negative pressure to suction oocytes 50,51. However, this method can only manipulate one oocyte at a time and requires high precision and stability of the air pump. The oocyte will be damaged if the suction force is too large. Many researchers have prepared devices that can fix oocytes in batches. Lu et al. 52 immobilized zebrafish embryos through rows of parallel V-shaped grooves made of gel at the bottom of a Petri dish in microinjection experiments, but this method has strict requirements on the direction of injection. As shown in **Figure 4A**, Huang et al. 34 used agarose gel to make a zebrafish embryo fixation device with an array of hemispherical grooves. Compared with the previous device, this device allows injection from any direction and is biocompatible. However, the size of the hemispherical groove will have a large effect on the experiment. As shown in **Figure 4B**, Sun et al. 53 designed a glass device with array round holes for batch fixation of oocytes. This device combines negative pressure suction and a microtrap and has a large air chamber, which can improve the stability of negative pressure suction and improve the survival rate of oocytes.

**Figure 5:**
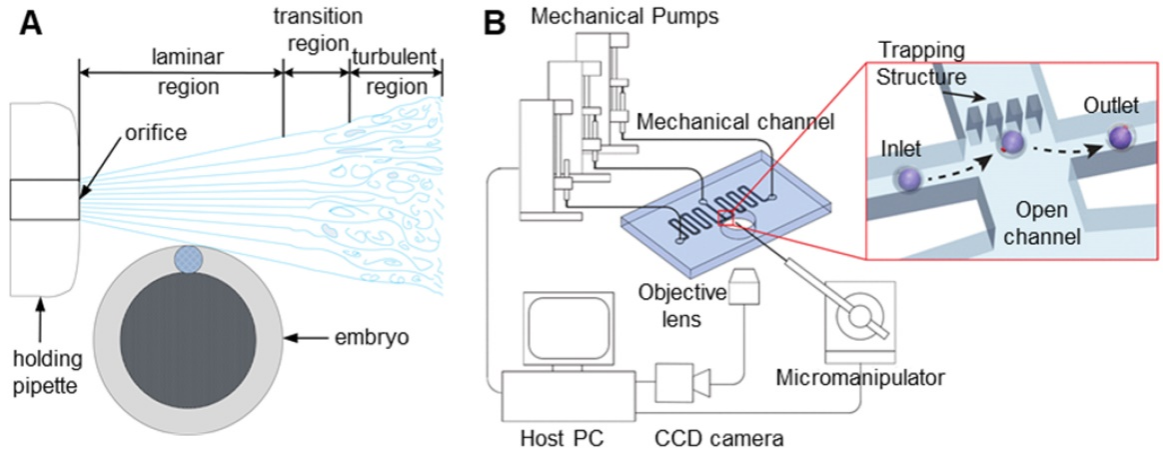
(A) Sun uses the torque generated by the fluid flow to rotate the cells. (B) A microfluidic platform used to achieve oocyte capture and direction control. Adapted with permission from 35, copyright 2012 IEEE, and 58, copyright 2013 IEEE.

In noncontact oocyte manipulation technologies, microfluidics mainly uses microfluidic flow to generate forces acting on oocytes, thereby fixing oocytes or adjusting their poses 54-57. This method does not require contact with oocytes and causes little damage to oocytes, but its process is complicated and inefficient. As shown in **Figure 5A**, when Sun et al. 35 analysed fluid flow, they found that fluid flow can generate a torque that makes the oocyte rotate. The fixed microcapillary can produce laminar and turbulent flow. The turbulence is chaotic, lacks stability and cannot be used. Laminar flow has smooth characteristics and is ideal for driving cells to rotate. Laminar and turbulent regions can be controlled by adjusting the flow rate of the fluid produced by the microcapillary. A lower flow rate leads to a larger laminar flow area. Therefore, oocyte rotation can be achieved by controlling the flow velocity of the fluid in the microcapillary. Shin et al. 58 designed a microfluidic platform to automate the process of oocyte capture and direction control through fluid dynamics and vision-based position control. The microfluidic channel and system configuration are shown in **Figure 5B**. A direction control algorithm based on the movement of oocytes in the microchannel is proposed. Visual tracking of the polar body is used to provide information about the embryo direction. The experimental results show that the embryo direction can be automatically controlled without manual intervention. Conventional microinjectors can access the device through the lumen so that fixed oocytes can be manipulated.

**Figure 6:**
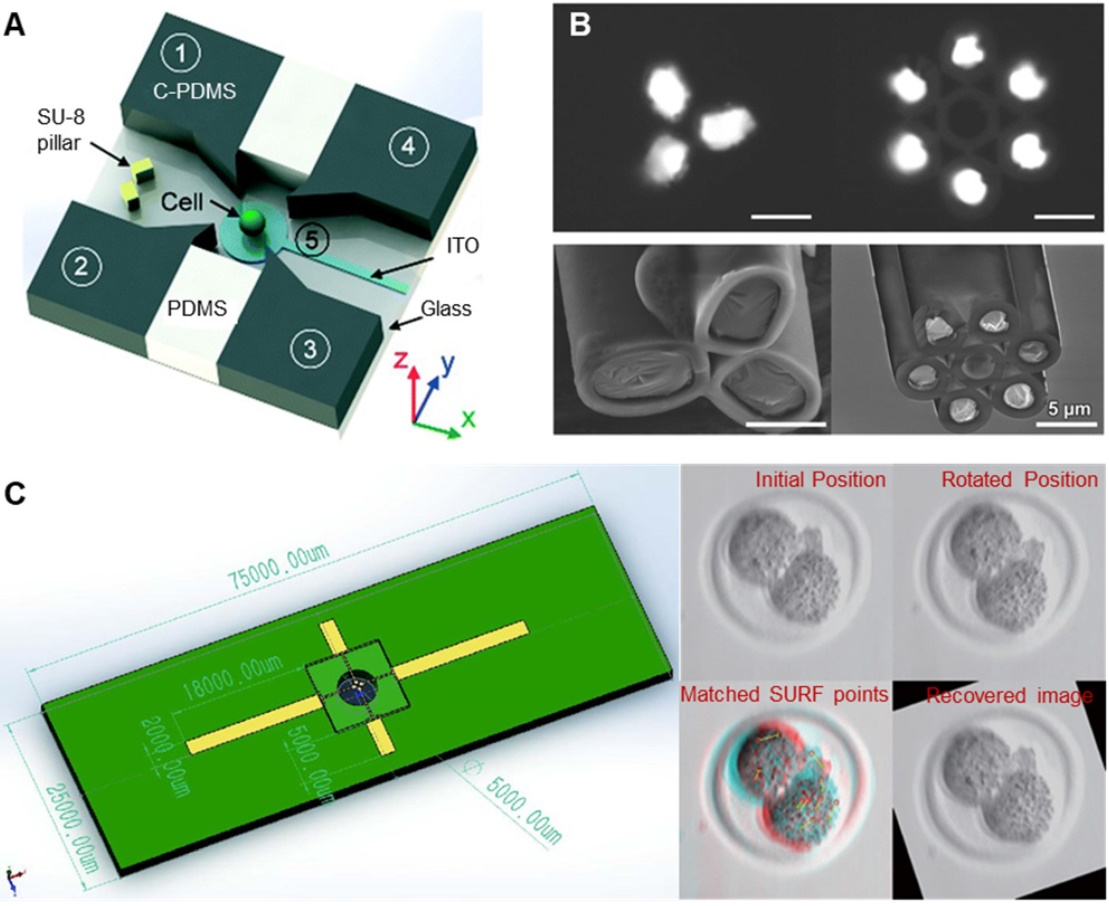
(A) Schematic diagram of a dielectrophoretic chip. (B) Scanning electron microscopy image of liquid metal-based capillary microneedle electrodes. (C) The effect of dielectrophoresis forceps and drive oocyte rotation. Adapted with permission from 62, copyright 2018 Royal Society of Chemistry, and 63, copyright 2018 John Wiley and Sons, and 64, copyright 2020 IEEE.

Dielectrophoresis (DEP) refers to the process in which oocytes are polarized in nonuniform electric fields and subjected to dielectrophoretic forces. Thus, oocytes can be controlled by adjusting electric field parameters to change the dielectrophoretic forces 62-64. As shown in **Figure 6A**, Huang et al. 62 designed a dielectrophoretic chip consisting of four vertical carbon-black-nanoparticle-PDMS (C-PDMS) electrodes, an ITO electrode at the bottom, and a single cell trap/release module. The trap/release module allows a single cell to be hydrodynamically captured and then transferred to a rotating cavity formed by electrodes. The cell can be rotated in 3D by applying appropriate AC signals to the electrodes. As shown in **Figure 6B**, Yu et al. 63 prepared a capillary microneedle electrode based on liquid metal by injecting liquid metal gallium into a multitube glass capillary. By applying multifrequency, multiamplitude, multiphase AC electrical signals to the microelectrodes, three-dimensional dielectric electrophoresis traps and one-dimensional electrical rotation can be generated simultaneously, realizing 4D single-cell manipulation. As shown in **Figure 6C**, Huang et al. 64 connected an inverted microchip with four electrodes to a manipulator as robotic dielectric electrophoresis tweezers to drive the embryo lying on the petri dish for rotation. A vision-based algorithm was developed to obtain the position and direction of the embryo and provide feedback signals to achieve precision control of the embryo. Compared with the first two systems, this system can quickly process large numbers of cells in a contactless, low-cost, and flexible manner. Manipulating oocytes based on DEP can have a high accuracy and a fast response speed, but whether polarization in a nonuniform electric field will cause damage to oocytes has not been studied.

Optical tweezers provide a new method for cellular manipulation 65-67. Optical tweezers capture and move objects using the force exerted by a strongly focused beam of light. The intensity gradient in the converging beam draws small particles to the focus, and particles can be captured in three dimensions near the focus. We can move and rotate cells by changing the focus of the laser beam. Zhou et al. 68 summarized the working principles and unique functions of various optical tweezers as well as their applications in biology. However, the manipulation force provided by optical tweezers is relatively small, which is slightly insufficient in manipulating oocytes. Cells and biological tissues have a certain ability to absorb light, and using optical tweezers to manipulate cells may cause potential heat damage. The magnetic field method attaches magnetic particles on the cell surface and then drives the cell to move or rotate by driving the magnetic particles on them 69-73. These two methods have been successfully applied to the manipulation of nanoparticles and somatic cells but have not yet been successfully applied to oocyte manipulation. However, these two methods have the potential to manipulate oocytes and may be further developed by researchers.

### 2.2 Minimally invasive micromanipulation

The thick zona pellucida outside the oocyte becomes the barrier we need to break through after determining the precise operating position of the oocyte and adjusting the posture of the oocyte to meet the end-effector. Manual manipulation mainly reduces the damage to oocytes through thinner microcapillary and higher puncture speed. However, if the needle is too thin, it will be too soft and will affect the suction or injection of substance. Therefore, the diameter of the needle tip cannot be reduced indefinitely. To reduce the damage to oocytes and evaluate the damage to oocytes in the process of membrane puncturing, many new technologies have been applied. Researchers first believe that the force received by oocytes during membrane puncture is an important factor for oocyte injury and hope to measure and control the micropuncture force. At the same time, some advanced membrane puncture technologies have also been used to improve oocyte micromanipulation devices.

#### 2.2.1 Measurement of micropuncture force

In the oocyte membrane puncturing experiment, the micropuncture force exerted on the oocyte is usually on the order of micronewtons. As shown in **Table 2**, the micropuncture force measurement methods are mainly divided into direct and indirect measurements 74,75. The direct measurement method is mainly used to measure the film puncturing force through microforce sensors. There are many kinds of force sensors to measure the film puncturing force, such as piezoresistive force sensors, capacitive force sensors, piezoelectric force sensors and optical force sensors. Indirect measurement methods mainly include vision-based methods, calculation methods and actuator input methods.

**Figure 7:**
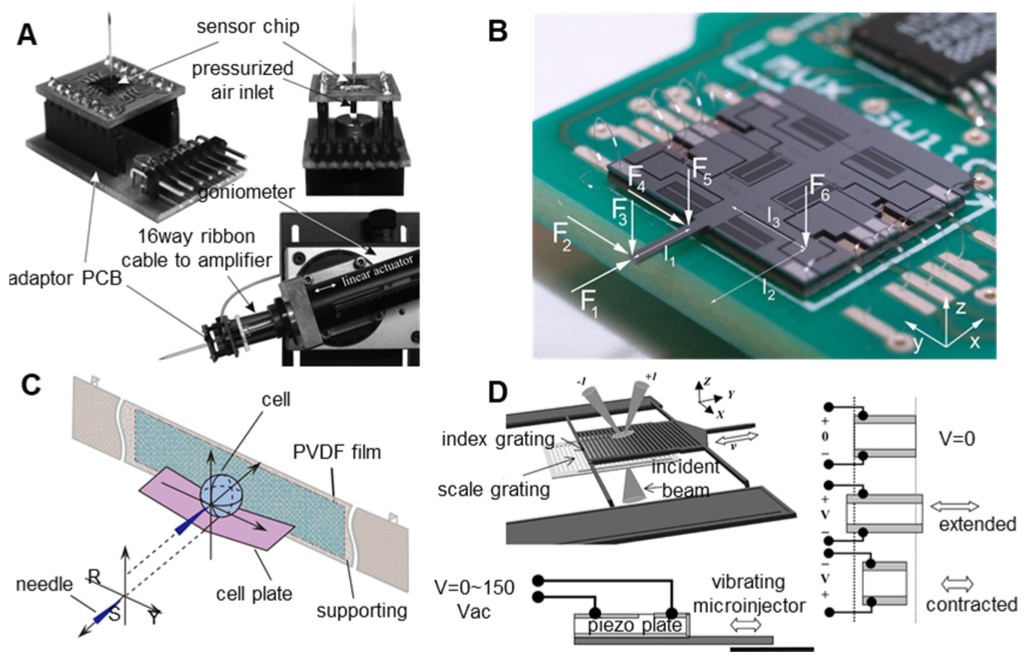
** Various sensors for measuring the injection force.** (A) Piezoresistive force sensor. (B) Capacitive six-axis force sensor based on the MEMS process. (C) Microforce sensor based on piezoelectric material PVDF. (D) A micro-optical force sensor. Adapted with permission from 52, copyright 2012 IEEE, and 81, copyright 2009 IEEE, and 84, copyright 2009 SAGE Publications, and 87, copyright 2006 IEEE.

The resistance of the piezoresistive force sensor will change with the variation in the external force, and the size of the external force can be obtained by measuring the resistance 76,77. Due to the simple structure, the resistive force sensor can be easily combined with the existing microinjection system, but it requires high assembly accuracy and is easily affected by environmental temperature changes. As shown in **Figure 7A**, Beutel et al. 77 integrated a silicon piezoresistive microforce sensor on the tail of a microcapillary to measure the injection force during microinjection. Thanks to the rapid development of microelectromechanical systems (MEMS) technology, a variety of capacitive force sensors based on MEMS technology 78-81 have been prepared. This sensor has advantages including low power, low noise, large range, and high sensitivity, can measure the force information of multiple axes and is not sensitive to environmental temperature changes. However, capacitive force sensors also have some limitations including complex structures, short lives, and a difficult integration with existing microinjection systems. As shown in **Figure 7B**, Beyeler et al. 81 designed a capacitive force sensor that can measure the force or moment of six axes and has a resolution of micronewtons and nanonewtons. The piezoelectric effect sensor is mainly prepared based on the piezoelectric effect of piezoelectric materials (piezoelectric ceramics (PZT) and polyvinylidene fluoride (PVDF)). 82-84. Piezoelectric sensors have the advantages of a large force measurement range, a high bandwidth, a small size and a high power density, but they are also vulnerable to temperature changes and signal drift caused by charge leakage. Therefore, piezoelectric sensors are more suitable for dynamic force measurements but not for static force measurements. **Figure 7C** shows the injection force sensing scheme designed by Xie et al. 84. The microforce sensor adopts a simple supported beam structure. A PVDF film is adhesively bonded to the back of the supporting beam. The cell plate is well placed on the beam such that the centre points of the beam, the PVDF film, and the cell coincide with each other. When the needle penetrates into the cell along the extension line of the RO, the process can be considered a quasi-static process. According to Newton's law, the cell penetration force equals the force applied on the PVDF film. Optical force sensors measure force based on changes in the intensity or phase of light signals 85-87. The optical force sensor can measure the injection force without contact. The optical force sensor has the advantages of high sensitivity, anti-electromagnetic interference, good reproducibility, no hysteresis, and no limitation of the operating space. However, optical force sensors are easily affected by light reflection or refraction. Since cells absorb light energy, optical force sensors can facilitate potential thermal damage to cells. Using near-infrared light to form optical force sensors can reduce damage to cells. As shown in **Figure 7D**, Zhang et al. 87 integrated an optical MEMS force sensor on a vibrating microinjector, which consists of two vertically separated microgratings. In the experiment, a HeNe laser (633 nm/4 mW) was used to illuminate the grating. The injection force is determined by the relative displacement of the two gratings, which is determined by the intensity distribution of the diffraction orders.

Among the indirect measurement methods, the vision-based method mainly uses microscopic image processing and an accurate oocyte mechanics model to determine the micropuncture force 88-90. Force is usually calculated based on the deformation of visually tracked flexible objects (such as oocytes and end-effectors). The measured geometric information is processed by the force estimation algorithm to obtain the micropuncture force. This method is suitable for dynamic measurements, but due to the limitation of the oocyte mechanics model, the precision of the measurement results is not very high. The calculation method solves the unknown force through various force functions 91,92. This method is suitable for detecting forces that are not easy to measure directly, but the structure of the instrument is complex. Others indirectly estimated the injection force through the input of the actuator 93,94. This method is suitable for online measurement and control, but the precision is poor. In addition, there are many other indirect measurement methods. Because of the poor accuracy of indirect measurement methods, it will not be introduced in too much detail here.

#### 2.2.2 Control of the micropuncture force

**Figure 8:**
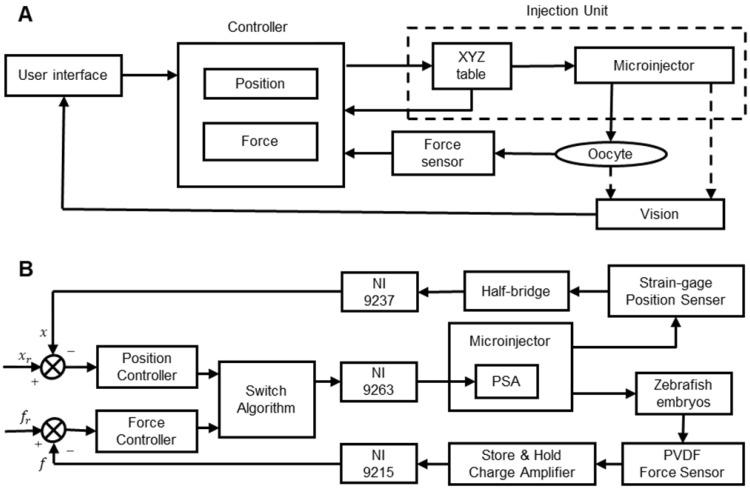
(A) Schematic diagram of the cell injection system with inner loop impedance control and outer loop feedback control. (B) An injection system solution with position/force switch control. Adapted with permission from 84, copyright 2009 SAGE Publications, and 97, copyright 2017 IEEE.

The control of the micropuncture force is very important in oocyte membrane puncturing experiments. If the micropuncture force is too small, then it will not be able to penetrate the zona pellucida and cell membrane of the oocyte. If the injection force is too large, then the oocyte may be hurt or even die. Therefore, achieving the precise control of the injection force has become the key to improving the success rate. Xie et al. 84 developed a cell injection method based on force control, which can adjust the penetration force according to the desired force trajectory. As shown in **Figure 8A**, the proposed force control framework includes two control loops. The inner loop is an impedance control used to specify the interaction between the microcapillary and the embryo. The outer loop is a force tracking nonlinear controller using feedback linearization technology. With the proposed force control method, the puncture force can be clearly adjusted during the embryo injection process to follow the desired force trajectory. Karimirad et al. 95 proposed a vision-based method to model a precision load cell with artificial neural networks. The proposed neural network model combined with the boundary detection algorithm can act as a load cell capable of measuring force with a range of ***µN*** to ***mN***. The algorithm can trace and characterize embryo deformations by extracting geometric features called “pit angles” directly from images of the embryo micromanipulation process. The neural network was trained with the experimental data of zebrafish embryo micromanipulation, and the trained neural network was suitable for indentation of any other spherical elastic object. The results of this study can be used to measure forces during microinjection of biological cells, such as mouse oocytes/embryos, and are particularly suitable for situations requiring force feedback. Sun et al. 96 developed a static PVDF microforce sensor based on the inverse model method to detect the cell injection force and developed a fuzzy-PID feedback-based closed-loop control method to control the cell injection force. Experiments show that the system can track and control the injection force in cell micromanipulation, but the accuracy and stability need to be further improved. Wang et al. 97 designed a piezo-driven cell injection system that combines force and position control. The PVDF force sensor for detecting the micropuncture force and the strain gauge sensor for measuring the microcapillary relative position in real time are integrated into the system. As shown in **Figure 8B**, by introducing the weight coefficient method, the challenge of transient pulsation between force and position controller switching can be reduced. An adaptive sliding mode control method with parameter estimation is used to compensate for the hysteresis nonlinearity and disturbance of the piezoelectric actuator. The injection force is controlled by an incremental PID controller. The whole system is low cost, easy to install and easy to maintain.

#### 2.2.3 Improvement of micropuncture technology

The conventional microinjector penetrates the zona pellucida and cell membrane of oocytes by mechanical squeezing. During the puncture process, a certain force and initial velocity must be given to the microcapillary tip 98, 99. However, as the zona pellucida of oocytes is extremely tough, mechanical squeezing will cause large deformation of oocytes, which can severely damage or even kill oocytes. Subsequently, piezo-driven ultrasonic microinjectors based on the inverse piezoelectric effect were developed 100, but the piezoelectric actuator produced the axial vibration required for puncture and produced harmful lateral vibration. Excessive lateral vibrations will tear and kill the oocytes. Some researchers have added mercury or heavy oil to the tip of the microcapillary to attenuate the lateral vibration 101-102. Although this method is effective, mercury and heavy oil will have toxic side effects on fragile oocytes. Other researchers used micromotors to rotate microcapillaries to reduce damage to oocytes 103, but the results were modest.

**Figure 9:**
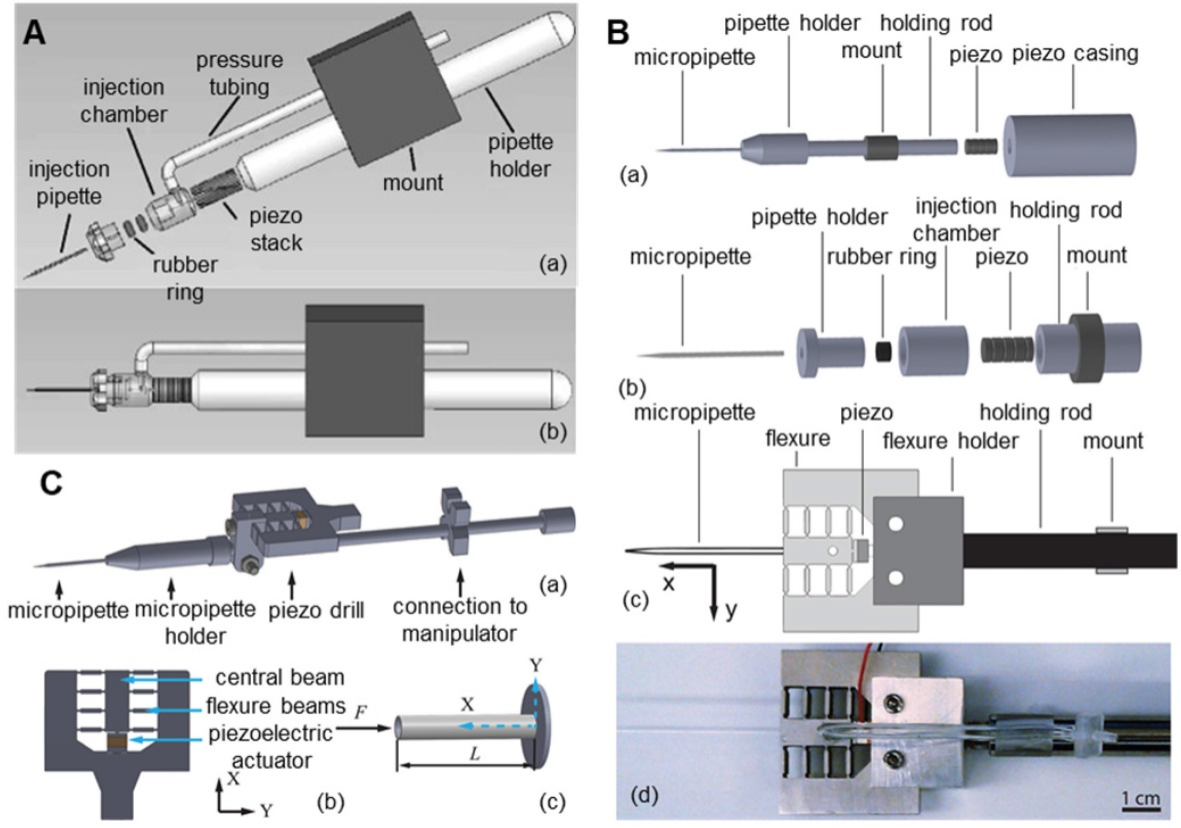
** Various improved piezoelectric ultrasonic microinjectors.** (A) Microinjector with piezoelectric ceramic front. (B) Piezoelectric ultrasonic microinjector with a flexible mechanism. (C) Microinjector with piezoelectric actuator eccentric configuration. Adapted with permission from 104, copyright 2011 Spring Nature, and 106, copyright 2018 IEEE, and 107, copyright 2020 IEEE.

To reduce the lateral vibration of the piezoelectric ultrasonic microinjector, researchers began to improve its structure. As shown in **Figure 9A**, Huang et al. 104,105 proposed a novel design of a piezo-driven ultrasonic microinjector. By prepositioning the piezoceramics, the power of piezoelectric oscillations is concentrated on the pipette of the syringe, thus eliminating the vibration effect on other parts of the micromanipulator and reducing harmful lateral vibration. In the automatic cell injection experiment on zebrafish embryos (n=200), the microcapillary penetrated oocytes at low speed and with small deformation. The success rate was 96%, and the survival rate was 80.7%. As shown in **Figure 9B**, Johnson et al. 106 connected the piezoelectric ceramic and the microcapillary with a flexure mechanism on the basis of the piezoelectric ceramic to further reduce the lateral vibration. In the puncture experiment of mouse oocytes (n=45), the puncture success rate reached 100% and only caused a 3.4 µm deformation of the oocyte during the puncture process. As shown in **Figure 9C**, Dai et al. 107 installed the piezoelectric actuator next to the holder of the microcapillary by adopting the eccentric configuration of the piezoelectric actuator, which has the advantage of not affecting the installation of the standard microcapillary. At the same time, these authors also developed a technology to correlate filters and motion history images based on angular feature probability data to automatically detect the cell membrane rupture through piezoelectric drilling. This technology can automatically stop the piezoelectric vibration immediately after the microcapillary penetrates the oocyte membrane. Compared with manual stopping, the time delay between membrane puncture and the stopping of piezoelectric vibration was greatly reduced, thus improving the survival rate after oocyte puncture. In addition, many researchers have improved piezoelectric ultrasonic microinjectors and achieved good results.

At present, a great deal of work has been carried out in the study of oocyte injury. However, in terms of reducing oocyte injury during microinjection, these previous studies still have deficiencies. First, research on oocyte injury has basically focused on the cell surface, such as the zona pellucida. However, there are a large number of skeletal structures inside the cell, and the injury of these structures cannot be ignored. At present, relevant studies have established a more detailed mechanical model of oocytes to study and evaluate the mechanism of injury to cell internal fine and cytoskeletal structure during microinjection. For example, Liu et al. 108 established a kinetic model based on dissipated particles to study the degree of oocyte damage during microinjection. The damage to oocytes was simulated by studying the number of broken molecular bonds in oocytes. The greater the number of broken chemical bonds is, the greater the damage degree of oocytes. By studying the damage mechanism of oocytes during microinjection, the optimal parameters of oocyte membrane breaking can be set, which will be the key research direction of minimally invasive oocyte micromanipulation in the future.

### 2.3 Quantitative micromanipulation

In micromanipulation, it is often required to implement material exchange inside or outside the oocyte through injection or extraction, which is mainly achieved by microcapillary connections with air or liquid pumps. When the microcapillary is inserted into the oocyte, the positive/negative pressure of the air pump is adjusted to push/suck material into/outside the oocytes through the microcapillary 109,110. In the SCNT experiment, oocytes underwent extraction and injection of nuclei. Controlling the amount of extraction and injection is the key to the success of the experiment. In the ICSI experiment, a single sperm is injected into oocytes, and the control of the injection volume is not only related to the success of the experiment but also involves ethical issues. The microinjection quantity is at the picolitre level. To achieve high-precision quantitative microinjection, researchers have mainly conducted investigations from the following two directions: develop high-precision syringe pumps to actively improve the accuracy of suction and injection or detect and control the quantity in real time during the suction and injection process.

#### 2.3.1 High-precision injection pump

**Figure 10:**
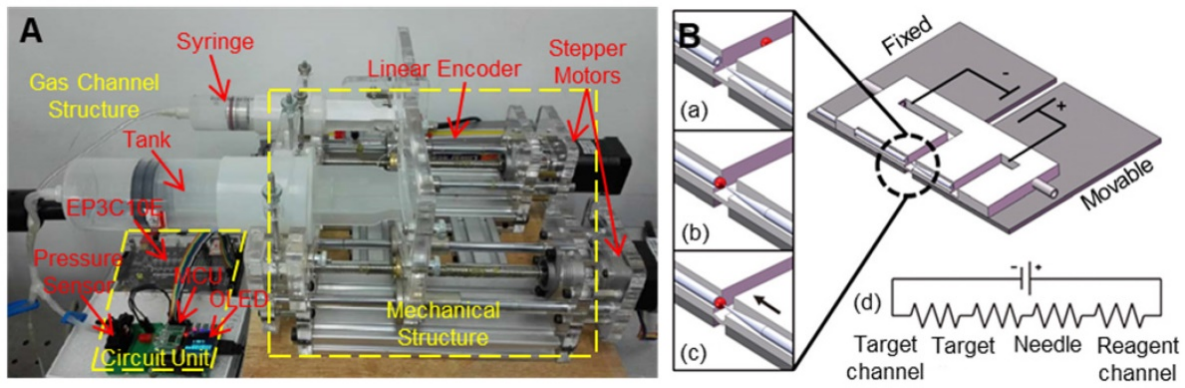
(A) High-precision pressure-driven pump for the quantitative injection of picolitre. (B) Electrical schematic diagram of the injection system based on electroosmosis. Adapted with permission from 111, copyright 2017 World Scientific Publishing Co, and 112, copyright 2009 Royal Society of Chemistry.

Microinjection experiments are usually carried out on commercial injection pumps using an air or oil pump as a pressure source. Commercial injection pumps include CellTram 4r Air and CellTram 4r Oil (Eppendorf), IM-400 and IM-400B (Narishige). However, due to the instability and accuracy of the pressure source, commercial pumps have some problems with respect to picolitre level injection, and the success rate varies greatly between different operators. Li et al. 111 developed a high-precision pressure-driven pump for quantitative injection of a subpicolitre. As shown in **Figure 10A**, by connecting the buffer tank to the gas channel, the new pump system has higher stability and resolution, various positive and negative pressures between -30 kPa and +150 kPa can be generated, the pressure control accuracy is less than 1 Pa, and the precision of the injection volume can reach 0.0022 pl. Noori et al. 112 realized the pumping of reagents through electroosmosis in an experiment. Electroosmosis is induced by applying a potential to the electrode embedded in the target and the reagent supply channel, as shown in **Figure 10B**. The advantage of using electroosmosis for reagent transport is that it eliminates the need for dedicated pumps and does not limit the size of the microcapillary, providing true scalability for the microcapillary. In addition, electroosmosis provides nonpulsed flow and precise electrical quantity control.

#### 2.3.2 Detection and control of injection quantity in real time

Zhao et al. 113 proposed a new microcapillary aspiration method based on a common pneumatic microinjection system. This method is the first to use the equilibrium pressure model to quantify the effect of the capillary effect on suction or injection pressure. The method has the ability to quantify suction pressure and detect the joint between oocytes and microcapillary tissue. Wang et al. 114 first considered the influence of intracellular pressure on the microinjection amount. The experiments showed that intracellular pressure may result in an error of up to 30% between the actual injection quantity and the set value. In this work, the relationship between intracellular pressure and injection quantity was analysed and modelled to compensate for the control of the injection quantity. The experiments showed that the difference between the expected set value and the actual injection quantity was less than 3% after the intracellular pressure was compensated. Liu 115 obtained the critical injection pressure of the microcapillary through theoretical analysis and experimental verification, which provided reference data for quantitative cell injection. Chow et al. 116 controlled the injection quantity in the experiment by controlling the injection pressure and the opening time of the pressure and found a linear relationship between the injection amount and the injection pressure and a linear relationship between the injection amount and the injection time. This method does not require additional special equipment, but when the injection substance, injection target and microcapillary diameter change, the relationship among the injection pressure, pressure opening time and injection quantity needs to be recalculated again, which is very complicated. Sun et al. 117 proposed a method to characterize the injection volume of the robotic cell injection system by measuring the fluorescence intensity of the injected cells. The injection volume was determined by the changes in the fluorescence intensity of the cells before and after the injection. The injection volume can be controlled by adjusting the injection parameters (such as injection time and pressure) or the concentration of the substance to be injected by feeding back to the experiment. Huang et al. 118 designed a self-sensing microflow injection device based on piezoelectric ceramics. Based on the characteristics of the microdisplacement produced by the inverse piezoelectric effect, the injection volume was controlled by adjusting the size, frequency and time of the driving voltage. The self-induced displacement in the microinjection process can be obtained by using the normal piezoelectric effect, which realizes the integration of the sensor and actuator. The experiment showed that the device has good reliability and repeatability.

### 2.4 Conclusion for oocyte micromanipulation

The oocyte micromanipulation node is the most complicated node in the embryo engineering technology process and the node that causes the greatest damage to the oocyte. The quality of its completion directly affects the subsequent nodes. Therefore, much research has been focused on this node. At present, researchers have been trying to replace traditional manual operations with machine operations, and machine-assisted oocyte micromanipulation has been developing towards a targeted, minimally invasive, and quantitative direction. The application of the automatic identification and positioning technology of oocytes and end-effectors has greatly improved the degree of the automation of oocyte microinjection. The application of various contact and noncontact control methods has realized the control of oocytes with precise posture adjustment and fixation. The emergence of piezoelectric ultrasonic microscopic rupture technology makes it possible to complete the rupture of oocytes with minor damage to the oocytes. The application of various microinjection force sensors and control technologies makes it possible that the injection force is known and controllable. The development of high-precision syringe pumps and the closed-loop control technology of injection dose allow the exchange of materials inside and outside the oocytes to achieve nanoliter-level precision. These technologies use the machine's high sensitivity, high precision, and high stability to compensate for the lack of manual operation stability to improve the efficiency and success rate of micromanipulation. Although these technologies have shown certain advantages in terms of the degree of automation, in terms of the core index success rate, the results of machined micromanipulations have not shown much advantage over manual operations. The addition of various complex advanced technologies also increases the complexity of the system and increases the difficulty of operation for actual operators. This is also the inherent reason why machine-assisted micromanipulation technology still cannot replace manual operation and cannot be widely promoted and applied. Therefore, how to demonstrate the technical advantages of machine-assisted micromanipulation technology in the core index success rate will be a problem that we need to seriously think about and solve.

## 3. Oocyte electrical activation/reconstructed embryo electrofusion

In the process of embryo engineering technology, oocyte electrical activation/reconstructed embryo electrofusion is the second critical node that oocytes will confront. Various bioelectrical signals play an important role in the natural development of oocytes. Bioelectric stimulation can change the oocyte zona pellucida and the movement of ions in the solution to achieve various effects. Electrical stimulation is also a common method in various stages of oocyte micromanipulation. For example, during natural fertilization, oocyte activation is accomplished by sperm entry and induced calcium oscillation. In the process of parthenogenetic activation (PA) and ICSI, the artificially assisted activation of oocytes is essential. Presently known artificially assisted activation methods include mechanical stimulation 119, 120, chemical activation (enzymes 121, calcium ionophores 122, 123, ethanol 124, 125, etc.), and electrical activation 126, 127. The unique advantages of electrical stimulation lie in that the parameters are easier to accurately control, the manipulation is simple, and there is no chemical toxicity. In addition, during SCNT, the fusion of oocytes and somatic cells is also accomplished by electrofusion.

### 3.1 Principles of electrical activation/electrofusion

Electrical stimulation techniques related to oocytes are mainly divided into electrical activation and electrofusion. Electrical activation means that the oocyte membrane is stimulated by a high-voltage DC pulse, and calcium ions flow into the oocyte as a second messenger through ion channels on the cell membrane, causing changes in the chemical substances in the oocyte, that is, stimulating the endoplasmic reticulum in the oocyte. The stored calcium pool is released, thereby increasing the concentration of Ca^2+^. On the one hand, the increased Ca^2+^ content destroys the existing cytostatic factor (CSF), thereby reducing the activity of the maturation promoting factor (MPF). On the other hand, increased Ca^2+^ causes the degradation of cyclin sensitive to Ca^2+^, initiates the activation of oocytes, and prompts oocytes to leave the MⅡ phase to complete embryonic development after meiosis. Electrofusion refers to the use of electrical pulses to create recoverable micropores in the recipient cell and donor cell in the fusion solution so that the two cell membranes fuse with each other. If the electric pulse is too large, then the micropores in the cell membrane will not be repaired. Thus, an appropriate electric pulse can make the cell membranes fuse, and the cytoplasm of the cells can facilitate communication and finally merge into one cell. Both electrical activation and electrofusion are the principles for establishing electroporation. In essence, the phospholipid bilayer structure of the cell membrane changes under the stimulation of electrical pulses. Electroporation refers to the generation of micropores in the cell membrane under the action of a high-intensity electric field, which improves the permeability of the cell membrane 128,129. The micropores produced in the cell membrane allow substances in the culture medium to enter the cells, and many manipulations of oocytes are based on this theory 130.

When calculating the membrane potential difference, the cell membrane is regarded as an insulator, and the cytoplasm inside the cell and the suspension outside the cell are both regarded as electrolyte solutions. Under the action of an external electric field, both the cytoplasm and the cell suspension are polarized, and a membrane is produced. The potential difference ***V_m_*** is given by formula (3-1):


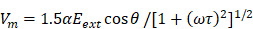
(3-1)

where ***E_ext_*** is the applied electric field intensity, ***θ*** is the angle between the radial direction at an arbitrary point on the membrane and the electric field direction, and ***τ*** is the relaxation time constant of the dielectric membrane. When the frequency of the applied alternating electric field is low enough, we assume ***ωτ* << 1**, or when the applied electric field is a direct current (DC) electric field, formula (3-1) can be simplified to formula (3-2):


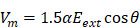
(3-2)

With two cellular extremum points, ***θ* = 0° *or* 180°**, the membrane potential difference induced by the applied electric field is further simplified as Eq. (3-3):


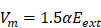
(3-3)

The potential difference is the largest at these two points. The induced membrane potential increases with increasing applied electric field intensity. In cell electroporation, the cell membrane will be perforated when the intensity reaches a special value: ***V = V_cr_***. Under this condition, the induced membrane potential ***V_cr_*** is called the critical membrane potential, while the applied electric field ***E_cr_*** is called the critical applied electric field. When the intensity of the applied electric field is higher than ***E_cr_***, the cell membrane begins to perforate. The higher the applied electric field intensity is, more or larger holes occur on the membrane. However, if the applied electric field is too strong, cell perforation becomes irreversible, and cells can be permanently damaged.

The above is the theoretical basis of cell electroporation, but the actual situation is much more complicated than this. Virtually all cells are not ideal spheres, different cells vary in size and shape, and the composition of their membranes and media varies. Therefore, when performing cell electroporation experiments, the theoretically calculated values are only for reference, and the best parameters are obtained through continuous experiments. Since cells have a strong ability to repair themselves, they can withstand large pulse amplitudes. Scientists have found that even low amplitude pulses can cause electroporation, but stronger amplitude pulses can achieve higher electroporation efficiency. The lower limit of the pulse width must be longer than the charging time of the membrane and the elastic recovery time of the membrane to keep the pores open. There is a complementary relationship between the pulse width and amplitude. To reduce the pulse amplitude, the pulse width needs to be widened to compensate; the efficiency of perforation is often proportional to the product of the pulse amplitude and the width. The most commonly used pulse waveforms are DC square wave pulses and exponentially decayed resistance-capacitance (RC) pulses. If the electrical pulse can maintain the membrane pores open for a period of time with a lower voltage after breaking through the cell membrane, then it will help improve the efficiency of cell electroporation. Therefore, RC pulses are often more effective than DC pulses of the same amplitude. In addition, increasing the number of pulses can obviously increase the efficiency of cell electroporation, but the death rate of cells also increases.

### 3.2 Application of electric activation/electrofusion

Researchers have used electrical stimulation to activate oocytes when studying PA and have carried out experimental exploration on many organisms, such as pigs 131-134, cows 135, and mice 136. The specific research contents and conclusions are summarized in **Table 3**. By analysing the experimental data of previous researchers, we found that when parthenogenetic activation of oocytes is carried out, the effect of electrical activation (EA) is obviously better than that of chemical activation (CA) and mechanical activation (MA), and the combination of EA and CA can obtain a better effect.

Electrical stimulation is also widely used in ICSI to stimulate oocytes. Experimental studies have been conducted on many animals, such as rabbits 137, 138, pigs 139, and humans 140-142. The specific research content and conclusions are summarized in **Table 4**. By analysing the experimental data of previous scientific researchers, we found that electrical activation can significantly increase the fertilization rate and cleavage rate of oocytes after ICSI, and the electrical activation of oocytes can be applied before or after ICSI.

In SCNT, the fusion of somatic cells and enucleated oocytes usually uses electrofusion. Experimental studies have been conducted on many animals, such as pigs 143-154, cattle 146-150, goats 151, 152, and mice 153-155. The specific research content and conclusions are summarized in **Table 5**. By analysing the experimental conclusions in **Table 5**, we found that appropriate chemical treatment of oocytes after electrofusion can improve the success rate of fusion. Generally, the electric field intensity required for electrical activation of oocytes should be lower than the electric field intensity required for electrofusion. However, when analysing the experimental data in **Table 4** and **Table 5**, we found that this conclusion was not obvious, which may be due to the various oocytes used in the experiment, the composition of the culture medium, and the electric activation/electrofusion device being different.

### 3.3 Electrical activation/electrofusion device

Traditional cell electrofusion systems use a fusion chamber with two parallel plate electrodes for electrofusion. The distance between two parallel plate electrodes is 1~10 mm, and the fusion electric field is generally ~kV/cm, which makes the voltage applied to the parallel plate electrodes reach hundreds to thousands of volts. Due to the surface tension of the liquid and the electrochemical reaction between the electrode and the buffer solution to produce bubbles, the electric field distribution in the electrofusion chamber is not uniform. In addition, the posture of the cells between the parallel plate electrodes is difficult to unify, causing fluctuations in the effect of electrical stimulation/electrofusion. Due to the limitations of traditional electrofusion systems, the effect of electrical stimulation/electrofusion on cells is very rough. Even the same device, the same batch of oocytes, and the same electrical parameters have different effects. Therefore, the miniaturization and refinement of oocyte electrical stimulation/electrofusion devices is an inevitable trend.

**Figure 11:**
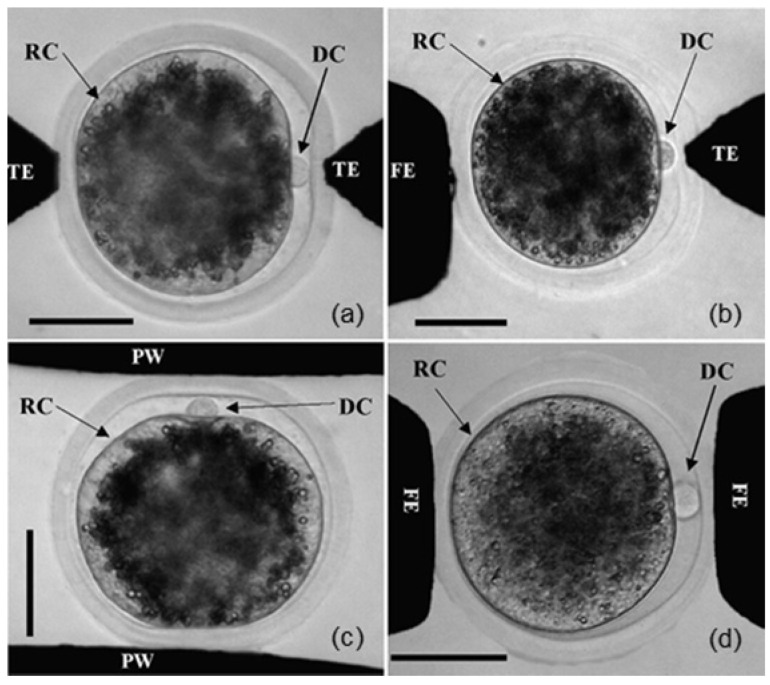
** Schematic diagram of four electrofusion schemes.** (a) Tip-end plus tip-end (TT); (b) tip-end plus frustum-end (TF); (c) frustum-end plus frustum-end (FF); (d) parallel microelectrodes (PM). Adapted with permission from 156, copyright 2007 Elsevier.

Liu et al. 156 analysed the effects of the electrofusion of goat SCNT embryos using electrodes of different shapes. As shown in **Figure 11**, the experiment used three kinds of microelectrodes: 15 μm tip-end, 100 μm frustum-end and 200 μm parallel microelectrodes. These microelectrodes were combined into four groups for the experiment: tip-end plus tip-end (TT), tip-end plus frustum-end (TF), frustum-end plus frustum-end (FF) and parallel microelectrodes (PM). In the four schemes, the interface between the nuclear donor and oocyte was perpendicular to the electric field. When the electrodes did not apply pressure to the reconstruction configuration, the fusion rate of the four groups was not significantly different; all were approximately 74%. When pressure was applied, the TT group showed a higher fusion rate (94.9%) and a lower degradation rate (2.1%). Compared with traditional electrode chamber fusion, electrofusion with pressure applied to the tip needle microelectrode is a better SCNT electrofusion scheme.

**Figure 12:**
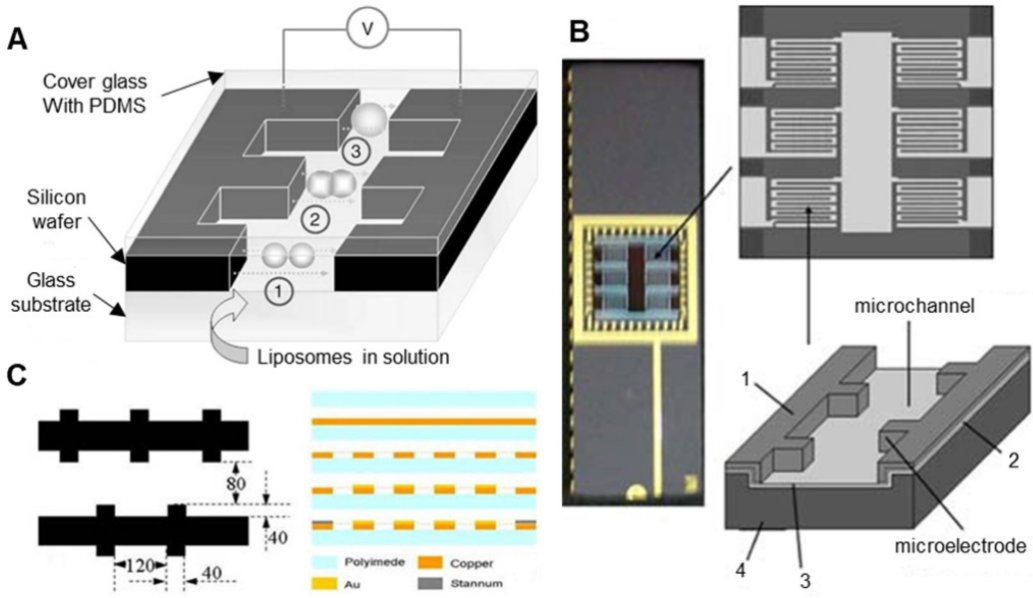
** Various electrofusion chips with interdigitated electrodes.** (A) A schematic diagram of a microfluidic chip with a high-aspect-ratio microelectrode array. (B) An electrofusion chip with 1368 pairs of silicon microelectrodes. (C) A schematic diagram of the flexible electrofusion chip. unit: ***µm***. Adapted with permission from 157, copyright 2004 Spring Nature, and 158, copyright 2008 Springer Nature, and 159, copyright 2009 Elsevier.

At the same time, under the premise of ensuring the success rate of oocyte electrical activation/electrofusion, microfluidic chips have inherent advantages in improving the efficiency of electrical activation/electrofusion. Although no related research on oocyte electrical activation/fusion chips has been reported, there are indeed many studies on microfluidic electrofusion chips for somatic cells, which can be referenced for future development of high-precision oocyte electrical stimulation chips. Researchers first found that cell pairing can be achieved by using dielectrophoresis force. A kind of cell electrofusion microfluidic chip with interdigitated microelectrodes was developed 157-159. This microfluidic chip uses an alternating current to achieve cell pairing during manipulation and then direct current pulses to complete cell fusion. As shown in **Figure 12A**, Guillaume et al. 157 fabricated a microfluidic electrofusion device with a high-aspect-ratio microelectrode array. A 250 μm thick silicon electrode was bonded to a glass substrate by photolithography. The electrode was covered with a glass substrate coated with PDMS. The electrode spacing varied from 30 μm to 500 μm. The liposome cells were sequestered and paired with an alternating current of 0.1-0.2 kV/cm and 300 kHz. Then, 5 to 6 DC pulses of 5-10 kV/cm were applied for electrofusion. Cao et al. 158 produced a microfluidic chip with a similar structure for the electrofusion of plant protoplasts. As shown in **Figure 12B**, the microfluidic chip has 6 microchambers and a total of 1368 pairs of high-aspect-ratio silicon microelectrodes. The microchannels were 20 μm deep and 80 μm wide, and the electrode spacing was 50-100 μm, with increments of 10 μm. In the experiment, a 1.2 kV/cm, 2 MHz alternating current was used to pair the plant protoplasts; several 4 kV/cm, 20-50 μs direct current pulses were used for electrofusion. Hu et al. 159 used flexible printed circuit board (FPCB) technology to produce a new type of flexible cell electrofusion chip. As shown in **Figure 12C**, 2.2×10^4^ microelectrodes were integrated on the chip, which can perform cell electrofusion in large quantities. Human embryonic kidney cells (HEK-293) were paired under a 6 V, 1 MHz alternating current, and electrofusion was performed under 3 to 5 direct current pulses of 35 V for 50 μs.

**Figure 13:**
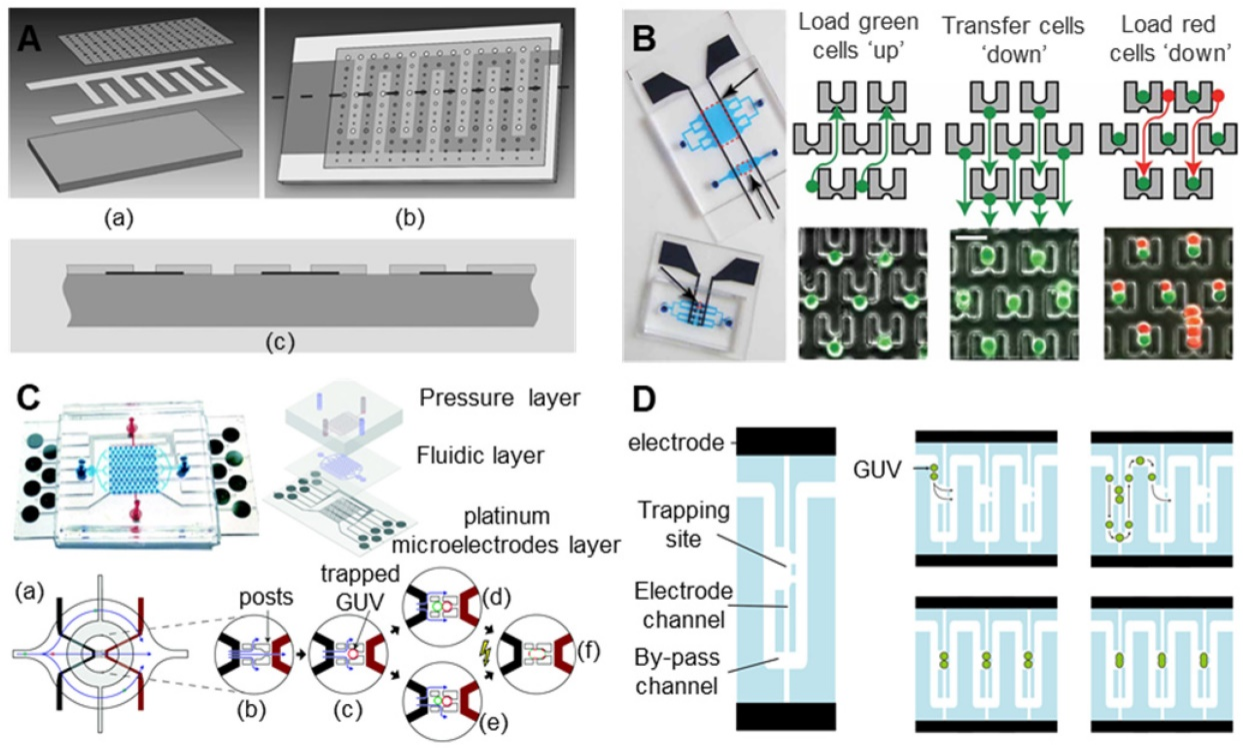
(A) A double-layer microfluidic chip integrating automatic cell positioning and cell electrofusion. (B) A microfluidic chip integrates thousands of microtraps made of PDMS and its working steps. (C) A microfluidic chip can separate cells of different diameters and perform electrofusion and its working schematic diagram. (D) A microfluidic chip for electrofusion of GUVs and its working diagram. Adapted with permission from 160, copyright 2010 Spring Nature, and 161, copyright 2009 Spring Nature, and 162, copyright 2014 Royal Society of Chemistry, and 163, copyright 2020 Elsevier.

Compared with traditional electrofusion systems, microfluidic chips containing interdigitated microelectrode arrays have the advantages of a low voltage, a simple structure, and a high throughput. However, when using the dielectrophoretic force generated by alternating current to pair the cells, the number of paired cells cannot be controlled, and the cells are easily trapped in the nonelectrofusion zone between the two adjacent electrodes, which greatly reduces the pairing efficiency of the cells. Therefore, it is a better choice to use microstructures in microfluidic chips for cell pairing. Clow et al. 160 designed a double-layer microfluidic chip that integrates automatic cell positioning and cell electrofusion. As shown in **Figure 13A**, interdigital titanium electrodes with a thickness of 300 μm, a width of 250 μm, and a pitch of 250 μm were prepared on a borosilicate glass substrate. The electrode was coated with an electrically insulating photosensitive epoxy polymer (SU-8) film with a thickness of 4 or 22 µm. Micropits with diameters of 10-80 µm were made above the electrode. During the experiment, it was necessary to manually load the donor somatic cells and the recipient oocytes, draw the cells into the micropits through DEP to achieve pairing, and then apply high-voltage direct current pulses for electrofusion. The device cleverly introduces the micropit structure onto the microfluidic electrofusion chip, which effectively improves the success rate of cell pairing. However, DEP is still needed to fix cells, and the manual loading of cells undoubtedly increases the cumbersomeness of the experiment and reduces the efficiency. Skelley et al. 161 fabricated a microfluidic chip with thousands of microtraps made of PDMS arranged on a glass substrate. As shown in **Figure 13B**, the microtraps used to capture cells consist of a larger frontal capture ring (14 μm tall, 18 μm wide, 25-40 μm deep), a smaller dorsal capture ring (14 μm tall, 10 μm wide, 5 μm deep), and a support ring (7.5 μm wide, 35-50 μm long, 6-8 μm tall). In the experiment, a single cell A (green fluorescence) was first captured with a smaller backside capture cup. Once the array was saturated, cell A was transferred directly “down” to the relatively large capture cup. Finally, cell B (red fluorescence) was loaded. Cell B was captured by the larger capture ring in front and paired with cell A, with a success rate of up to 70%. This design makes clever use of hydrodynamics and specially designed microtraps to capture and pair cells. Robinson, T et al. 162 manufactured a microfluidic chip that could detach giant unilamellar vesicles (GUVs) of different diameters and perform electrofusion. As shown in **Figure 13C**, the chip contains a three-layer structure: a circular ring valve is integrated on the pressure control layer, a PDMS trap for capturing target cells is integrated in the fluid layer, and a patterned electrode is integrated on the glass base. GUVs with diameters much larger than 20 μm (the size of the trap passage) will not enter the trap. GUVs with diameters less than 8 μm will escape through a gap in the trap, allowing them to capture target-sized GUVs and pair them up. After all traps are captured and paired, the ring valve in each chamber can be hydraulically driven to isolate each trap to prevent GUVs from being affected by fluid shear. Finally, the electrofusion of GUVs can be completed by the output of a DC pulse through the microelectrode. Sugahara, K et al. 163 also produced a microfluidic chip that can be used for electrofusion GUVs. As shown in **Figure 13D**, the chip structure is very simple and was prepared from polydimethylsiloxane (PDMS) using standard soft lithography. One side of the snake-like pathway in the centre of the chip is designed to trap cells, and the chip is flanked by electrodes. The GUV suspension was injected into the chip with a syringe pump at a flow rate of 400 nL/min. GUVs were captured when they flow through the trap on the side of the channel. The trap can capture two GUVs and complete the pairing. The subsequent GUVs will follow the bypass channel and continue to flow until caught by the next trap. After the pairing is completed, the GUVs are electrically fused with DC pulse input through the electrodes. However, it should be noted that neither the microfluidic fusion chip with an interdigitated electrode nor the microfluidic fusion chip with a “microtrap” has been successfully applied to oocytes. In the future, a microfluidic electrofusion chip more suitable for oocytes may be developed.

### 3.4 Conclusion for oocyte electrical activation/reconstructed embryo electrofusion

The oocyte electrical activation/reconstructed embryo electrofusion node is the weakest part of the embryo engineering technology process. Both electrical stimulation strategies and devices need to be improved. To date, much research work has been performed by researchers with biological backgrounds, focusing on adjusting various “optimal” electrical stimulation parameters to obtain a high activation rate/fusion rate. However, in many studies, only irregular conclusions have been obtained regarding the optimization parameters under the existing crude electrical stimulation device. This is also the underlying reason why the “optimal electrical stimulation parameters” obtained by relevant studies shown in **Tables 4 to 6** of section 3.2 are totally different. Therefore, to truly determine the law of electrical activation/electrofusion parameters, the miniaturization and refinement of experimental devices is an inevitable trend. To date, substantial research and application in somatic cell electrofusion with precision electrical stimulation technology based on microfluidic chip technology has been carried out. This technology has shown good technical advantages and will definitely be the future development trend for oocyte electrical stimulation technology.

## 4. Early embryo *in vitro* culture

In the process of oocyte micromanipulation, the *in vitro* culture of early embryos is the third key node that determines the ultimate success rate. In the natural development process, the nutrition of the early embryo comes from the secretion of the mother's oviduct and uterine cavity. Its cellular activities (division, gene expression, and metabolism) are affected by factors produced by the environment, the embryo itself, and the cells of the reproductive tract. With the continuous development of biotechnology, we can bypass the fallopian tube and transfer embryos that have developed to the blastocyst stage to the uterus for further development through *in vitro* culture. The development process of embryo culture technology *in vitro* essentially involves constantly approaching the natural embryonic development process, simulating the natural environment of embryo development and meeting changing nutritional needs during embryo development. Chemical and physical factors have been proven to be a prerequisite for improving the culture environment of preimplantation embryos and are the basis for improving the rate and survival of blastocysts. At the same time, the microenvironment and macroenvironment in which the embryo is cultured *in vitro* also play a vital role in the development of the embryo. This part starts with analysing the reproductive mechanism and influencing factors of early embryos and deeply analyses the complex environment of early embryo development. Then, from the perspective of engineering technology, the research progress of *in vitro* embryo culture technology was systematically introduced.

### 4.1 Developmental mechanism and influencing factors of early embryos

#### 4.1.1 Developmental mechanism of early embryos

The reproductive mechanism is a complicated problem of the interaction between fluid and structure. The sperm and oocyte meet to form a fertilized ovum, the fertilized ovum further develops to the cleavage and blastocyst stages, and the process of blastocyst transport to the uterus is completed in the fallopian tube. The fallopian tube plays an important role, and the whole reproductive process undergoes dynamic changes under mechanical and biochemical conditions 164. Under an optical microscope, the fallopian tube mucosa is longitudinally folded, with a cubic or columnar epithelial layer. The epithelial cells in the epithelial layer are mainly divided into secretory cells and ciliated cells. The fallopian tube is not static but is dynamic, including muscle contraction and cilia beating 165. Due to the intermittent contraction of the fallopian tube and uterus, the early embryo passes through the fallopian tube to reach the uterus and then uses the contraction of the myometrium from the cervix to the fundus to reach the implantation site at the bottom of the uterus 166. The segmental muscle contraction of the fallopian tube wall not only drives the ovum to perform related exercises but also causes agitation of the material in the fallopian tube to ensure the mixing of gametes and embryos with the secretions of the fallopian tube 167. The dynamic activity of the oviduct may directly change the surrounding environment of the embryo and promote the exchange of gas and biomolecules. The fallopian tube may play an important role in the mechanical action of the embryo. The conventional static culture mechanism cannot simulate the mechanical stimulation of the embryo, and simulating the microscopic environment of the structure of the fallopian tube may promote the development of the embryo.

#### 4.1.2 Factors influencing early embryonic development

After understanding the reproductive mechanism of early embryos, researchers have attempted to create a culture environment close to living body development for early embryos when they are cultured *in vitro*. Chemical and physical factors must be considered when early embryos are cultured *in vitro*. **Table 6** summarizes the effects of common chemical and physical factors on embryos.

Early embryos are prone to developmental block when they are cultured in a culture medium alone. An important reason is the lack of *in vitro* growth factors. These growth factors composed of proteins usually bind to factor-specific receptors on the cell surface and affect tissue growth and differentiation 183. At present, early embryos try to form a co-culture system with various cells (oviduct epithelial cells, endometrial cells, follicles, fibroblasts, etc.), which can effectively improve the development rate. The reason is that the cell monolayer can remove toxins from the culture medium, secrete nutrients needed by the embryo, and interact with the embryo. During the co-culture process, these cells can improve the embryo morphology and have a positive impact on the embryo implantation rate and pregnancy rate 184-186. *In vitro*, the growth factors secreted by monolayer cells can promote the maturation of oocytes and the development of early embryos. Co-cultured cells can release a variety of different growth factors in the culture medium, which can improve the development of co-cultured embryos. In addition, the co-culture method is beneficial for the embryonic development of some mammals (cattle 187, horses 188, pigs 189, sheep 190, mice 191), which is not suitable for all embryos and species. The components and functions of growth factors secreted by different cells are also different. The poor growth factors secreted in the process of co-culture will have adverse effects on early embryonic development 183.

The embryo density is also considered to be one of the important factors affecting embryo development. Increasing the proportion of embryos in the culture medium can promote embryo development *in vitro* 198-200. Compared with individual cultures, the co-culture of embryos and embryos can promote the development of fertilized ova to blastocysts because the embryo produces autocrine and paracrine factors, which make up for unfavourable culture conditions by interaction 201. In addition, the increase in the embryo density can produce more growth factors around the embryo, and the effect is similar to adding growth factors to the medium.

The development process of early embryos is a very complicated process, and complicated influencing factors have made it very difficult to completely simulate its internal environment *in vitro*. To date, in many biological and medical laboratories, conventional static *in vitro* culture systems still occupy the absolute mainstream position. However, due to the substantial demand of biomedical-related industries, researchers have used new materials, new structures and advanced processing technologies to continuously innovate early embryo *in vitro* culture systems. At present, early embryo *in vitro* culture systems are mainly divided into conventional *in vitro* culture systems, pseudo-tubal/uterine dynamic culture systems, and co-culture systems.

### 4.2 Conventional *in vitro* culture system

A conventional culture system is a method in which embryos are cultured separately in a culture medium without adding other cells and their secretion factors. It is mainly divided into the droplet culture method [202.203], WOW (well-of-the-well) culture method [204.205], GO (glass oviduct) culture method 206 and microchannel static culture method 207. A droplet culture involves making 30-100 microlitre droplets in a petri dish, placing a small amount of embryos in it, and covering the surface of the droplets with paraffin oil. The WOW culture method uses a high-temperature method to melt the bottom of the orifice plate until small holes appear and strictly rinses with a special rinse to remove bubbles and harmful substances generated during the melting process 208. The accumulation of embryo-derived secretory factors in the microholes is beneficial for the development of the embryo. The GO culture method involves culturing embryos separately in a very small volume (1 microlitre) of a culture medium *in vitro* by simulating the shape of the fallopian tube. The microchannel static culture method is mainly to culture embryos in microchannels filled with a medium. The medium does not produce liquid flow, and the stress value of the embryos in the microchannel will be reduced. The channel wall can restrict the flow of autocrine and/or paracrine factors produced by embryonic cells so that these factors gather around the embryo as much as possible.

In conventional culture methods, oil is usually an indispensable material that can prevent the medium from evaporating, protect the medium from pollution 209, absorb toxic components 210 and facilitate observation. However, the oil film will also absorb lipophilic factors in the medium, and harmful substances will also enter the culture medium, which will have an adverse effect on the embryo [211.212]. S. Roh et al. 213 studied the method of mouse parthenogenetic embryo culture and designed a new oil-free microtubule culture system (MTC), which has a higher blastocyst formation rate than the conventional drip culture and oily MTC groups. In addition, some new materials have also been applied to conventional culture methods, which have a positive impact on embryos. Alginate is also an ideal three-dimensional extracellular matrix material for *in vitro* cell, tissue and embryo culture. Using alginate hydrogel as the test material, Zhao et al. 214 cultured bovine embryos after *in vitro* fertilization and parthenogenesis in an alginate encapsulation culture system (AECS), an alginate overlay culture system (AOCS) and a control culture system (covered with mineral oil). Finally, it was found that the AECS system and the AOCS system can promote the cell proliferation, elongation and differentiation of bovine embryos.

To date, conventional *in vitro* culture methods are still the mainstream *in vitro* culture methods in practical applications. These methods mainly consider the chemical and physical conditions during early embryonic development. However, its shortcomings are also very obvious. The developmental process of early embryos is a dynamic process. There are complex interaction processes between embryos and the tissue environment and even embryos. Long-term static culture is not a good choice; at the same time, due to the static state of the culture medium, the repeated manual pipetting and washing of embryos are required. Additionally, drastic changes in the temperature, pH, osmotic pressure, light and mechanical stress of the embryo environment during this process may adversely affect embryo development. At the same time, this process also has very high requirements with respect to the experience and technology of the experimental operators, and the success rate between different operators is very different.

### 4.3 Imitation fallopian tube/uterine dynamic culture system

To approach the fallopian tube/uterine environment as much as possible during the *in vitro* culture process to improve the success rate of an *in vitro* culture and to minimize the interference of human manipulation factors on the results, researchers have begun to develop various standardized and automated pseudo-fallopian tube/uterine dynamic culture systems. In addition to providing suitable physical and chemical conditions for early embryos, the pseudo-tubal/uterine dynamic culture system creates a dynamic environment close to the natural development of the living body on the basis of studying the reproductive mechanism. The early dynamic culture system was mainly a mechanical stimulation culture system 215. The embryos were cultured *in vitro* and subjected to similar mechanical stimulation in the mother. For example, Koji Matsuura et al. 180 developed the tilt culture system TECS (tilting embryo culture system), placed mouse embryo droplets on the tilt plate of TECS, and used a motor to drive the tilt plate to study the stimulation effect of the shear stress on early mouse embryos. Kim et al. 216 designed a new microfluidic culture system based on peristaltic mechanical stimulation, and the embryo was passed through channels with different contraction widths to simulate the peristaltic effect of peristalsis. The shear stress on the embryo on TECS is very similar to that in the fallopian tube. Studies have shown that mechanical stimulation can indeed increase the blastocyst development rate of early embryos. With the development of microfluidic technology, its inherent advantages are very suitable for pseudo-tubal/uterine dynamic culture systems, and it will soon be used to develop pseudo-tubal/uterine dynamic culture systems. Due to the current microfluidic technology system, the current pseudo-tubal/uterine dynamic culture system is mainly divided into a laminar flow microfluidic dynamic culture system and a droplet microfluidic dynamic culture system.

#### 4.3.1 Laminar flow microfluidic dynamic culture system

**Figure 14:**
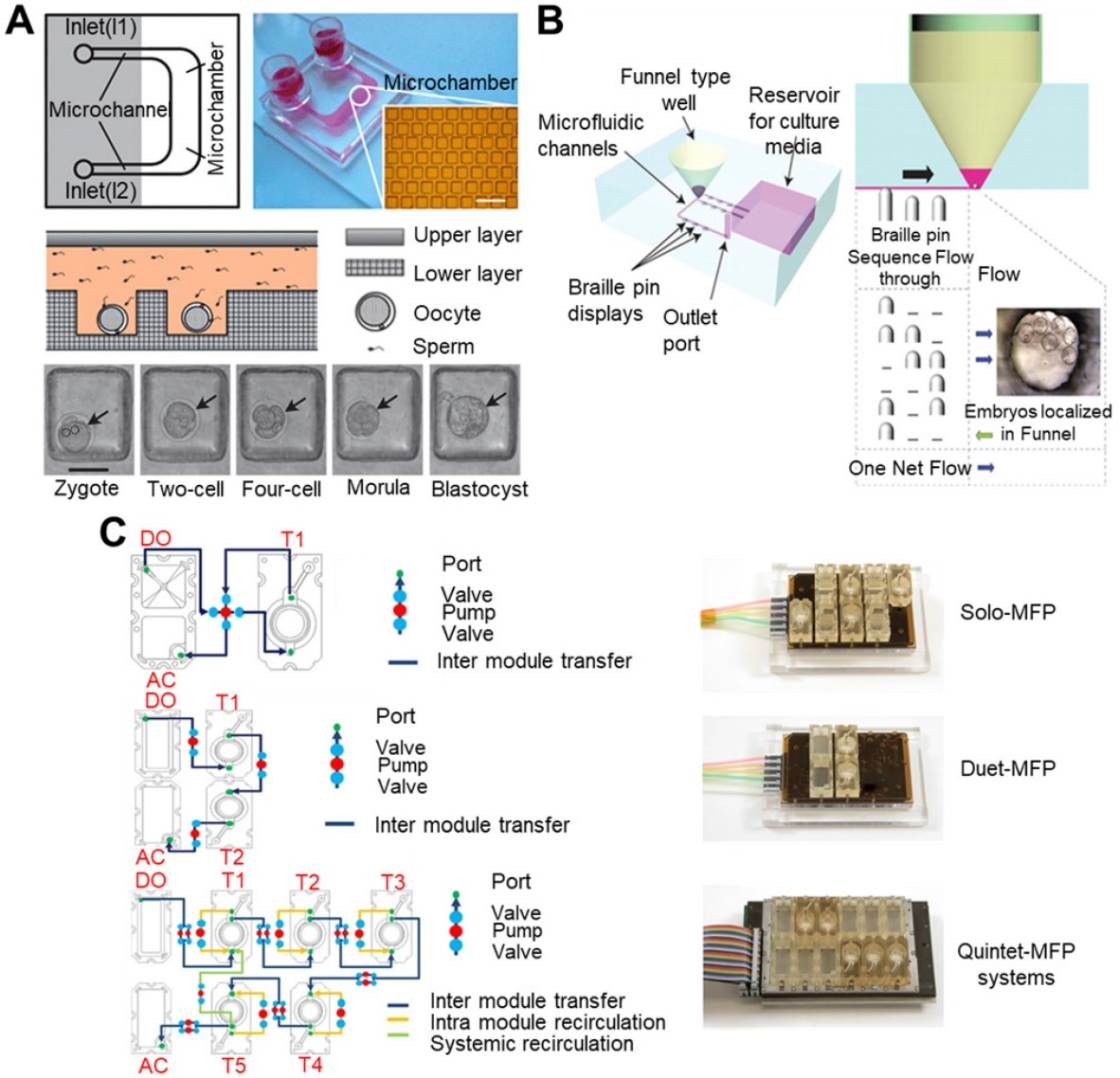
** The mechanical stimulation system** (A) A novel microwell-structured microfluidic device that integrates single oocyte trapping, fertilization and subsequent embryo culture. (B) A dynamic microfunnel embryo culture system would enhance outcomes by better mimicking the fluid mechanical and biochemical stimulation embryos experience *in vivo* from ciliary currents and oviductal contractions. (C) A microfluidic system that supports murine ovarian follicles to produce the human 28-day menstrual cycle hormone profile, which controls the human female reproductive tract and peripheral tissue dynamics in single-, dual- and multiple-unit microfluidic platforms. Adapted with permission from 217, copyright 2011 Royal Society of Chemistry, and 222, copyright 2018 Oxford University Press, and 223, copyright 2020 Nature Pub. Group.

Microfluidic technology is very consistent with the physiological requirements of the microenvironment required for embryo cultures. Microfluidic systems have been reported for embryo cultures under static and dynamic conditions. In a static culture, embryos are placed in a microchannel or culture chamber and cultured without a flowing medium. Under dynamic conditions, embryos are cultured in a flowing medium to simulate their physical environment in the body. Han et al. 217 designed a new type of microstructure microfluidic device with the functions of single oocyte capture, fertilization and embryo culture. Through the numerical simulation of the flow rate of the culture fluid, the capture of oocytes and the removal of debris at different microwell depths (50, 100, 150, 200 microns) were studied. As shown in **Figure 14A**, the 200-micron-deep wells reduced the maximum flow velocity near the oocytes by 97%; at the same time, the 200-micron-deep microwells were more capable of capturing and removing debris than the other three depths. Stephanie et al. 218 designed a microfluidic perfusion device using hydrodynamic traps, which can trap ovum cells in micropores. The micropores function as a culture chamber and can perform separate oocyte and embryo experiments. The device can perform high-resolution imaging at a uniform speed and control the temperature. Furthermore, the device can perfuse media, drugs, sperm and other substances in the microchannel, provide long-term incubation capabilities for longitudinal research, and avoid damage to cells when transferring cells.

Microfluidic technology creates a dynamic environment through the flow of culture media to simulate the fluid force and mechanical stimulation of the embryo in the oviduct environment. Miyoshi et al. 219 were the first to study the effect of pulsating mechanical vibration (PMV) on porcine oocyte maturation. The cumulus-oocyte complex was treated with a 20 Hz pulsating mechanical vibration (PMV). The results show that PMV-treated oocytes have a higher blastocyst rate. Regarding the vibration frequency, the frequency of cilia beating increases significantly after ovulation in the human isthmus and ampulla [165.220]. Second, the frequency of cilia beating is different, roughly distributed at approximately 5-20 Hz 221. Y.S. Heo et al. 222 designed a microfluidic culture system that uses microchannels as a channel for fluid to flow through the microfunnel where the embryo is located. The designed culture microfunnel minimizes the mechanical force generated by the fluid flowing through the channel. The microfunnel here positions multiple embryos together, provides a liquid storage container, buffers the biochemical exchange rate of liquid and fluid, and reduces the mechanical stimulation of the channel fluid to the embryo. The system uses a pin actuator to replace the traditional peristaltic pump to produce pulsation and peristalsis. The microchannel can generate liquid pulses with a periodic frequency of 0.135 Hz. Through simulation, the average shear force per unit area of the microchannel is 1.99×10^-3^ dyn/cm^2^, and the average shear force per unit area of the microfunnel is 3.55×10^-3^ dyn/cm^2^. Compared with static culture, dynamic funnel culture significantly improved the blastocyst rate and pregnancy rate, as shown in **Figure 14B**.

The method of culturing mammalian cells *in vitro* simulates the physical and chemical microenvironment. The typical medium composition is based on basic nutrients and some specialized factors, which are randomly mixed in a static culture environment. Cytokines and endocrine signals at the cell-cell and tissue levels are not integrated into the signalling pathway. Xiao et al. 223 designed a microfluidic platform (EVATAR) that integrates reproductive tract tissues and peripheral organs. This device can simulate the endocrine circulation of the female reproductive tract, ovaries, fallopian tubes, uterus, and cervix and realize the circulation of all organizations, as shown in **Figure 14C**. Gametes and early embryos are in close contact with epithelial cells when they are transported in the oviduct. Previous research on the *in vitro* culture of epithelial cells mainly focused on the monolayer culture of epithelial cells and the culture of fallopian tube tissue explants. Explant culture mainly uses epithelial cells to form ciliated beating vesicles. In monolayer culture, epithelial cells rapidly polarize and differentiate, becoming flat cells that lose their cilia and secretory ability.

**Figure 15:**
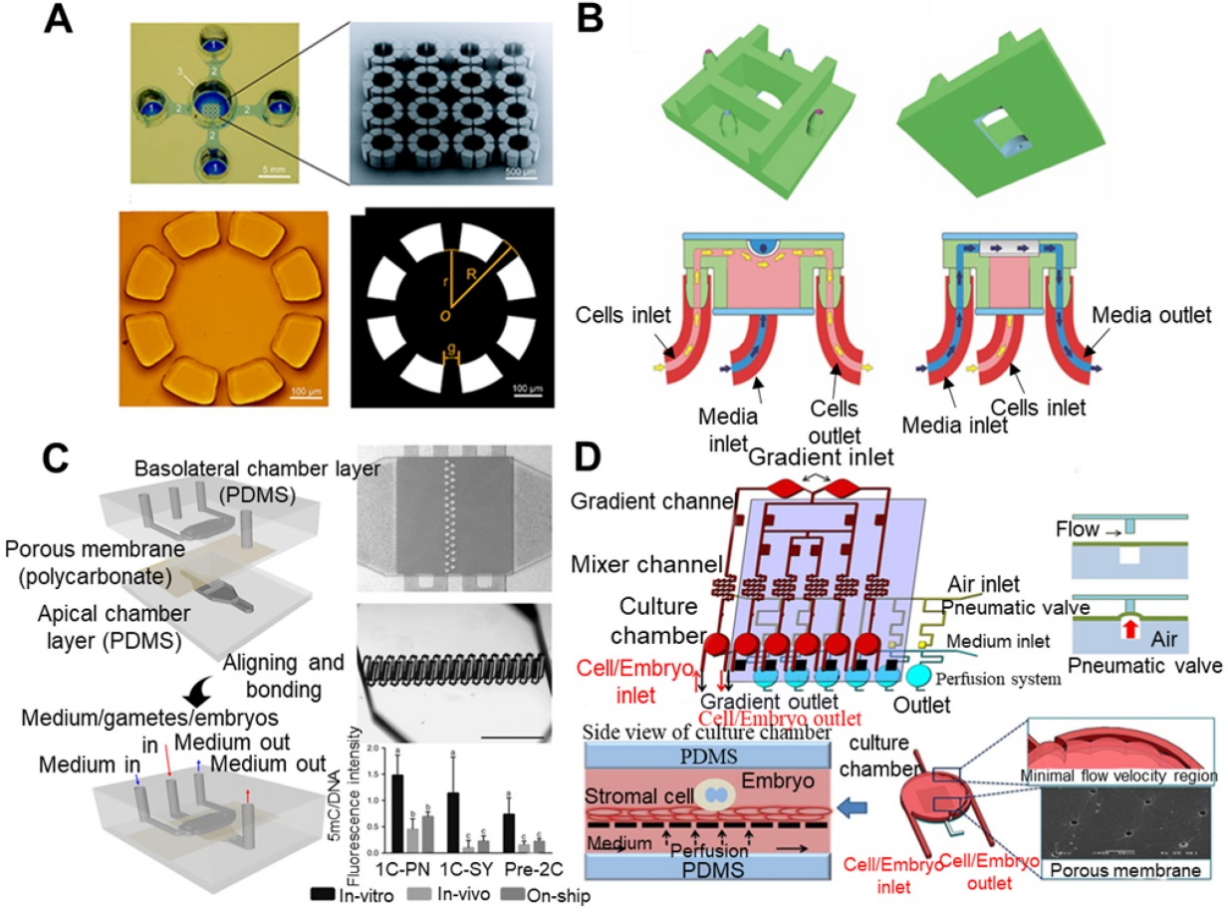
** Uterine-like co-culture microfluidic chip** (A) A novel microdevice that integrates each step of IVF, including oocyte positioning, sperm screening, fertilization, medium replacement, and embryo culture. (B) A U-shaped porous membrane three-dimensional fallopian tube model chip. (C) An oviduct-on-a-chip platform to better investigate the mechanisms related to (epi)genetic reprogramming and the degree to which they differ between *in vitro* and *in vivo* embryos. (D) A microfluidic chip co-cultured with embryos and endometrial stromal cells. Adapted with permission from 226, copyright 2011 American Chemical Society, and 227, copyright 2017 Royal Society of Chemistry, and 228, copyright 2018 Nature Pub. Group, and 229, copyright 2016 Elsevier Sequoia.

At present, there are some microfluidic devices combined with pseudo-tubal dynamic culture and co-culture systems. Paula et al. 224 used early pig embryos as the research object. When the culture distance between embryos ranged from 81 μm to 160 μm, the fertilized ovum satisfactorily developed to the blastocyst stage. As the distance increased, the blastocyst development rate decreased significantly, verifying that the diffuse paracrine/autocrine factors produced by early pig embryos can promote *in vitro* culture between embryos. Michelle et al. 197 proved that preimplantation mouse embryos can produce factors that stimulate culture, and the embryos will stimulate each other. When more embryos are implanted in the same droplet, the more embryos will reach the blastocyst stage. When low-quality and high-quality embryos are cultured in the same droplet, the two will interact. High-quality embryos will have a positive effect on low-quality embryos. In contrast, low-quality embryos will have an adverse effect on high-quality embryos. U. Sanmee et al. 225 found that by co-culturing different proportions of semi-destructive and intact 4-cell embryos, the blastocyst rate of the former was increased, while the latter exhibited no significant change, verifying that high-quality embryos have a beneficial effect on low-quality embryos. Co-cultivation between embryos is also a way to improve the quality of embryos *in vitro*. Xie Lan et al. 226 designed a new type of microfluidic device in which each step of *in vitro* fertilization is integrated into the device, including ovum positioning, sperm screening, fertilization and other steps. An array of 4×4 cells is designed. Each cell is a circular fence structure composed of 8 pillars. The inner diameter of the cell is 400 microns to capture 1 to 4 oocytes, and the gap between the pillars is 50 microns to facilitate the passage of sperm, as shown in **Figure 15A**. Connecting the 4 linear channels with the oocyte positioning area can effectively screen sperm, quickly replace the culture medium, remove unattached and dead sperm, and provide a gentle and rapid medium replacement method. The damage to the embryo caused by the embryo from one droplet to another is avoided. Through dynamic monitoring of embryo development, the microfluidic chip can successfully perform *in vitro* fertilization, and the growth rate of embryos is similar to that of conventional *in vitro* fertilization (p>0.1).

Ferraz et al. 227 designed a U-shaped porous membrane three-dimensional fallopian tube model chip, including a basolateral perfusion chamber that simulates blood circulation and an independent apical perfusion chamber that simulates fluid movement in the fallopian tube lumen. The system can simulate endocrine changes in the body and promote an increase in beneficial substances and the removal of harmful substances. The use of porous membranes ensures that epithelial cells are cultured at the air-liquid interface, maintaining cilia beating and epithelial secretion. This device can effectively avoid multiple sperm fertilization and parthenogenesis, as shown in **Figure 15B**. Furthermore, Ferraz et al. 228 found that fallopian tube model chips made of conventional materials produce toxic substances (phthalates and ethylene glycol) during cell culture, which affect early embryonic development. PDMS has good biocompatibility. Ferrza used PDMS materials to further optimize and design a microfluidic fallopian tube chip platform, which contains two independent and perfusable compartments, which are separated by a porous membrane. The apical chamber continuously perfuses the fallopian tube epithelial cell layer with IVF and IVP devices, and the chamber is also designed with a column for capturing oocytes and embryos. The basal compartment is used to simulate the changes in circulating hormones that occur during ovulation. This simulated fallopian tube platform introduces changes in the culture conditions and the oestrus cycle at the same time, which can better simulate the environment in the body. By using the immunofluorescence labelling method to detect changes in the global DNA methylation status, the embryonic development ability can be revealed after the activation of the embryonic genome. The first genetic comparison of the 4.8.16-cell and embryonic stages of bovine embryo development found that compared with standard IVP conditions, the platform can improve the delay of zygotic transcriptome activation, as shown in **Figure 15C**. Chang et al. 229 designed a microfluidic chip in which embryos and endometrial stromal cells were co-cultured, as shown in **Figure 15D**. A semicircular arc-shaped microstructure was designed on the side wall of the culture chamber. The embryo uses the microstructure to generate mechanical stimulation to the embryo under the drive of fluid, which simulates the cilia of the embryo in the fallopian tube. In addition, the chip simulates hormone changes during the menstrual cycle and creates a gradient channel that automatically generates different concentrations of steroid hormones. In addition, multiple culture chambers are connected to each other through microchannels to produce a group culture effect, which is conducive to the exchange of autocrine and paracrine factors between embryos. In the culture room, endometrial stromal cells are cultured on the porous membrane, and fresh culture fluid is delivered to the cells on the top and bottom, effectively avoiding the adverse effects on the embryo caused by apoptosis. Experimental results showed that the blastocyst development rate of 67±10.05% under the co-culture condition was higher than that of 49±8.19% under the non-co-culture condition. The co-culture condition was more conducive to the culture and development of the culture. In short, with the future development of microfluidic technology, the integration of co-culture technology with new culture materials and culture devices can create an *in vitro* culture environment closer to that of a living body for early embryos and promote the development of *in vitro* culture technology for early mouse embryos.

#### 4.3.2 Droplet-type microfluidic dynamic culture system

When *in vitro*, laminar flow microfluidic devices mainly provide a microchannel, microstructure and continuous flow culture liquid, while fluid mechanics technology is used to manipulate cells and simulate the physiological microenvironment experienced *in vivo* 230. However, laminar flow microfluidic devices also have disadvantages. For example, the culture method may cause pollution problems, and it is difficult to manipulate and observe a single cell or several cells in a continuous flow channel. DMF (digital microfluidic) platform has become a good alternative. The DMF platform can bypass channels, pumps and valves to control fluid movement and avoid the problem of blockage caused by this effect 231. In its most popular way, DMFs can use electric fields (including EWOD and DEP) on the electrode array to process microscale discrete droplets 232. The DMF platform has become a very good tool for manipulating trace reagents and target cells in a single droplet and has been widely used in the field of cell manipulation (e.g., cell culture 233-236, cell sorting and concentration 237-240, and single-cell manipulation and analysis 241-244) in recent years. The DMF platform has the advantages of low reagent usage, a simple structure, and a high degree of automation 245. Irena et al. designed the first chip platform for complete mammalian culture driven by DMFs, integrating all the steps (seeding, growing, separating and reseeding) of mammalian cell culture into the DMF platform to realize the automated cultivation of mammalian cells 233.

**Figure 16:**
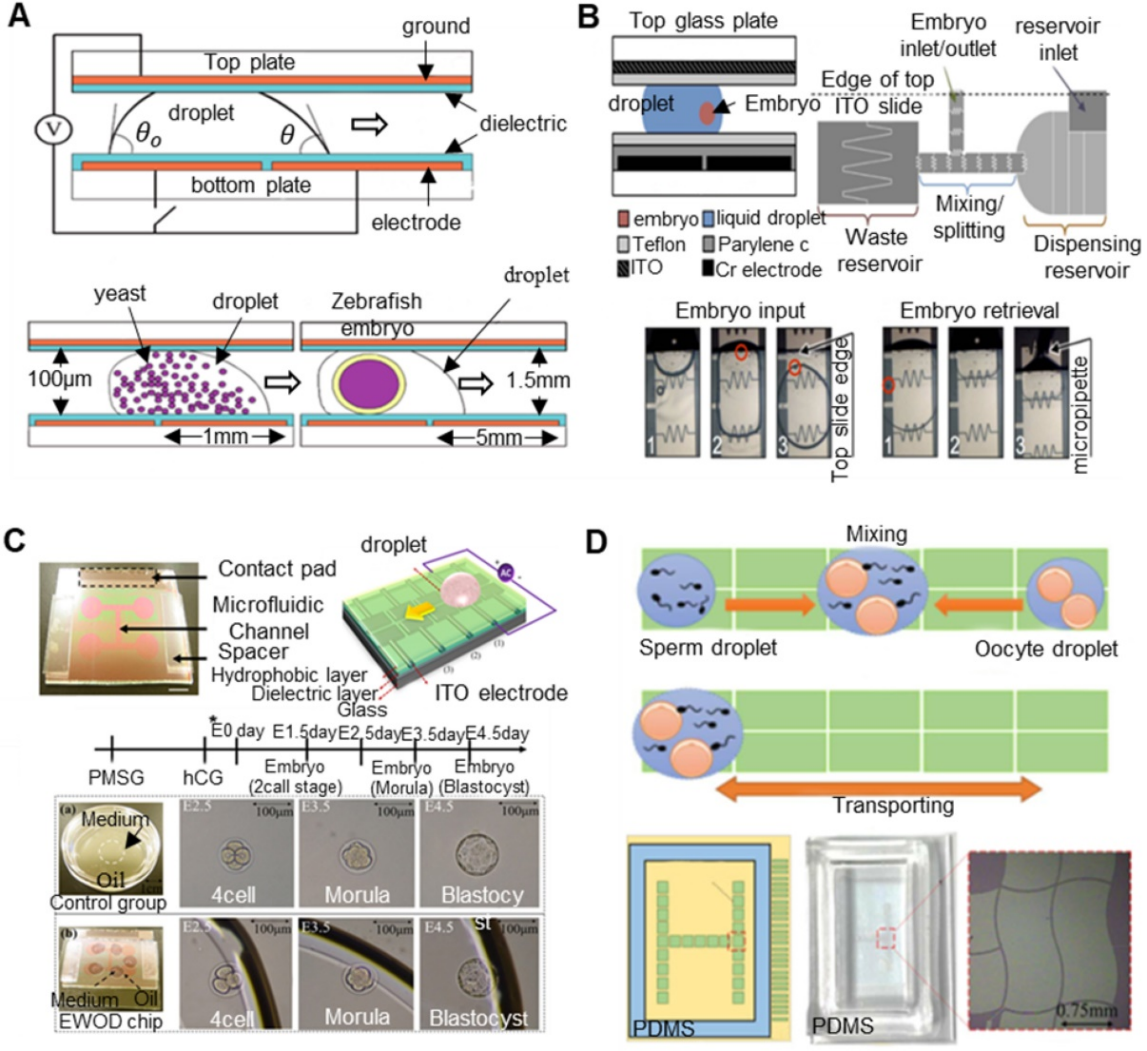
** DMF-style dynamic cultivation system.** (A) Dual-plate digital microfluidic device EWOD technology. (B) Digital microfluidic equipment to automate the processing of vitrified frozen embryos. (C) A microfluidic microchannel system for IVF is considered to provide an improved *in vivo*-mimicking environment to enhance the development of an embryo culture system before implantation. (D) A new DMF chip design with a PDMS ring has solved the problem of gas exchange during long-term embryo culture. Adapted with permission from 247, copyright 2009 Royal Society of Chemistry, and 248, copyright 2014 Public Library of Science, and 249, copyright 2015 Public Library of Science, and 250, copyright 2015 IEEE.

The EWOD (electrowetting on dielectric) system is a droplet driving mechanism in the DMF platform and is one of the most advanced methods for manipulating droplets. The EWOD system is derived from the electrowetting phenomenon that changes the contact angle of the liquid. By applying an AC voltage to the electrode directly below the surface of the substrate, the wettability of the droplet surface is changed locally, and the attraction that drives the movement of the droplet is generated. It is usually quantified by the degree of contact angle reduction. The change in the contact angle in the EWOD system is generally defined by the Young-Lippmann equation 246, and the contact angle is usually related to factors such as the operating frequency, dielectric constant, and droplet conductivity. This system can easily create, transfer, separate and combine micron- and nanometre-sized droplets, and its operation is stable and repeatable 245. Son et al. 247 used double-plate digital microfluidic device technology to transfer live yeast droplets and zebrafish embryo droplets and mixed the two droplets after transmission to achieve dechlorination. The transferred zebrafish embryos developed normally and hatched. This finding proves that the DMF platform can manipulate cells as well as larger embryos to maintain their viability, as shown in **Figure 16A**. The DMF platform can transport oocytes or embryos to a designated location for *in vitro* fertilization of oocytes or the chemical and mechanical treatment of embryos. Derek G. Pyne et al. 248 used digital microfluidic equipment to automate the processing of vitrified embryos, as shown in **Figure 16B**, including droplet delivery, mixing, distribution and splitting. Mouse embryos were placed in droplets that function as capillaries, and a single mouse embryo was transported through the DMF platform during the complete vitrification process. The embryo survival rate and development rate obtained by this device are equivalent to those obtained manually. This method has the advantages of automatic operation, the generation of a cryoprotectant concentration gradient and the feasibility of loading and retrieving embryos. Huang et al. 249 described a new type of digital microfluidic device with the EWOD system, as shown in **Figure 16C**, to cultivate mammalian embryos in a single droplet. Using the EWOD system to provide a dynamic fluid environment, this device provides a discontinuous microfluidic environment to simulate the *in vivo* embryonic development environment, promoting mouse embryo cleavage and the development to the preimplantation stage. However, this system cannot provide stable embryo movement speed; that is, the embryo movement speed in the droplet is random. Huang further designed a new type of circular DMF chip 250, as shown in **Figure 16D**, with the functions of *in vitro* fertilization and *in vitro* culture. In the experiment, HTF droplets containing male and female mouse gametes were mixed with DMF-treated droplets to form fertilized ova. The fertilized embryos were dynamically cultured and transported between electrode pairs at a certain interval. The *in vitro* fertilization rate of the DMF platform was 8.7% higher than that of traditional petri dishes, and the number of embryos that have developed to 8-cells was similar. The ring-shaped DMF chip solves the problem of gas exchange during embryo culture. Embryos in dynamic culture develop faster than those in static culture.

### 4.4 Conclusion for early embryo *in vitro* culture

In related research regarding the *in vitro* culture of early embryos, researchers have been constantly exploring the reproductive mechanism of early embryo development and have found that there are many complex factors affecting early embryo development. On the one hand, based on cost, operability and other factors, the conventional *in vitro* culture method of static culture still occupies a dominant position in many biological and medical laboratories. To improve this technology, some studies have introduced new culture materials to improve the culture effect and tap its potential. On the other hand, the reproductive mechanism of early embryos shows that dynamic culture technology is the most natural form of a culture system and will be the future development direction of *in vitro* culture technology. A large number of studies have focused on the tubal/uterine dynamic culture system. With the development of microfluidic technology, dynamic culture technology has been greatly promoted. By simulating organ-like structural microenvironments and dynamic biochemical/mechanical stimulation processes, various complex culture systems have been constructed to approach the natural development environment of early embryos, which has indeed brought about a substantial increase in the embryo development rate and has shown great advantages. There is no problem in the theoretical verification. However, considerable effort is required to replace traditional *in vitro* culture technology with practical systems. The system is too complex, the processing cost is high, and the reusability and ease of use are not strong. Cost reduction and moving these advanced technologies from the laboratory to industry are key problems to be solved in the future.

## 5. Conclusion and prospect

**Figure 17:**
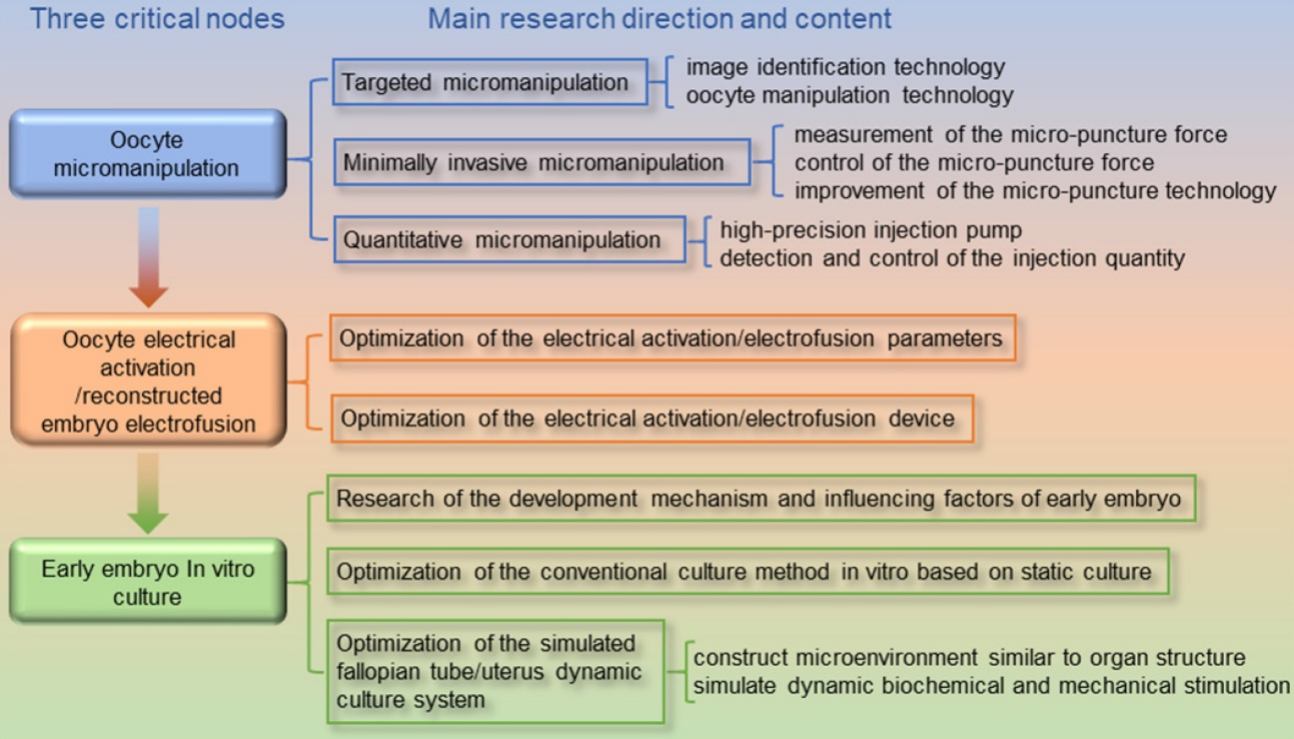
Critical nodes in embryo engineering technology and their main research contents.

From the perspective of engineering, this article presents the three high-risk nodes that most affect the success rate of the entire process of embryo engineering technology according to its current status and development trend, namely, oocyte micromanipulation, oocyte electrical activation/reconstructed embryo electrofusion and *in vitro* culture of early embryos. Researchers have started from these three critical nodes and developed numerous novel machine-assisted technologies to improve the effect. The main research content is shown in **Figure 17**. However, machine-assisted technology has never shown much advantage in terms of the core index, i.e., the success rate. This reason requires us to have a more in-depth understanding about the technical process of embryo manipulation. The embryo engineering technology process is first a life development process, with its own laws of life activities, and the operation technology without an in-depth analysis of its internal physiological mechanism cannot guarantee to maximize its advantages. Second, embryo operation technology is a sequential operation process, and its influencing factors are very complicated. All the factors of the previous operation node will have an impact on the following steps, and the improvement effect of some factors on a single node will be diluted by other nodes. In future related research, we should no longer pursue the ultimate improvement of certain aspects of technology in one aspect. Instead, we should have a systematic concept. First, we should find the weak bottleneck in the most critical nodes that affects the success rate to make breakthroughs, then take the process as a whole, conduct systematic analyses and repeated corrections, and finally achieve a substantial increase in the overall success rate of machine-assisted embryo engineering technology. The details are as follows:

In related research on oocyte micromanipulation, although researchers have invested substantial energy, they have tried to use the high sensitivity, high positioning accuracy, and high stability of machines to compensate for deficiencies in the stability of manual operation to achieve an improved success rate. However, the effect of machine-assisted micromanipulation is inadequate, and the reasons may be as follows. First, the existing micromanipulation technology lacks scientific theoretical guidance. The selection of existing micromanipulation strategies (injection path, angle, etc.) is basically based on manual operation experience. Second, researchers generally regard the target oocyte as a cortical membrane model. Research on oocyte damage has basically focused on the zona pellucida, but the internal structures of oocytes are often ignored. However, these internal microstructures, such as the nucleus and skeleton, are more important to oocytes. In future research, a guiding strategy for robotic oocyte micromanipulation should be established to provide theoretical guidance for micromanipulation instead of continuing to use the empirical conclusions of manual manipulation. The evaluation mechanism of the degree of damage in oocyte micromanipulation should be established and fed back to the experiment to help find the best operation mode and parameters.

In research on oocyte electrical activation/reconstructed embryo electrofusion, we should first consider the characteristics of oocyte electrical stimulation, combine microfluidic technology and micromanipulation technology, and develop a new precise electrical stimulation device suitable for oocyte electrical activation/reconstructed embryo electrofusion. Second, we should deeply reveal the physiological mechanism of oocyte electrical activation/reconstructed embryo electrofusion, study the effect of the different parameters of electrical stimulation signals on oocytes, and realize the optimization of electrical stimulation parameters. Through the combination of these two aspects, the best electrical stimulation effect can be obtained by trial and error correction.

In the study of the *in vitro* culture of early embryos, although researchers have constructed a variety of organ-like structural microenvironments and complex culture systems of dynamic biochemical/mechanical stimulation processes, they have brought a substantial increase in the rate of embryo development. However, there is still a long way to go for all kinds of systems to replace traditional *in vitro* culture technology. The existing systems are too complex, the processing cost is high, and the reusability and ease of use are not strong. To make it out of the laboratory, future work should focus on system improvement through the introduction of new processing technology, new materials and new structures, reduce the cost of products, strengthen modular design, increase the reuse rate of products and reduce the cost of consumables. Only in this way can actual operators use and promote the development of related industries.

In addition, although a large number of new technologies have been developed to improve the various nodes of embryo manipulation technology, how can we evaluate its effect? As the influencing factors of embryo engineering technology are too complicated, it is still very difficult to evaluate them. At present, research related to embryo engineering technology basically regards the embryo development rate/survival rate after surgery as the evaluation standard of the pros and cons of the new technology. In reproductive medicine, the final effect of the entire operation process can only be evaluated in the last *in vitro* culture link based on experience or image data, combined with the static morphological quality assessment system and the jet lag embryo culture detection system. Generally, this kind of evaluation system is very rough, empirical and subjective and is too strong to meet the developmental needs of embryo manipulation technology. Quantitative monitoring methods should be introduced for the entire process of embryo engineering technology, combined with complex models for predictive analysis, so that problems can be identified. Only by performing targeted improvements can machine-assisted embryo manipulation technology achieve a substantial increase in the overall success rate, show its superiority over manual operations, and promote technological upgrading. Ultimately, as a basic technology, this technology gives a considerable boost to related biomedical research and industry.

## Figures and Tables

**Table 1:** Oocyte manipulation methods

	Manipulation methods	Advantage	Disadvantage	References
Contact methods	Microcapillary negative pressure suction	Simple structure, easy to combine with existing micromanipulation systems	Requiring a high-precision source and can only fix one oocyte at a time	50,51
	“Microtrap” device	Batch processing oocytes	Size of the microtrap will affect the fixing effect	34,52
	Combination of negative pressure suction and a “microtrap”	Batch processing oocytes with a good fixation effect	Complex structure	53
Noncontact methods	Microfluidic	Less damage to oocytes	Complex manipulation and low efficiency	35,54-58
	Dielectrophoresis	High-precision and fast response	The structure is complex and the electric field may affect other equipment	59-64
	Optical tweezers	Not limited by the operating space	Operating force is small, with potential heat damage to oocytes	65-74
	Magnetic field	High precision	The complex structure and magnetic field may affect the manipulation of other equipment	75-79

**Table 2:** Measurement of the micropuncture force

	Measurement methods	Advantage	Disadvantage	References
Direct measurement	Piezoresistive force sensor	Simple structure, easy to combine with existing microinjection systems	High assembly precision and easily affected by environmental temperature changes	76,77
	Capacitive force sensor	Large force measurement range and high accuracy, multi-axis force information can be measured, not affected by environmental temperature changes	Complex structure and high cost	78-81
	Piezoelectric force sensor	Large measurement range, high bandwidth, small size and high power density	Susceptible to ambient temperature change and charge leakage will lead to signal drift	82-84
	Optical force sensor	High sensitivity, anti-electromagnetic interference, no hysteresis, and no limitation of the operating space	Susceptible to light reflection or refraction, and has potential heat damage to cells	85-87
Indirect measurement	Vision-based method	Not limited by operating space and without any damage to cells	Low precision	88-90
	Calculation method	Can detect forces that are not easy to measure directly	Complex structure and low precision	91,92
	Actuator input method	Suitable for online measurement and control, without additional equipment	Poor precision	93,94

**Table 3:** Application of electrical activation in parthenogenesis

Research objects	Research contents	Effects and conclusions	References
Pig	To study the influence of the number, frequency and interval of electrical stimulation pulses on the parthenogenesis of oocytes.	Neither the pulse frequency nor the pulse interval affects the rate of pronucleus formation. Multiple electrical pulse stimulation can achieve a faster development speed and a higher cleavage rate, the optimal electrical stimulation parameters: three DC pulses of 1.0 kV/cm for 50 μs, 5 min apart.	131
	Study of the parthenogenesis of a pig comparing the EA and CA.	The cleavage rate of the EA was higher than that of the CA. The optimal electrical stimulation parameters: three DC pulses of 1.0 kV/cm for 50 μs.	132, 133
	Study of the parthenogenesis of a pig using a combination of EA and CA.	The electrically activated pig oocytes can obtain a higher blastocyst formation rate by anisomycin. The optimal electrical stimulation parameters: a direct current pulse of 1.5 kV/cm for 60 μs.	134
Cow	Study of bovine parthenogenesis, using a combination of the EA and CA.	The optimal electrical stimulation parameters: two DC pluses of 1.75 kV/cm for 30 μs. The activated oocytes are treated with 6-dimethylaminopurine (6-DMAP). The cleavage rate can be comparable to IVF.	135
Mouse	Study of the parthenogenesis of mouse comparing the EA and CA.	Mouse oocytes could develop into blastocysts after EA or CA, but the formation rate and mass of the blastocysts after EA were significantly higher than that after CA, and the combined use of activators had no further positive effect on the development before implantation.	136

**Table 4:** Application of electrical activation in ICSI

Research objects	Research contents	Effects and conclusions	References
Rabbit	Electrical stimulation was performed on rabbit oocytes 10 minutes before ICSI.	Electrically stimulated oocytes had a higher rate of activation and subsequent development (implantation rate, pregnancy rate, live birth rate). The optimal electrical stimulation parameters: a single pulse of 1.25 kV/cm for 100 µs.	137
	The effect of electrical stimulation at different periods on the ICSI of rabbit oocytes was studied.	Rabbit oocytes that receive electrical stimulation before ICSI will have a higher rate of blastocyst development. The optimal electrical stimulation parameters: a single DC pulse of 2.5 kV/cm for 25 μs before ICSI.	138
Pig	The effect of electrical activation on the ICSI of porcine oocytes was studied.	The cleavage rate and blastocyst development rate of the electrically activated group were significantly higher than the IVF group and the non-activated ICSI group, the optimal electrical stimulation parameters: a single DC pulse of 1.26kV/cm for 30 s, 30 min after ICSI.	139
Human	Electrical stimulation of unfertilized oocytes 24 hours after the ICSI.	Oocytes after electric stimulation can be normally fertilized and complete early embryo development, and repeated electric stimulation can significantly improve subsequent embryo development. The parameters of electric stimulation: 1.35∼1.5 kV/cm, 40∼60 μs.	140
	Divide a large number of oocytes after the ICSI into two groups. One group receives electrical stimulation, and the other group serves as a control group without electrical stimulation.	The fertilization rate of the electrical stimulation group was significantly higher than that of the control group, and the degeneration rate of oocytes in the two groups was similar. The electrical stimulation parameters: a double-square DC pulse of 2.6∼2.8 kV/cm for 50 μs.	141
	The man suffered from round-head spermatozoa, and the oocytes cannot be fertilized after the ICSI. Electrical stimulation was applied to the oocytes 30 minutes after the ICSI.	The oocytes after electrical stimulation can be fertilized normally and develop to 4-8 cells, and then transplanted into the female uterus, and finally a healthy baby is successfully produced. The electrical stimulation parameters: a single DC pulse of 0.75 kV/cm for 50 μs.	142

**Table 5:** Application of electrofusion in SCNT

Research objects	Research contents	Effects and conclusions	References
Pig	Analyse the effects of the mature age and activation conditions of oocytes on the SCNT of pig oocytes.	The longer the oocytes were cultured, the higher the maturation rate. Additionally, the oocytes were more easily activated. The combined treatment of additional electrical stimulation and 6-DMAP after electrofusion can effectively improve the blastocyst formation rate. The electrofusion parameters: a single DC pulse of 1.5 kV/cm for 30 μs.	143
	When studying the SCNT of pig oocytes, different electrofusion pulses were used, and the fusion was divided into 3 groups for processing.	The optimal electrofusion parameters: a single pulse of 1.1 kV/cm for 30 μs. The fused oocytes treated with cytochalasin B had a higher blastocyst formation rate.	144
	The effects of different electrofusion parameters on the manual cloning of pig oocytes were studied.	The optimal electrofusion parameters: a single pulse of 1.0 kV/cm for 9 μs.	145
Cattle	Influence of different activation times on embryonic development after electrofusion.	The blastocyst development rate of reconstructed oocytes activated 3∼5 h after electrofusion was significantly higher than that of reconstructed oocytes activated immediately after electrofusion. The electrofusion parameters: a single DC pulse of 1.8 kV/cm for 20 μs.	146, 147
	The effects of different electrofusion parameters and different shapes of somatic cells on embryonic development were studied.	The round smooth somatic cells are better than the prototype rough somatic cells. Donor cells with a diameter of 15~25 μm are better than others. The optimal electrofusion parameters: two DC pulses of 2.5 kV/cm for 10 μs.	148
	The effects of different electrofusion parameters on buffalo oocytes cloned by handmade cloning were studied.	The optimal electrofusion parameters: a single pulse of 3.36 kV/cm for 4 μs.	149,150
Goat	Electrofusion oocytes of a goat.	The treatment of mammary gland epithelial cells with 100 mg/ml phytohemagglutinin before fusion can improve the fusion rate between the mammary gland epithelial cells and oocytes. The optimal electrical stimulation parameters: two DC pulses of 2.2 kV/cm for 10 μs.	151
	The effects of different electrofusion parameters on the oocytes of cloning goats were studied.	The optimal electrofusion parameters: a double electrical pulse of 2.2 kV/cm for 10 μs.	152
Mouse	Effects of unipolar pulses (UPs) and bipolar pulses (BPs) on the electrofusion of mouse SCNT embryos.	The experimental results showed that the bipolar pulse could effectively reduce the death of the embryos, and the electrofusion rate of the BPs was three times that of the UPs.	153
	Electrofusion of 2-cell embryos to obtain single-cell tetraploid embryos.	The optimal electrical stimulation parameters: a single pulse of 3.5 kV/cm for 35 μs.	154
	Human-mouse heterogeneous hybridoma cells were produced based on cell electrofusion technology, and the effect of electric field direction on fusion was studied.	The fusion yield can be increased by firing pulses at the cells in both vertical directions.	155

**Table 6:** The influence of chemical and physical factors on early embryos in the culture environment

Factors	Effects and conclusions	References
**Chemical Factors**		
Water	Water is an important component of the culture fluid. Nutrients and metabolites are absorbed and excreted by the oocytes by dissolving in water. The quality of water directly affects the effect of *in vitro* culture, and the small quantity of impurities can also affect the survival and development of oocytes.	168
Inorganic salt ion	In the process of embryo culture, inorganic salt ions play a role in regulating the osmotic pressure, maintaining the potential difference, and regulating the pH value in the medium. An ion concentration that is too high is not conducive to the development of the embryo.	169
Energy matter	Glucose, pyruvate, lactate, etc. are energy substances required for embryo cultures *in vitro*. Early embryos at different developmental stages require different energy substances.	170
Amino acid	Amino acids are the basic substances that make up protein, DNA, and RNA, as well as nutrients for early embryos. It can regulate the osmotic pressure, participate in trophoblast differentiation and the formation of basement membrane between endo and ectoderm, and promote the normal development of early embryos.	171
Vitamins	The effect of vitamins on embryonic development is to improve the energy metabolism-related enzyme activity, promote the embryo's absorption of glucose, and further promote the effect of early embryonic development *in vitro*.	[172.173]
PH value	To provide a good environment for embryos, early embryos need to simulate the acid-base environment in the living body when they are cultured *in vitro*. Most of the culture media rely on [*HCO_3_^—^*] and [*H_2_CO_3_*] as buffer materials to adjust the pH value between 7.2-7.4.	174
**Physical Factors**		
Temperature	Changes in temperature directly affect the physiological metabolism of early embryos. Excessive temperature causes heat stress on embryos, which affects the decrease of key enzyme activities in the embryo and the abnormal expression of important survival genes, resulting in a decrease in the blastocyst development rate and embryo quality. A temperature that is too low will lead to the slow development of early embryos and even a developmental block.	175
Gas phase	The gas phase condition is one of the important factors of the microenvironment during the *in vitro* culture of early embryos. *CO_2_* and *O_2_* are two gas phase factors that have a greater impact on the embryo *in vitro* culture. *CO_2_* can stabilize the pH, participate in the synthesis of the early embryonic protein and nucleic acid, and regulate metabolism. *O_2_* supports the metabolism of embryonic development, and the embryonic blastocyst development rate is increased under a relatively low oxygen environment, which is conducive to embryo development.	176-178
Humidity	Avoid evaporation of the culture solution, ensure proper humidity conditions, and maintain the ion concentration and osmotic pressure in the culture solution.	179
Force stimulation	The early embryo will be subjected to the compression stress produced by the oviduct wall and the fluid shear stress produced by the oviduct fluid. When the shear stress exceeds 12 dyn/cm^2^, the blastocyst will die within 12 h. Reasonable mechanical stimulation plays an important role in embryonic development.	180
Illumination	Light directly or indirectly affects embryos by increasing active free radicals, inducing gene transcription or increasing breakdown products in the culture medium. Light affects cell division, causes DNA damage and impaired implantation ability of embryos. Light is harmful to the development of mammalian embryos, and the degree of damage depends on the light intensity, wavelength, irradiation time and the light sensitivity of the embryo.	181
Osmotic pressure	The specific medium used for early embryo development has certain requirements on the osmotic pressure, which is generally stable in the dynamic range of 280 ~ 310 mOSM. The osmotic pressure is adjusted mainly by the concentration of inorganic salt ions (mainly Na and k), and then the ion channels on the cell membrane can control the in and out of ions. An osmotic pressure that is too high or too low has a negative impact on embryo development.	182

**Table 7:** Co-cultivation effects of embryos of different species and various cells

Co-cultured cell types	Effects and conclusions	References
Porcine oocytes and ovarian cortical cells (POCCS).	Improve the maturation quality of pig oocytes and the development rate of blastocysts significantly.	192
Bovine early embryos and bovine oviduct epithelial cells (BOEC).	Provides a favourable culture environment for embryos and promotes the development of bovine embryos.	193
Porcine oocytes and canine oviduct cells (COS).	Improve the maturation of oocytes *in vitro* and have a positive impact on the development of later embryos.	194
Mouse embryo and mouse uterine epithelial cells.	Co-culture can promote embryo development and blastocyst formation.	195
Mouse embryo and mouse endometrium.	Co-culture of embryos and endometrium is beneficial for increasing the rate of blastocysts, activating specific paracrine factors in time, and improving the possibility of embryo implantation.	196
Human embryo and human endometrial epithelial cells (EEC).	It is beneficial for human blastocysts, can induce the secretion of embryonic paracrine factors, absorb harmful substances, and change the metabolism in the medium.	197
