# Supplementary material for: Recent advances in critical nodes of embryo engineering technology
Source: Theranostics. 2021 May 25;11(15):7391–424. doi: 10.7150/thno.58799 (PMC8210615; doi:10.7150/thno.58799)
Supplement: Supplementary file 1 — Supplementary figures and tables. [file thnov11p7391s1.zip › Supplementary material/Figure copyright/14a.pdf]

Copyright Clearance Center Marketplace™

Order Confirmation

Thank you, your order has been placed. An email confirmation has been sent to you. Your order license details and printable licenses will be available within 24 hours. Please access Manage Account for final order details.

This is not an invoice. Please go to manage account to access your order history and invoices.

CUSTOMER INFORMATION

Payment by invoice: You can cancel your order until the invoice is generated by contacting customer service.

Billing Address

MingWei Gu
Suzhou of Jiangsu province
Suzhou
China

+86 18112585994
mwgu@stu.suda.edu.cn

Customer Location

MingWei Gu
Suzhou of Jiangsu province
Suzhou
China

PO Number (optional)

N/A

Payment options

Invoice

PENDING ORDER CONFIRMATION

Confirmation Number: Pending

Order Date: 23-Apr-2021

1. Lab on a chip

0.00 USD

**Order license ID**	Pending	**Publisher**	ROYAL SOCIETY OF CHEMISTRY
**ISSN**	1473-0189		
**Type of Use**	Republish in a journal/magazine	**Portion**	Chart/graph/table/figure

LICENSED CONTENT

**Publication Title**	Lab on a chip	**Country**	United Kingdom of Great Britain and Northern Ireland
**Author/Editor**	Royal Society of Chemistry (Great Britain)	**Rightsholder**	Royal Society of Chemistry
**Date**	01/01/2001	**Publication Type**	e-Journal
**Language**	English	**URL**	http://www.rsc.org/loc

REQUEST DETAILS

**Portion Type**	Chart/graph/table/figure	**Distribution**	Worldwide
**Number of charts / graphs / tables / figures requested**	2	**Translation**	Original language of publication
**Format (select all that apply)**	Electronic	**Copies for the disabled?**	No
**Who will republish the content?**	Publisher, not-for-profit	**Minor editing privileges?**	No
**Duration of Use**	Life of current edition	**Incidental promotional use?**	No
**Lifetime Unit Quantity**	Up to 499	**Currency**	USD
**Rights Requested**	Main product		

NEW WORK DETAILS

**Title**	Recent advances on critical nodes of embryo engineering technology	**Publisher imprint**	N/A
		**Expected publication date**	2021-05-15
**Author**	Mingwei Gu	**Expected size (number of pages)**	68

**Publication**	Theranostics	**Standard identifier**	N/A
**Publisher**	Ivyspring International Publisher		

ADDITIONAL DETAILS

**Order reference number**	N/A	**The requesting person / organization to appear on the license**	Mingwei Gu

REUSE CONTENT DETAILS

**Title, description or numeric reference of the portion(s)**	figure 1,figure 5	**Title of the article/chapter the portion is from**	Integration of single oocyte trapping, in vitrofertilization and embryo culture in a microwell-structured microfluidic device
**Editor of portion(s)**	N/A	**Author of portion(s)**	Royal Society of Chemistry (Great Britain)
**Volume of serial or monograph**	2010.10	**Issue, if republishing an article from a serial**	N/A
**Page or page range of portion**	2848-2854	**Publication date of portion**	2010-09-15

Total Items: 1 **Total Due: 0.00 USD**

Accepted: All Publisher and CCC Terms and Conditions
