# Supplementary material for: Recent advances in critical nodes of embryo engineering technology
Source: Theranostics. 2021 May 25;11(15):7391–424. doi: 10.7150/thno.58799 (PMC8210615; doi:10.7150/thno.58799)
Supplement: Supplementary file 1 — Supplementary figures and tables. [file thnov11p7391s1.zip › Supplementary material/Figures.docx]

#
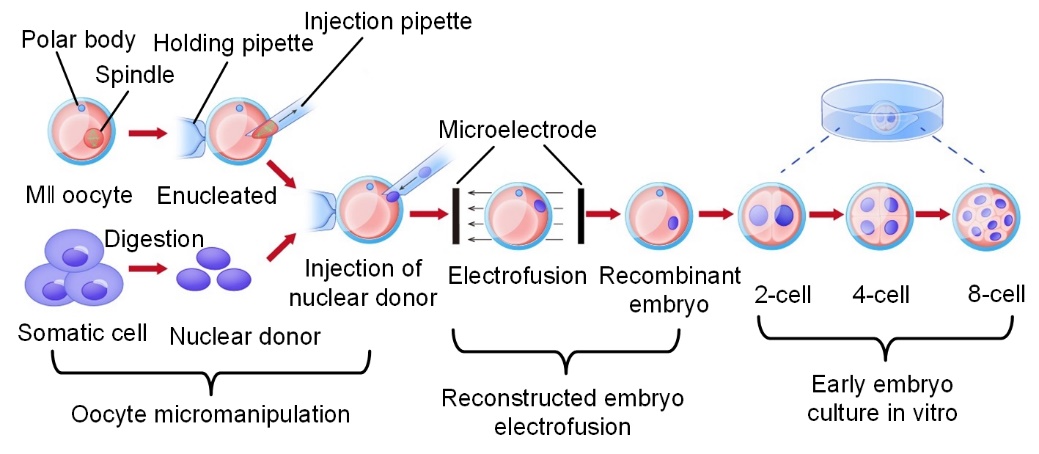


**Figure 1.** **Critical nodes in the process of embryo engineering technology** (take SCNT as an example).

#
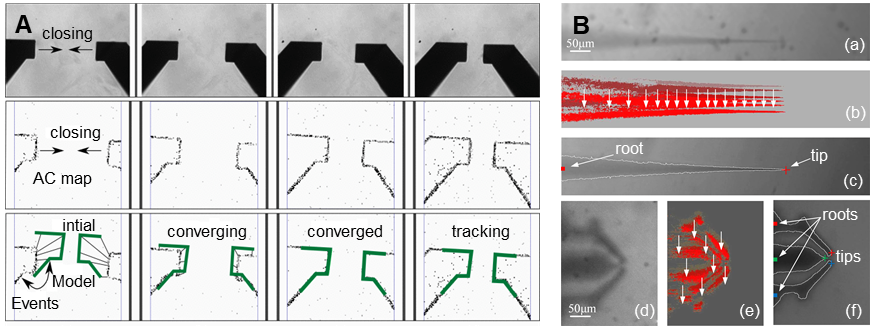


**Figure 2. Image recognition of the end-effector.** (A) Track the position of the microgripper through an iterative closest point algorithm. Adapted with permission from [18]. (B) Recognize microinjection needles and microtweezers through algorithms based on motion history images (MHI) and active contour models. Adapted with permission from [18], copyright 2012 IEEE, and [22], copyright 2013 IEEE.


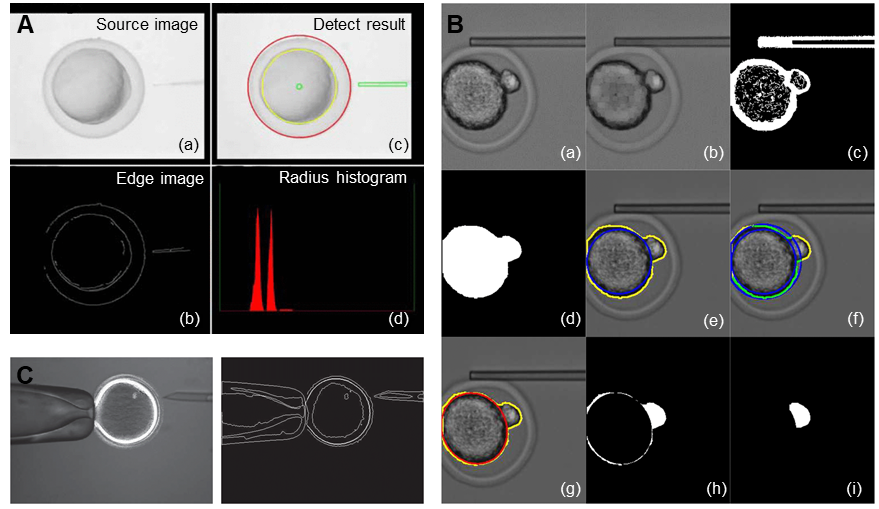


**Figure 3. Image recognition of different parts of oocytes.** (A) Detection of the chorion and cytoplasm of zebrafish oocytes and the edge of the microinjection needle by the Canny algorithm. (B) Detection of the existence and direction of polar bodies through image processing. (C) The fusion images from polarized light microscopy imaging systems and the traditional optical inverted microscope imaging system and the edge information of the fusion image. Adapted with permission from [34], copyright 2009 IEEE, and [39], copyright 2017 IEEE, and [48], copyright 2013 IEEE.

#
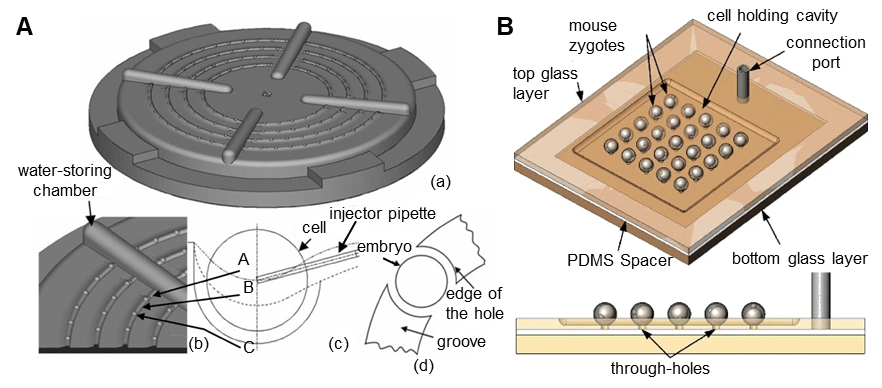


**Figure 4. Contact cell fixation device.** (A) The zebrafish embryo fixation device with arrayed hemispherical grooves. (B) The glass chip with arrayed round holes. Adapted with permission from [34], copyright 2009 IEEE, and [53], copyright 2009 Springer Nature.

#
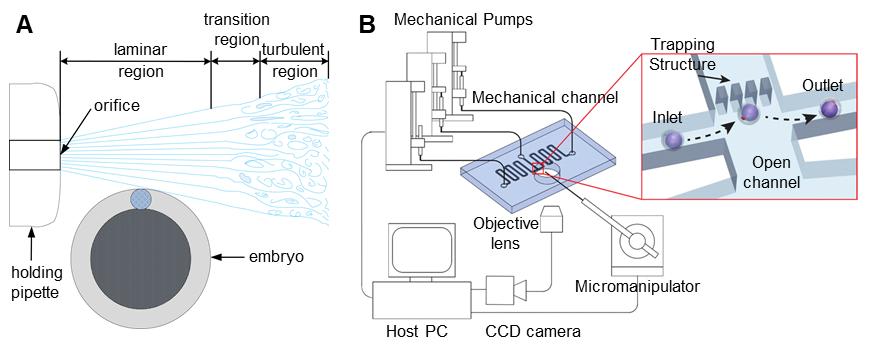


**Figure 5.** (A) Sun uses the torque generated by the fluid flow to rotate the cells. (B) A microfluidic platform used to achieve oocyte capture and direction control. Adapted with permission from [35], copyright 2012 IEEE, and [58], copyright 2013 IEEE.

#
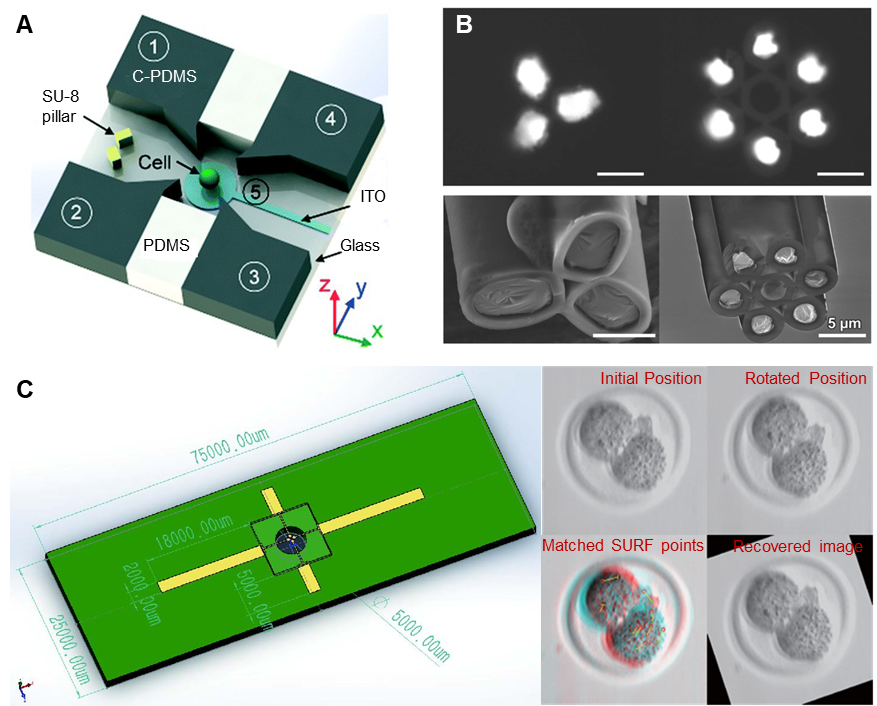


# Figure 6. (A) Schematic diagram of a dielectrophoretic chip. (B) Scanning electron microscopy image of liquid metal-based capillary microneedle electrodes. (C) The effect of dielectrophoresis forceps and drive oocyte rotation. Adapted with permission from [62], copyright 2018 Royal Society of Chemistry, and [63], copyright 2018 John Wiley and Sons, and [64], copyright 2020 IEEE.

#
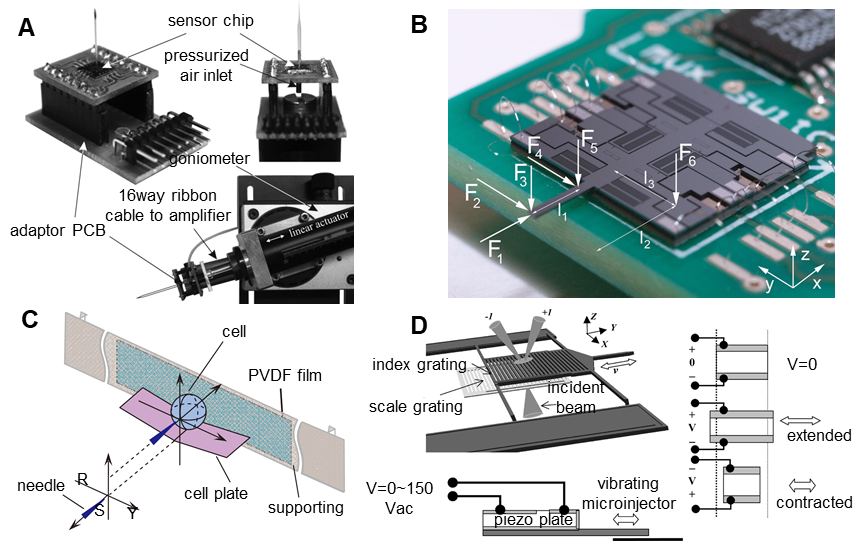


# Figure 7. Various sensors for measuring the injection force. (A) Piezoresistive force sensor. (B) Capacitive six-axis force sensor based on the MEMS process. (C) Microforce sensor based on piezoelectric material PVDF. (D) A micro-optical force sensor. Adapted with permission from [52], copyright 2012 IEEE, and [81], copyright 2009 IEEE, and [84], copyright 2009 SAGE Publications, and [87], copyright 2006 IEEE.

#
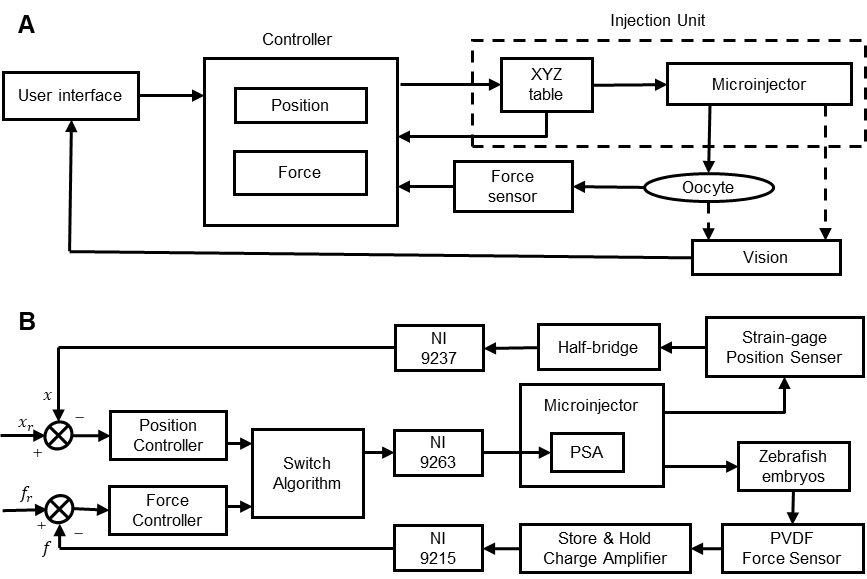


# Figure 8. (A) Schematic diagram of the cell injection system with inner loop impedance control and outer loop feedback control. (B) An injection system solution with position/force switch control. Adapted with permission from [84], copyright 2009 SAGE Publications, and [97], copyright 2017 IEEE.

#
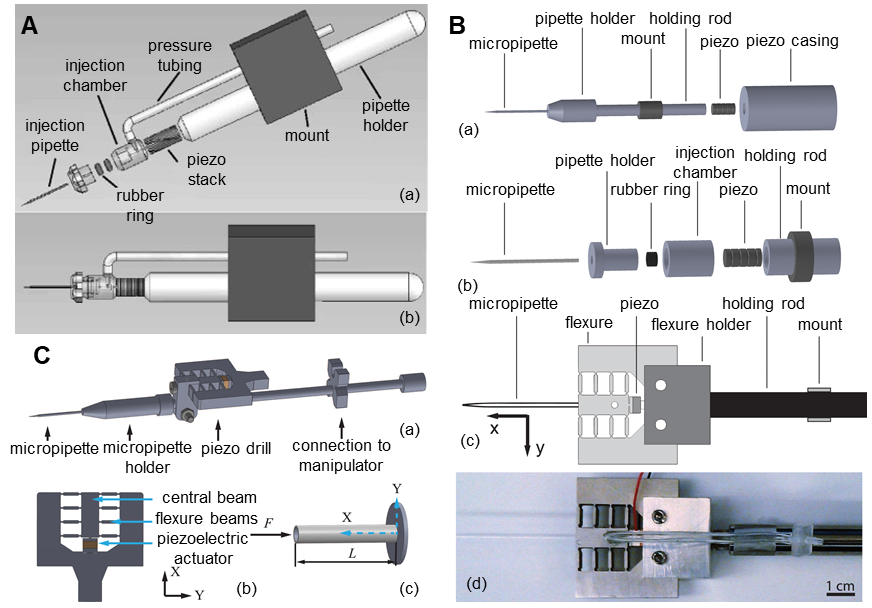


# Figure 9. Various improved piezoelectric ultrasonic microinjectors. (A) Microinjector with piezoelectric ceramic front. (B) Piezoelectric ultrasonic microinjector with a flexible mechanism. (C) Microinjector with piezoelectric actuator eccentric configuration. Adapted with permission from [104], copyright 2011 Spring Nature, and [106], copyright 2018 IEEE, and [107], copyright 2020 IEEE.

#
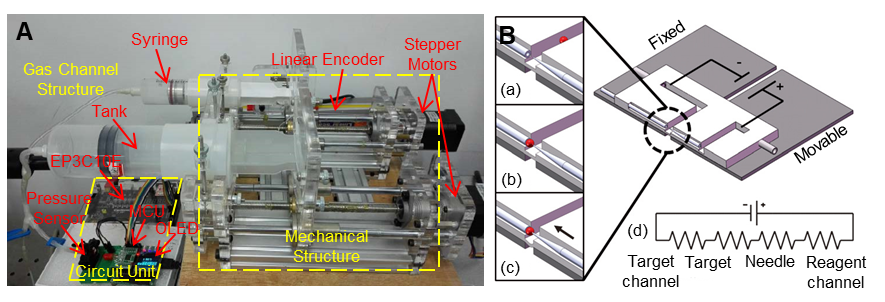


# Figure 10. (A) High-precision pressure-driven pump for the quantitative injection of picolitre. (B) Electrical schematic diagram of the injection system based on electroosmosis. Adapted with permission from [111], copyright 2017 World Scientific Publishing Co, and [112], copyright 2009 Royal Society of Chemistry.

#
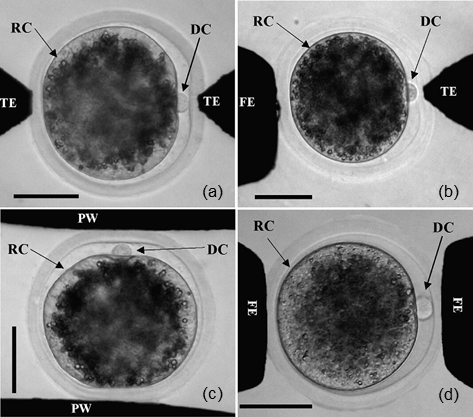


# Figure 11. Schematic diagram of four electrofusion schemes. (a) tip-end plus tip-end (TT); (b) tip-end plus frustum-end (TF); (c) frustum-end plus frustum-end (FF); (d) parallel microelectrodes (PM). Adapted with permission from [156], copyright 2007 Elsevier.

#
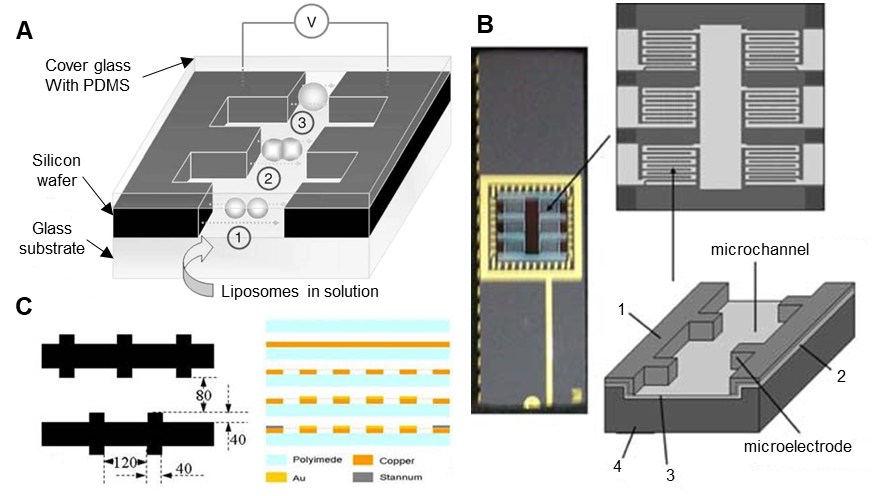


# Figure 12. Various electrofusion chips with interdigitated electrodes. (A) A schematic diagram of a microfluidic chip with a high-aspect-ratio microelectrode array. (B) An electrofusion chip with 1368 pairs of silicon microelectrodes. (C) A schematic diagram of the flexible electrofusion chip. unit: $\boldsymbol{\mu m}$. Adapted with permission from [157], copyright 2004 Spring Nature, and [158], copyright 2008 Springer Nature, and [159], copyright 2009 Elsevier.

#
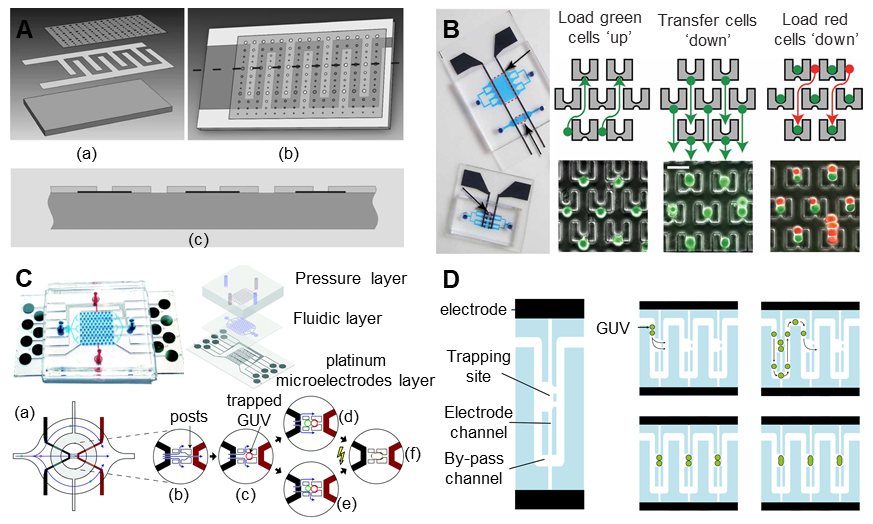


# Figure 13. (A) A double-layer microfluidic chip integrating automatic cell positioning and cell electrofusion. (B) A microfluidic chip integrates thousands of microtraps made of PDMS and its working steps. (C) A microfluidic chip can separate cells of different diameters and perform electrofusion and its working schematic diagram. (D) A microfluidic chip for electrofusion of GUVs and its working diagram. Adapted with permission from [160], copyright 2010 Spring Nature, and [161], copyright 2009 Spring Nature, and [162], copyright 2014 Royal Society of Chemistry, and [163], copyright 2020 Elsevier.


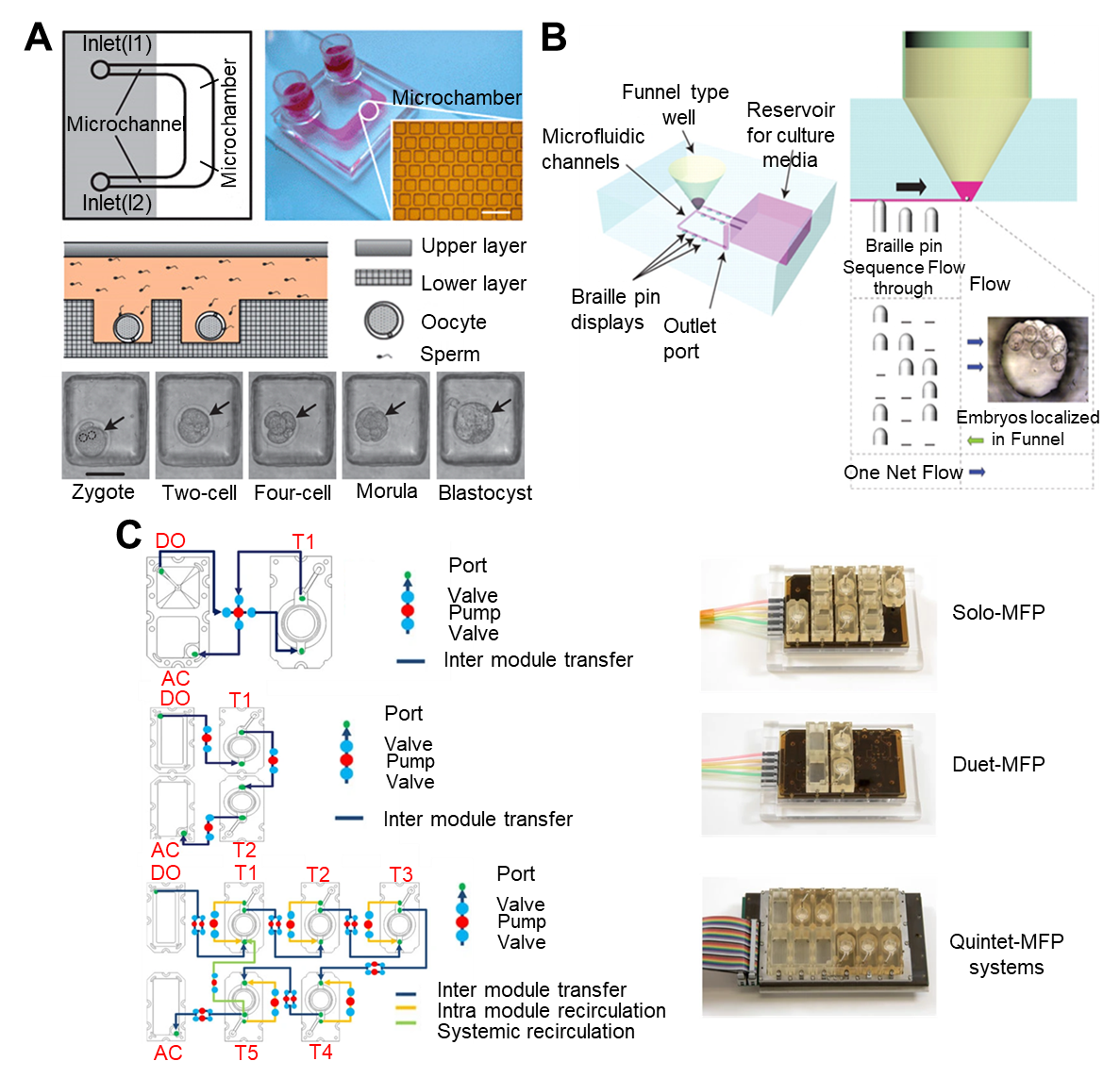


**Figure 14. The mechanical stimulation system** (A) A novel microwell-structured microfluidic device that integrates single oocyte trapping, fertilization and subsequent embryo culture. (B) A dynamic microfunnel embryo culture system would enhance outcomes by better mimicking the fluid mechanical and biochemical stimulation embryos experience in vivo from ciliary currents and oviductal contractions. (C) A microfluidic system that supports murine ovarian follicles to produce the human 28-day menstrual cycle hormone profile, which controls the human female reproductive tract and peripheral tissue dynamics in single-, dual- and multiple-unit microfluidic platforms. Adapted with permission from [217], copyright 2011 Royal Society of Chemistry, and [222], copyright 2018 Oxford University Press, and [223], copyright 2020 Nature Pub. Group.


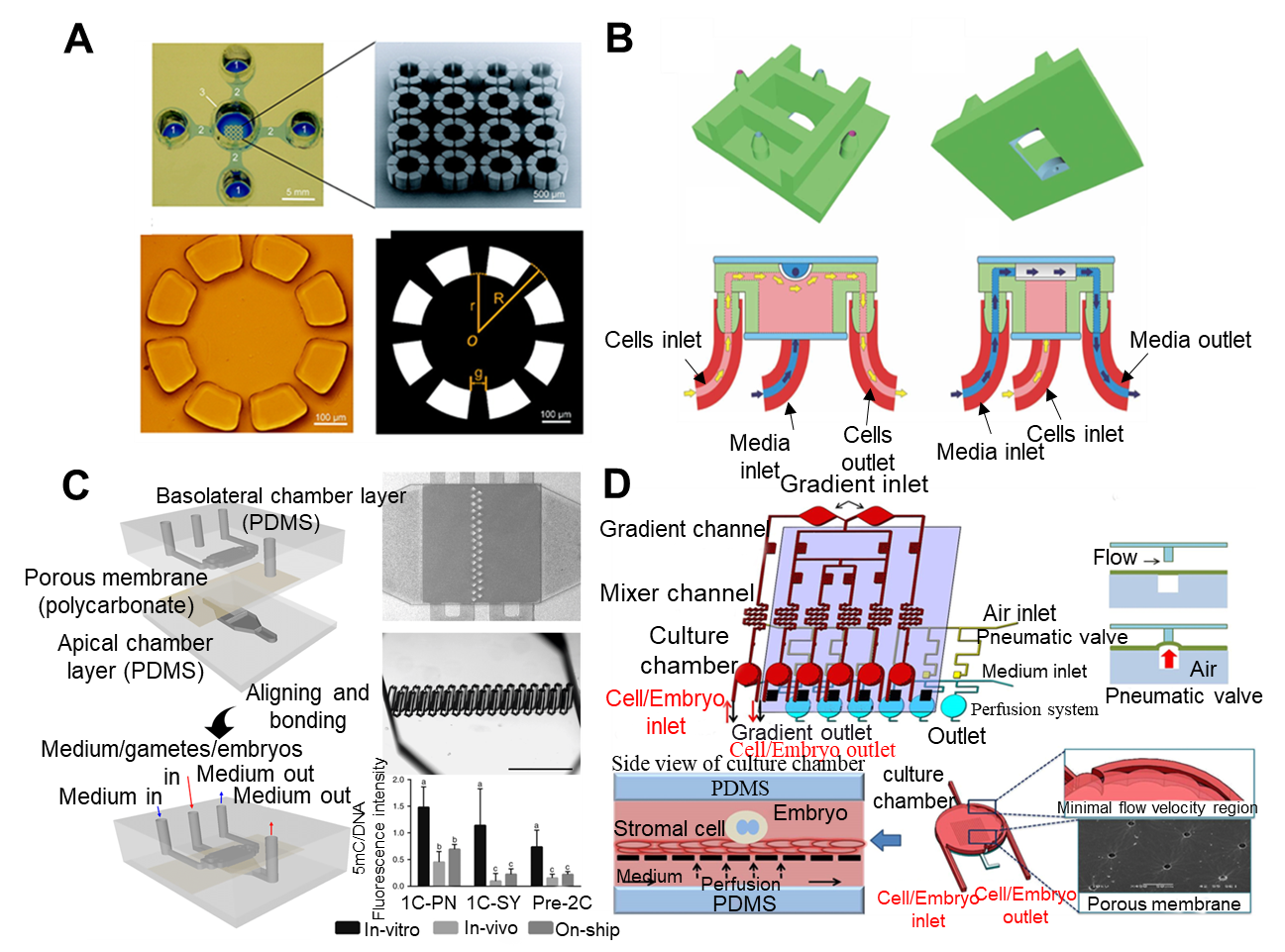


**Figure 15. Uterine-like co-culture microfluidic chip** (A) A novel microdevice that integrates each step of IVF, including oocyte positioning, sperm screening, fertilization, medium replacement, and embryo culture. (B) A U-shaped porous membrane three-dimensional fallopian tube model chip. (C) An oviduct-on-a-chip platform to better investigate the mechanisms related to (epi)genetic reprogramming and the degree to which they differ between in vitro and in vivo embryos. (D) A microfluidic chip co-cultured with embryos and endometrial stromal cells. Adapted with permission from [226], copyright 2011 American Chemical Society, and [227], copyright 2017 Royal Society of Chemistry, and [228], copyright 2018 Nature Pub. Group, and [229], copyright 2016 Elsevier Sequoia.


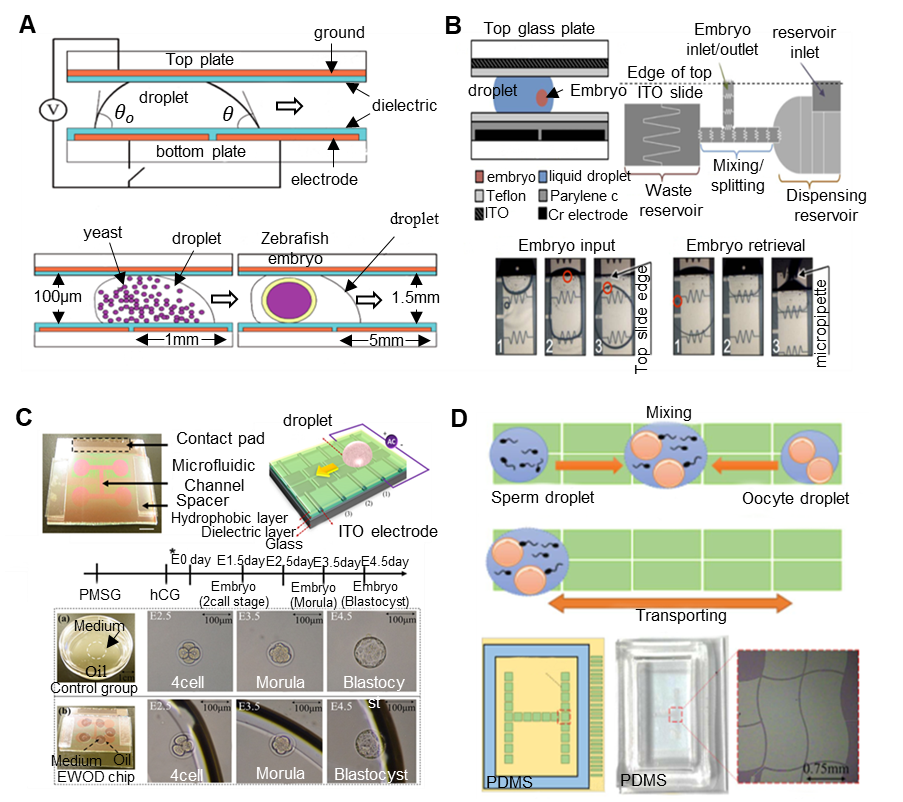


**Figure 16. DMF-style dynamic cultivation system.** (A) Dual-plate digital microfluidic device EWOD technology. (B) Digital microfluidic equipment to automate the processing of vitrified frozen embryos. (C) A microfluidic microchannel system for IVF is considered to provide an improved in vivo-mimicking environment to enhance the development of an embryo culture system before implantation. (D) A new DMF chip design with a PDMS ring has solved the problem of gas exchange during long-term embryo culture. Adapted with permission from [247], copyright 2009 Royal Society of Chemistry, and [248], copyright 2014 Public Library of Science, and [249], copyright 2015 Public Library of Science, and [250], copyright 2015 IEEE.

#
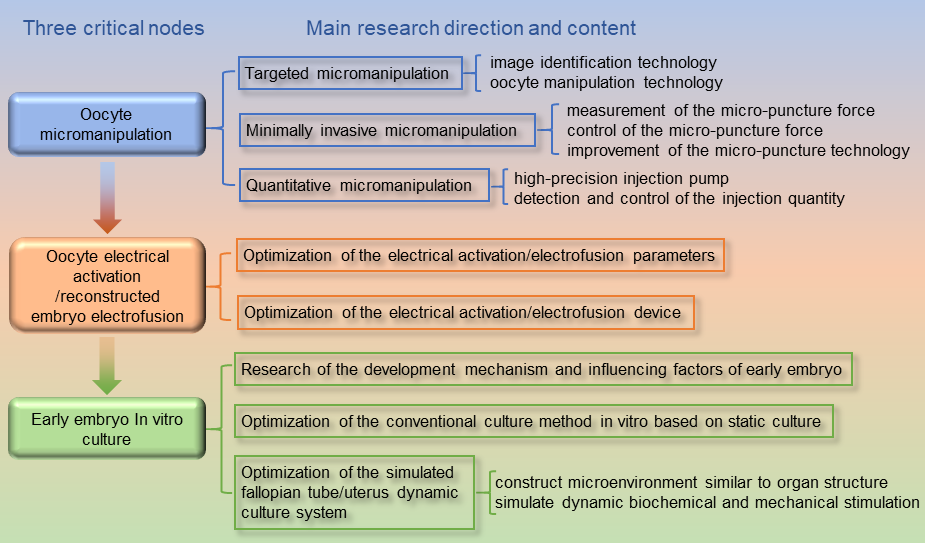


# Figure 17. Critical nodes in embryo engineering technology and their main research contents
