# Supplementary material for: Recent advances in critical nodes of embryo engineering technology
Source: Theranostics. 2021 May 25;11(15):7391–424. doi: 10.7150/thno.58799 (PMC8210615; doi:10.7150/thno.58799)
Supplement: Supplementary file 1 — Supplementary figures and tables. [file thnov11p7391s1.zip › Supplementary material/Introduction of Graphical Abstract.docx]

By studying the existing embryo engineering technology process, we discovered three critical nodes that have the greatest impact on the development of oocytes and early embryos, namely, oocyte micromanipulation, oocyte electrical activation/reconstructed embryo electrofusion, and the in vitro culture of early embryos. This article mainly demonstrates the efforts made by researchers in the relevant technologies of these three critical nodes from an engineering perspective, analyses the shortcomings of the current technology, and proposes a plan and prospects for the development of embryo engineering technology in the future.
