# Supplementary figures and images for: Recent advances in critical nodes of embryo engineering technology
Source: Theranostics. 2021 May 25;11(15):7391–424. doi: 10.7150/thno.58799 (PMC8210615; doi:10.7150/thno.58799)

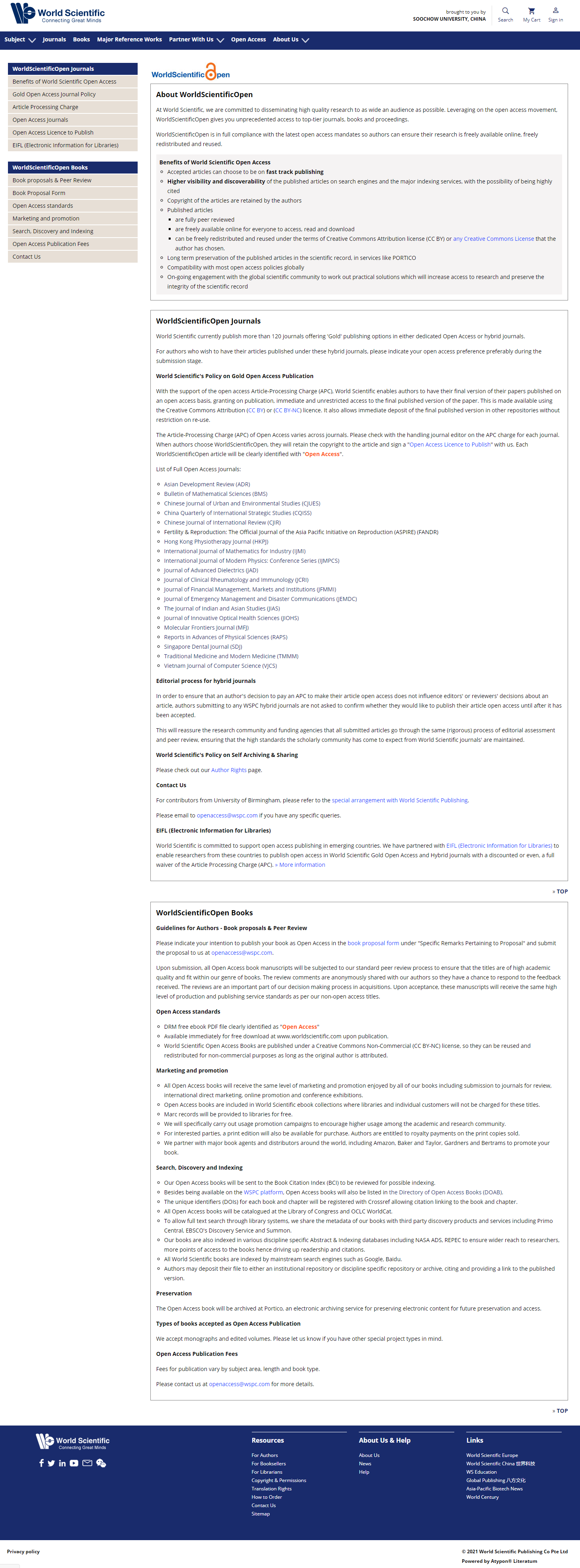

Supplement: Supplementary file 1 — Supplementary figures and tables. [file thnov11p7391s1.zip › Supplementary material/Figure copyright/10A.png]

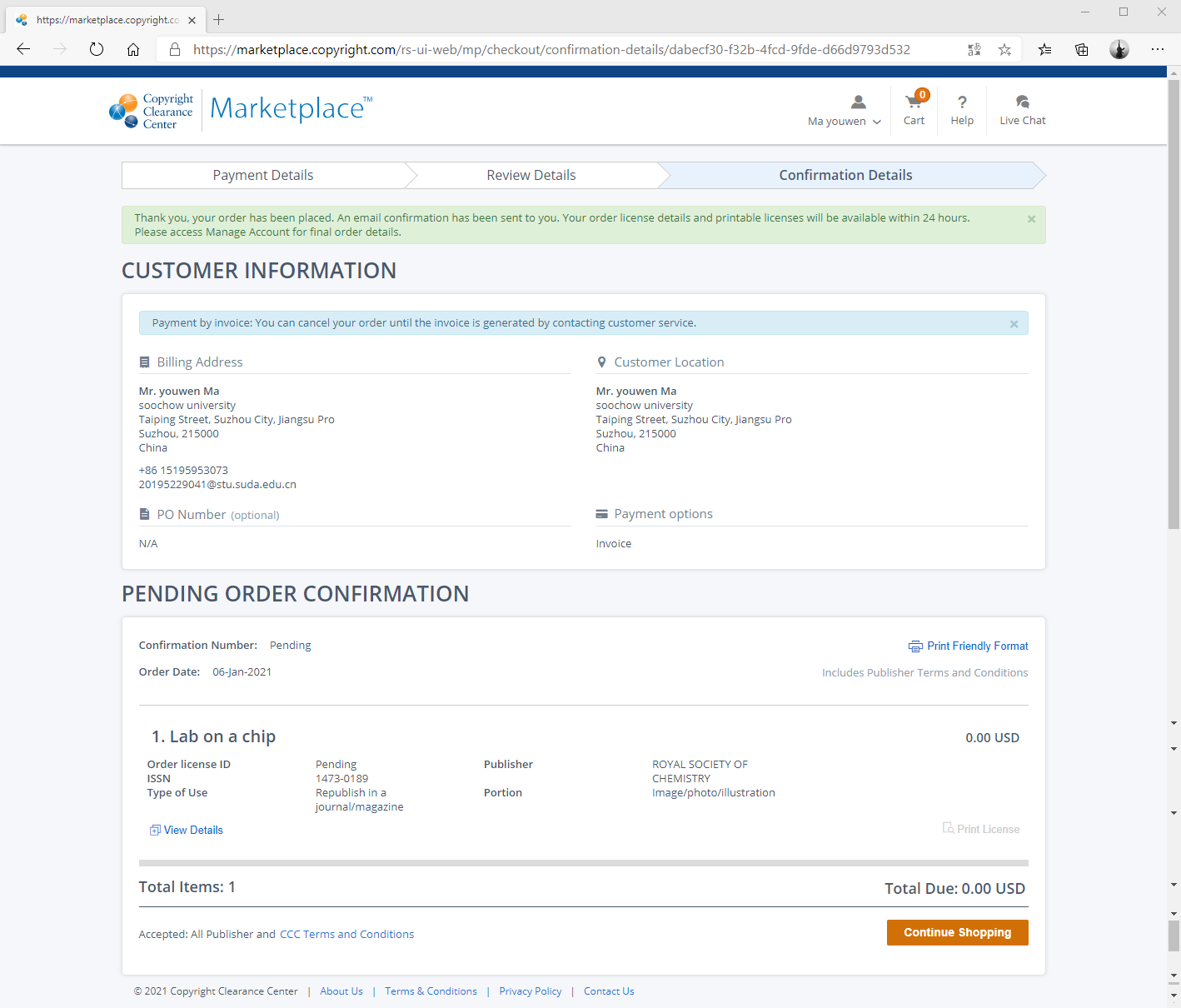

Supplement: Supplementary file 1 — Supplementary figures and tables. [file thnov11p7391s1.zip › Supplementary material/Figure copyright/10B.png]

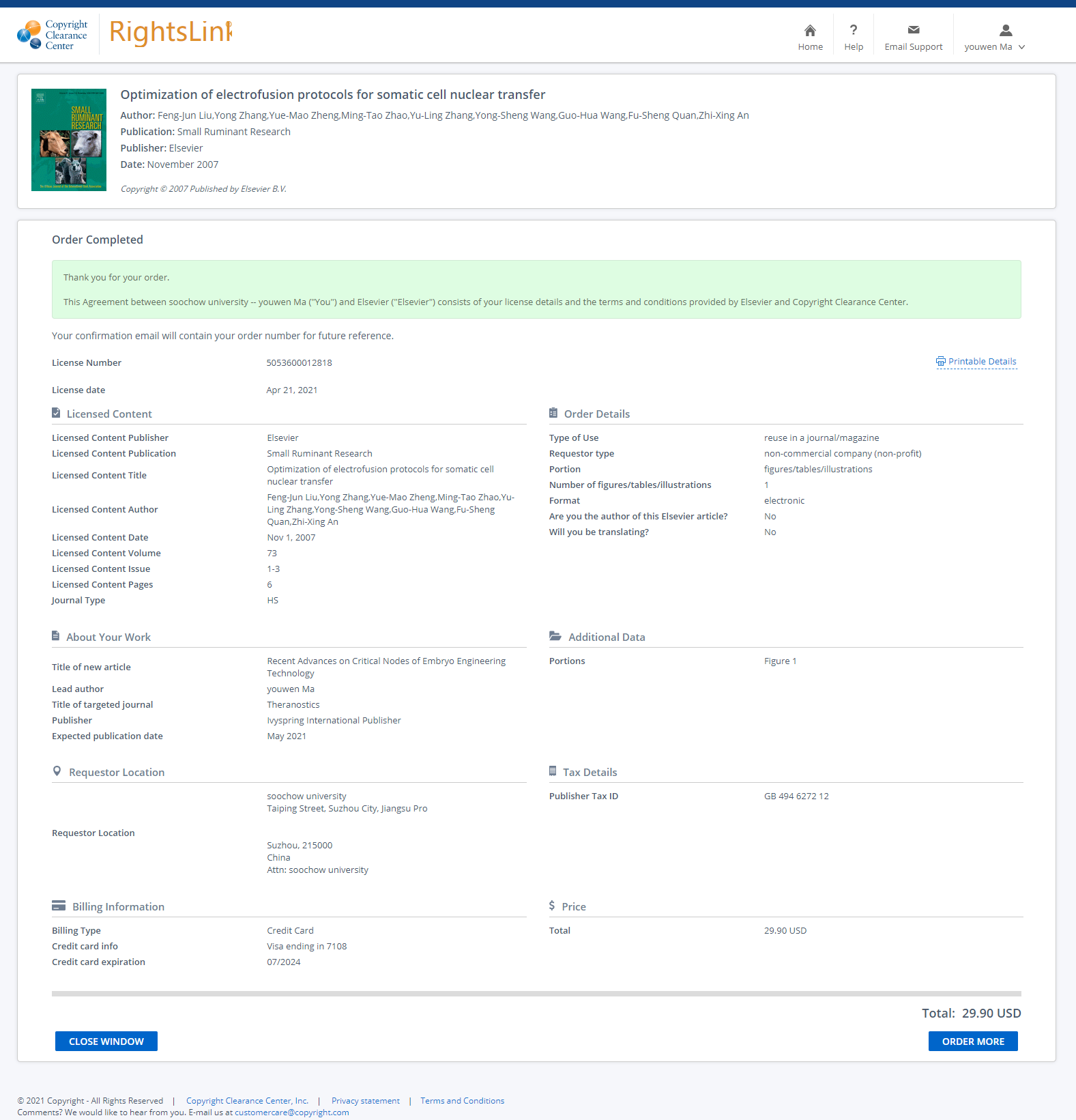

Supplement: Supplementary file 1 — Supplementary figures and tables. [file thnov11p7391s1.zip › Supplementary material/Figure copyright/11.png]

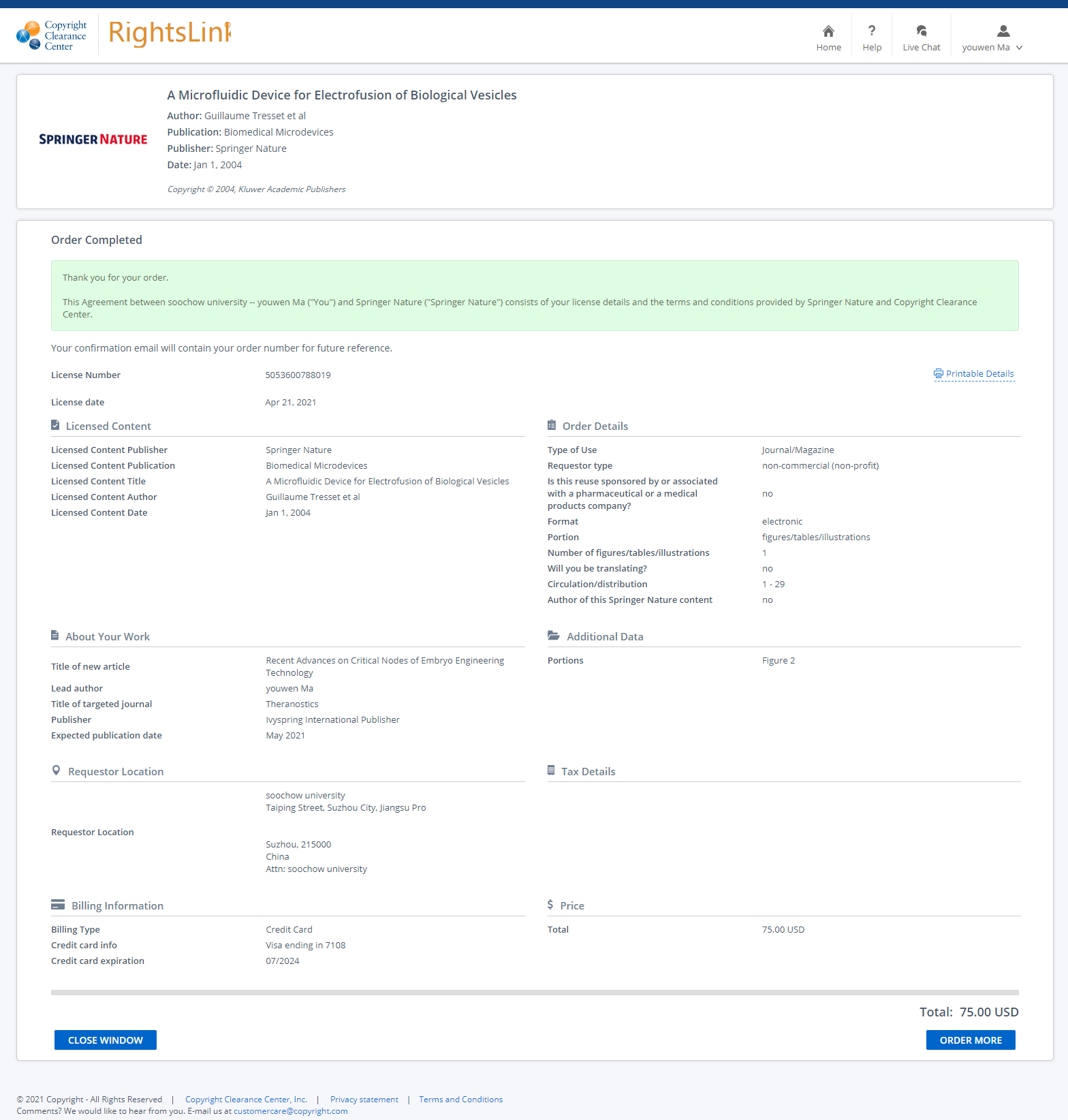

Supplement: Supplementary file 1 — Supplementary figures and tables. [file thnov11p7391s1.zip › Supplementary material/Figure copyright/12A.png]

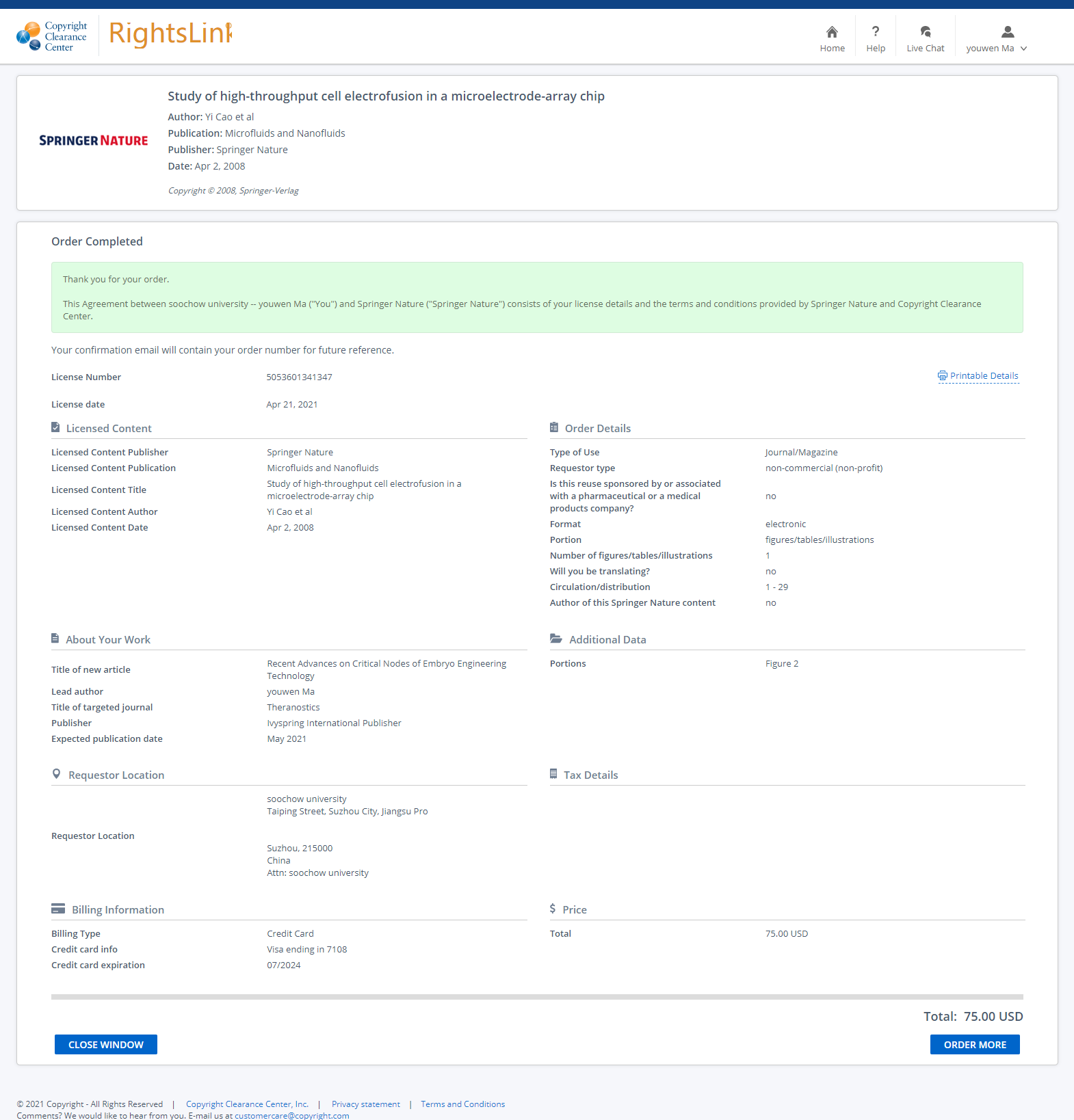

Supplement: Supplementary file 1 — Supplementary figures and tables. [file thnov11p7391s1.zip › Supplementary material/Figure copyright/12B.png]

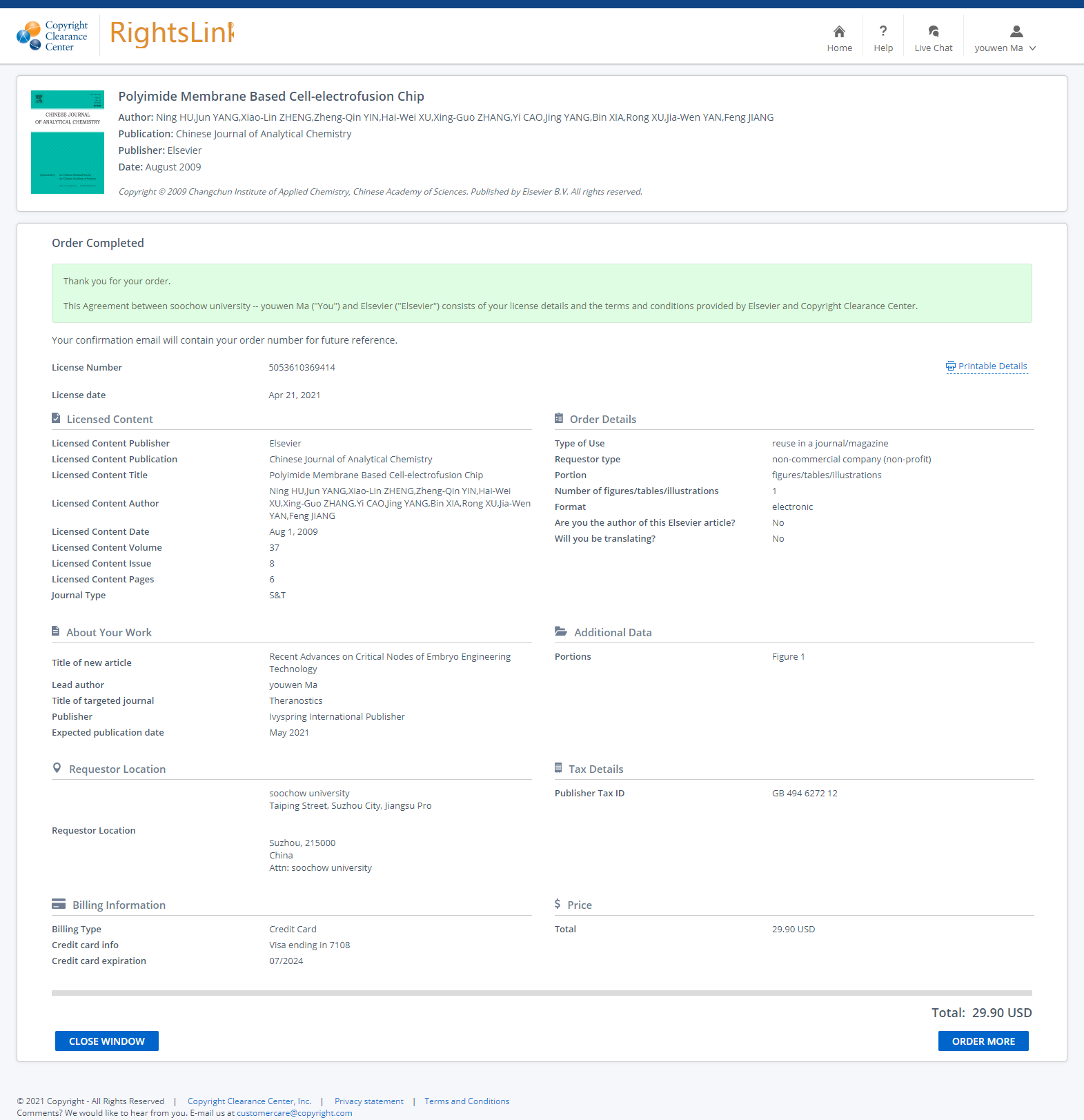

Supplement: Supplementary file 1 — Supplementary figures and tables. [file thnov11p7391s1.zip › Supplementary material/Figure copyright/12C.png]

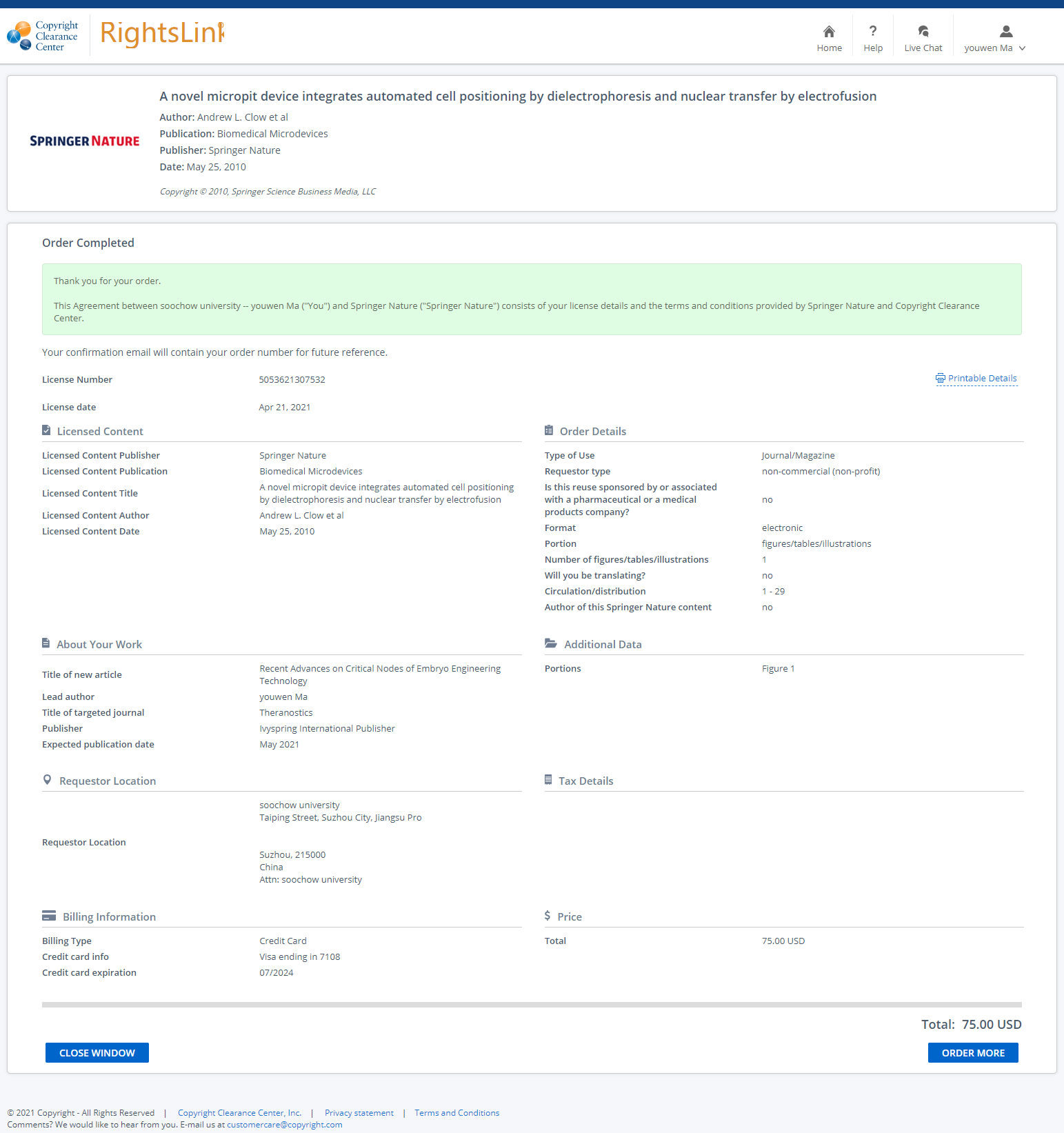

Supplement: Supplementary file 1 — Supplementary figures and tables. [file thnov11p7391s1.zip › Supplementary material/Figure copyright/13A.png]

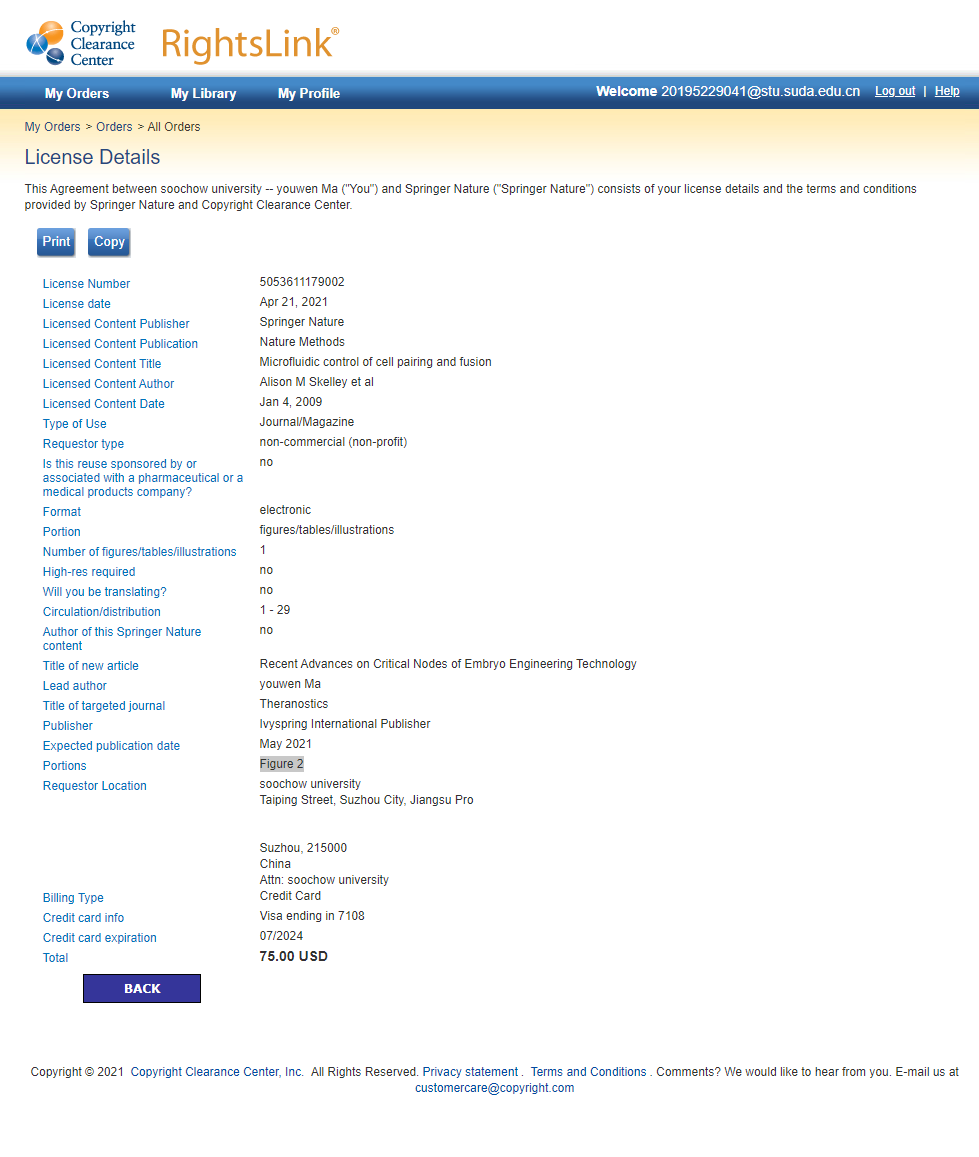

Supplement: Supplementary file 1 — Supplementary figures and tables. [file thnov11p7391s1.zip › Supplementary material/Figure copyright/13B.png]

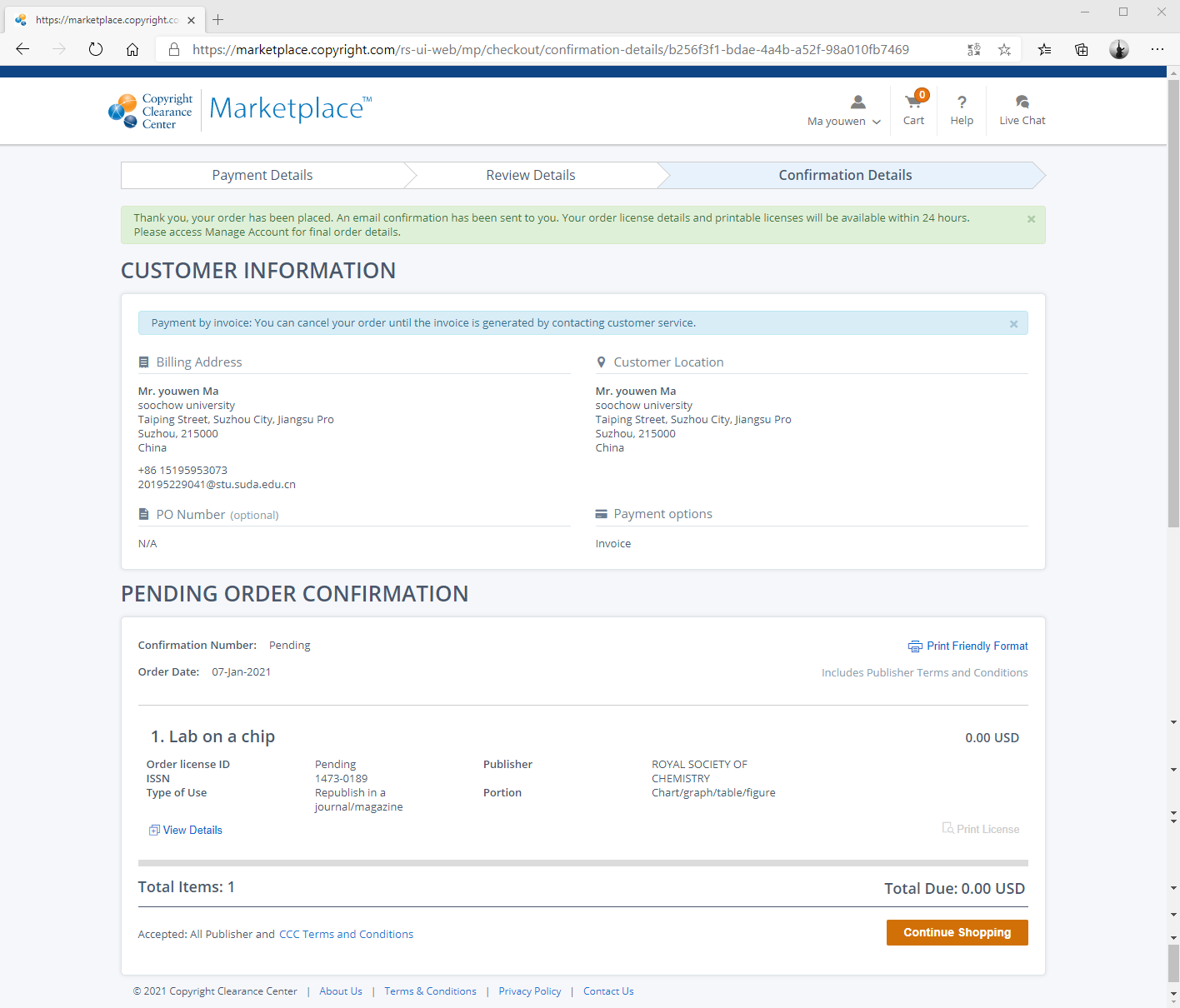

Supplement: Supplementary file 1 — Supplementary figures and tables. [file thnov11p7391s1.zip › Supplementary material/Figure copyright/13C.png]

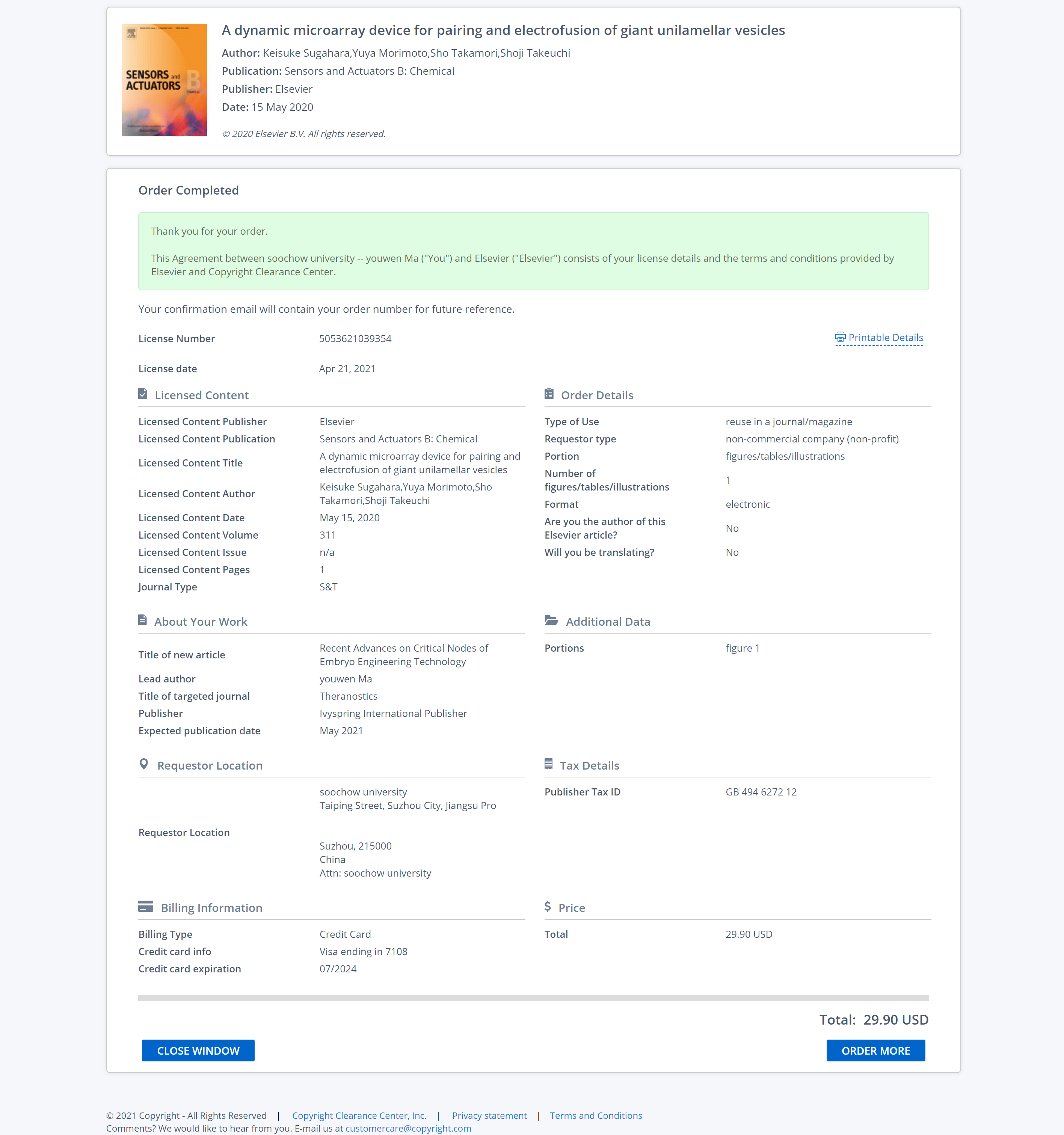

Supplement: Supplementary file 1 — Supplementary figures and tables. [file thnov11p7391s1.zip › Supplementary material/Figure copyright/13D.png]

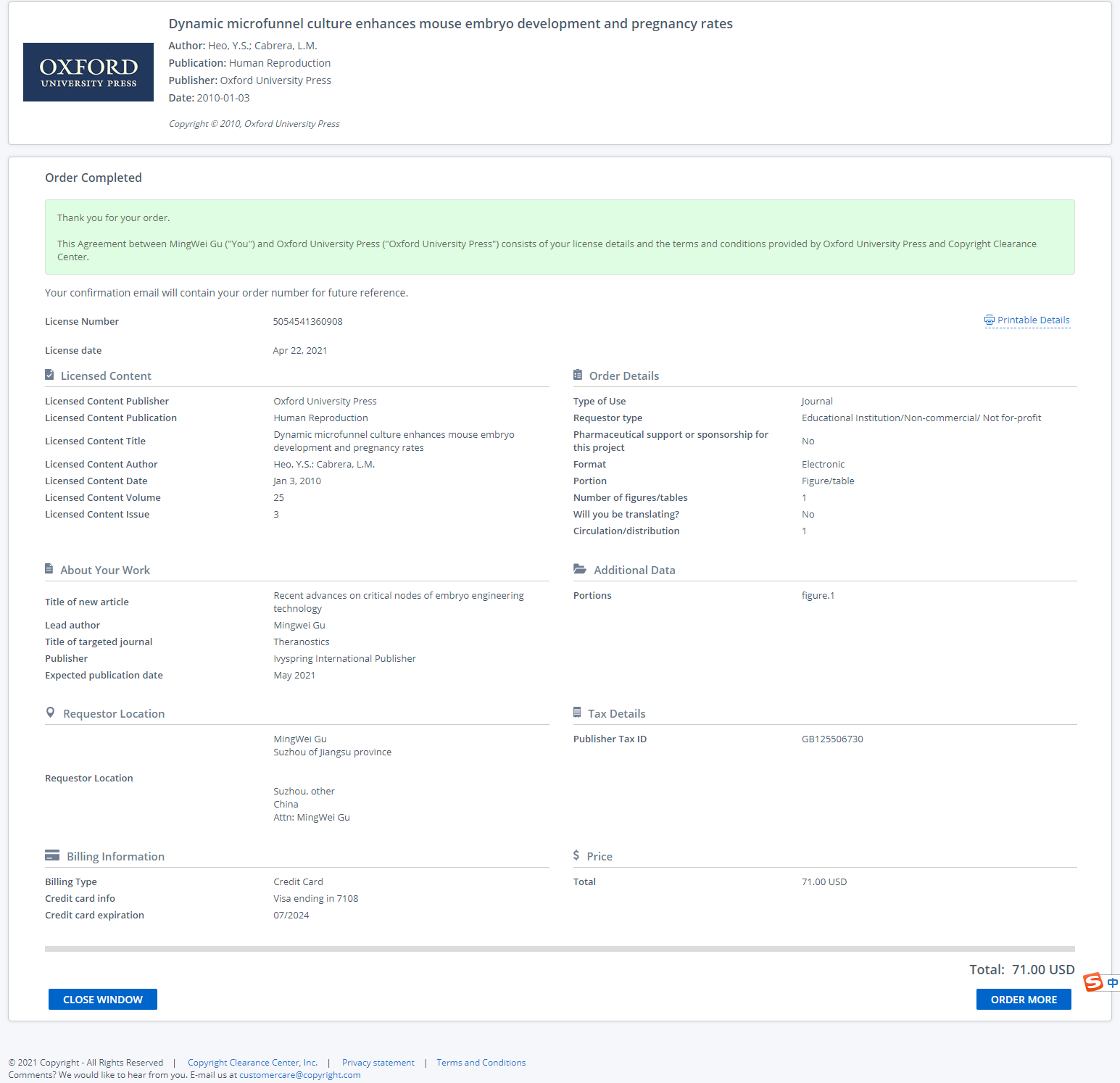

Supplement: Supplementary file 1 — Supplementary figures and tables. [file thnov11p7391s1.zip › Supplementary material/Figure copyright/14b.png]

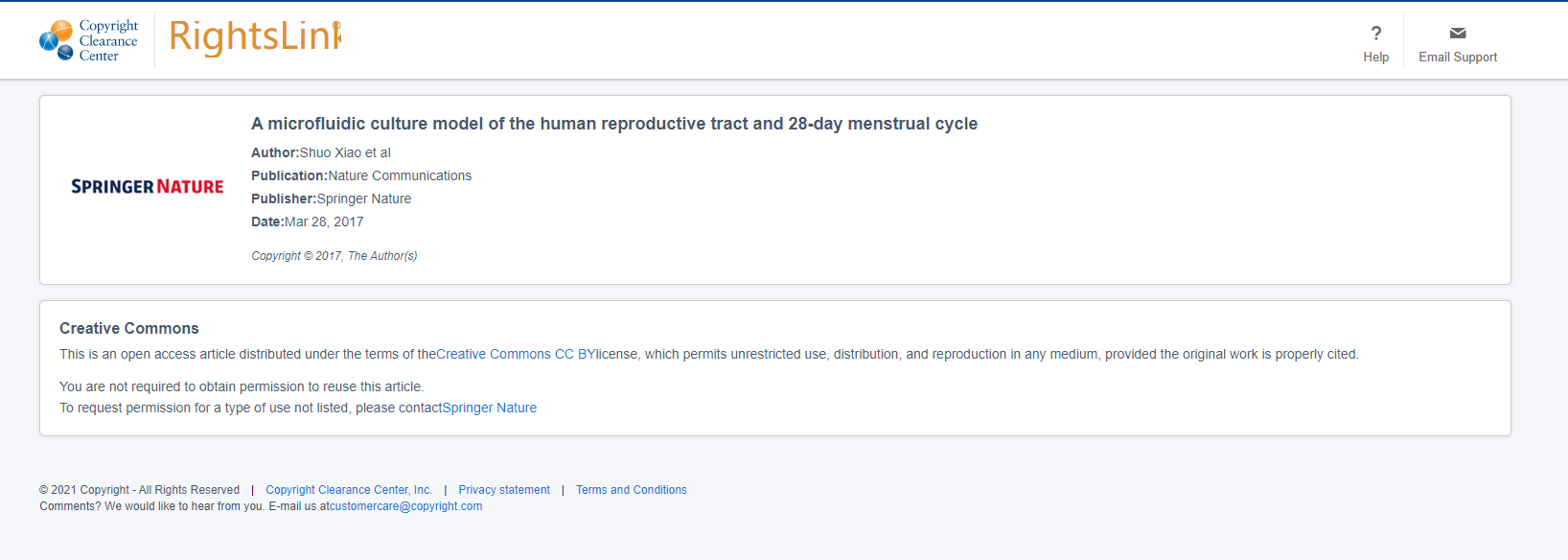

Supplement: Supplementary file 1 — Supplementary figures and tables. [file thnov11p7391s1.zip › Supplementary material/Figure copyright/14c.png]

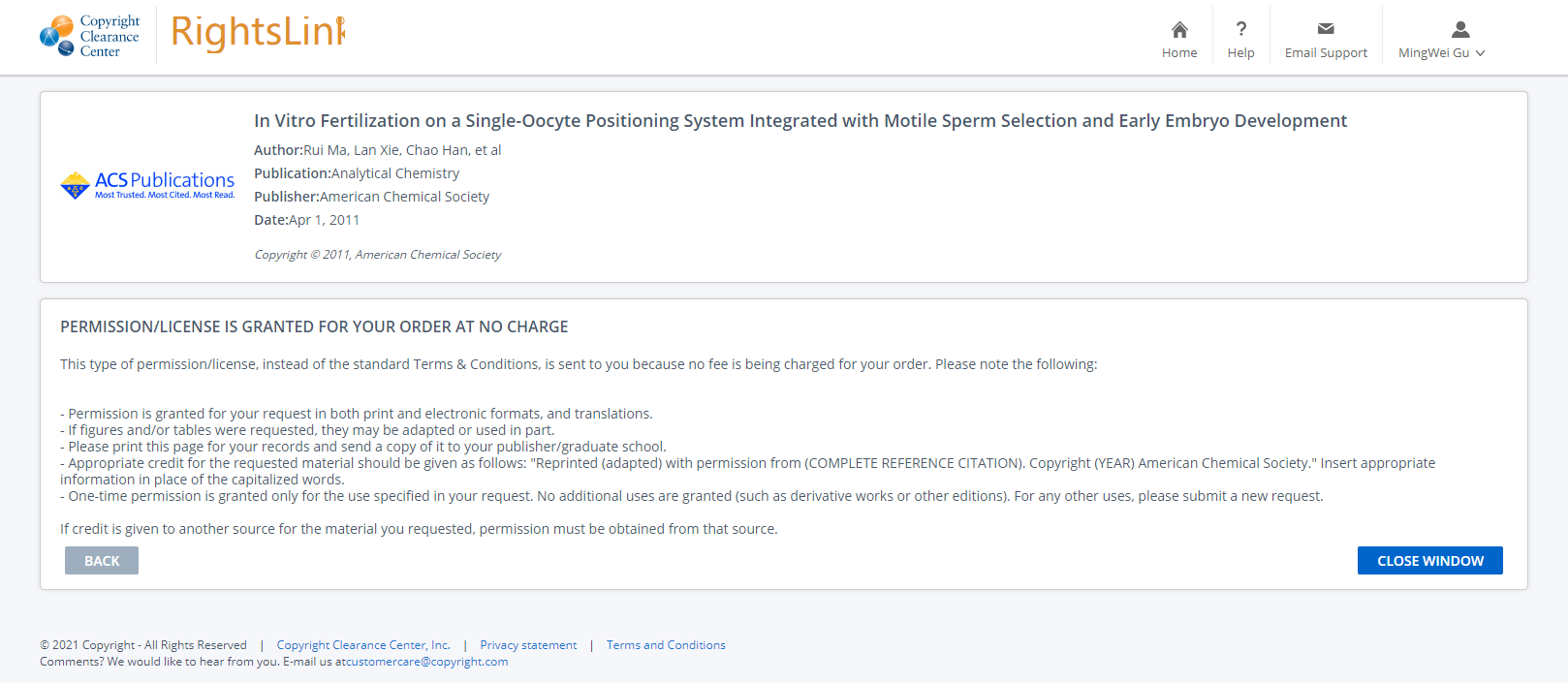

Supplement: Supplementary file 1 — Supplementary figures and tables. [file thnov11p7391s1.zip › Supplementary material/Figure copyright/15a.png]

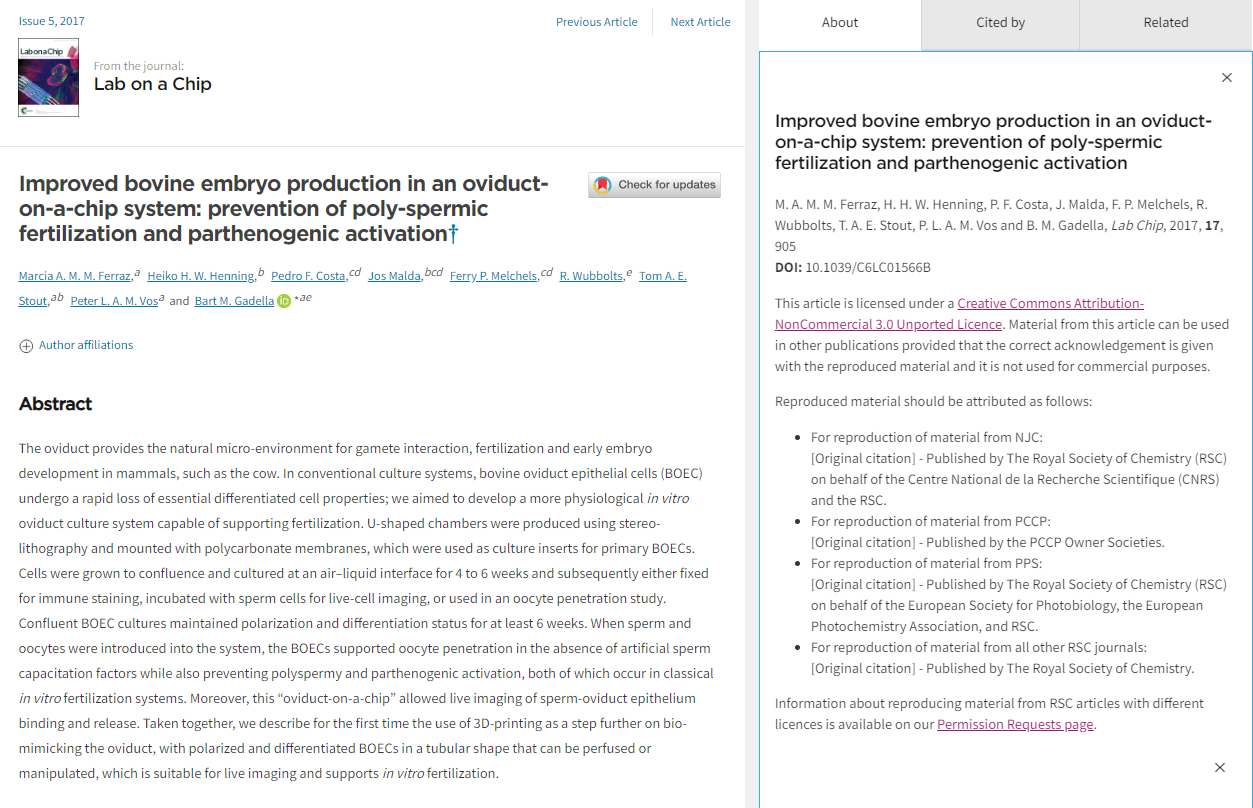

Supplement: Supplementary file 1 — Supplementary figures and tables. [file thnov11p7391s1.zip › Supplementary material/Figure copyright/15b.png]

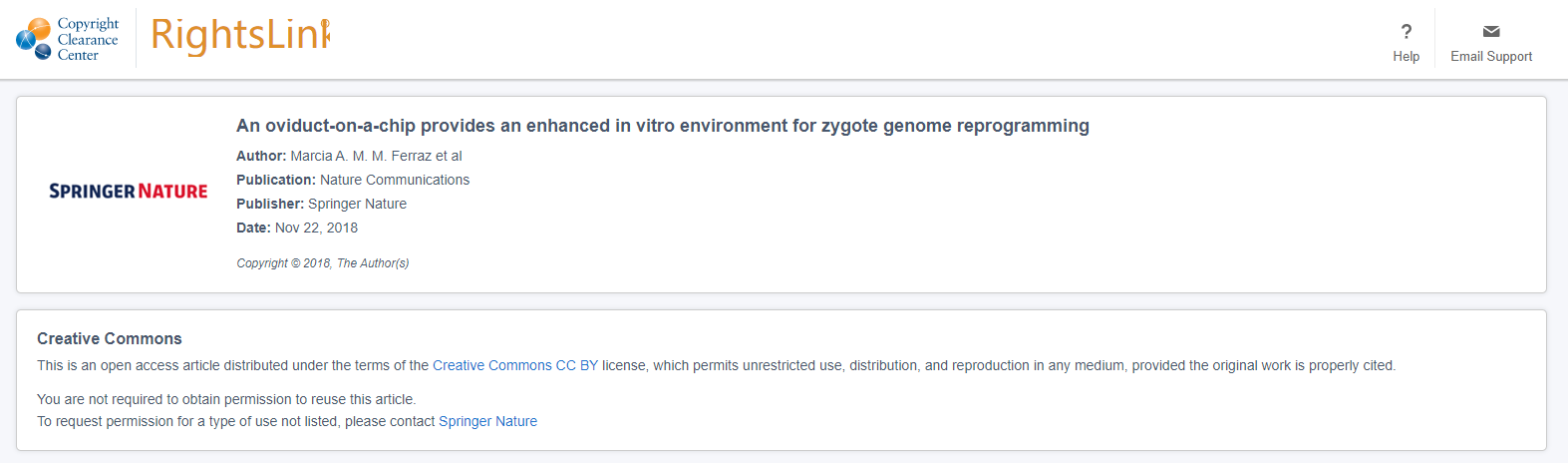

Supplement: Supplementary file 1 — Supplementary figures and tables. [file thnov11p7391s1.zip › Supplementary material/Figure copyright/15c.png]

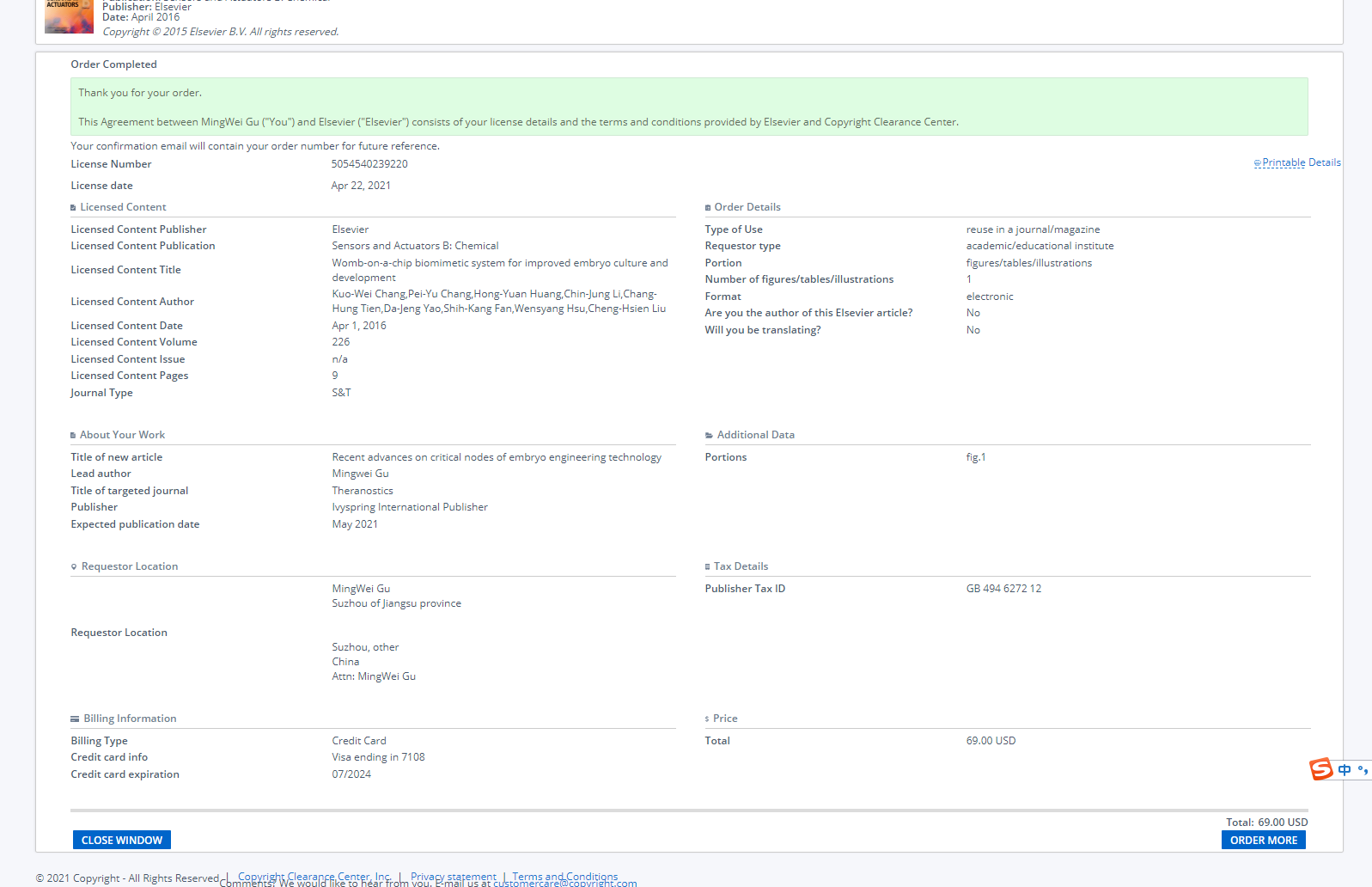

Supplement: Supplementary file 1 — Supplementary figures and tables. [file thnov11p7391s1.zip › Supplementary material/Figure copyright/15d.png]

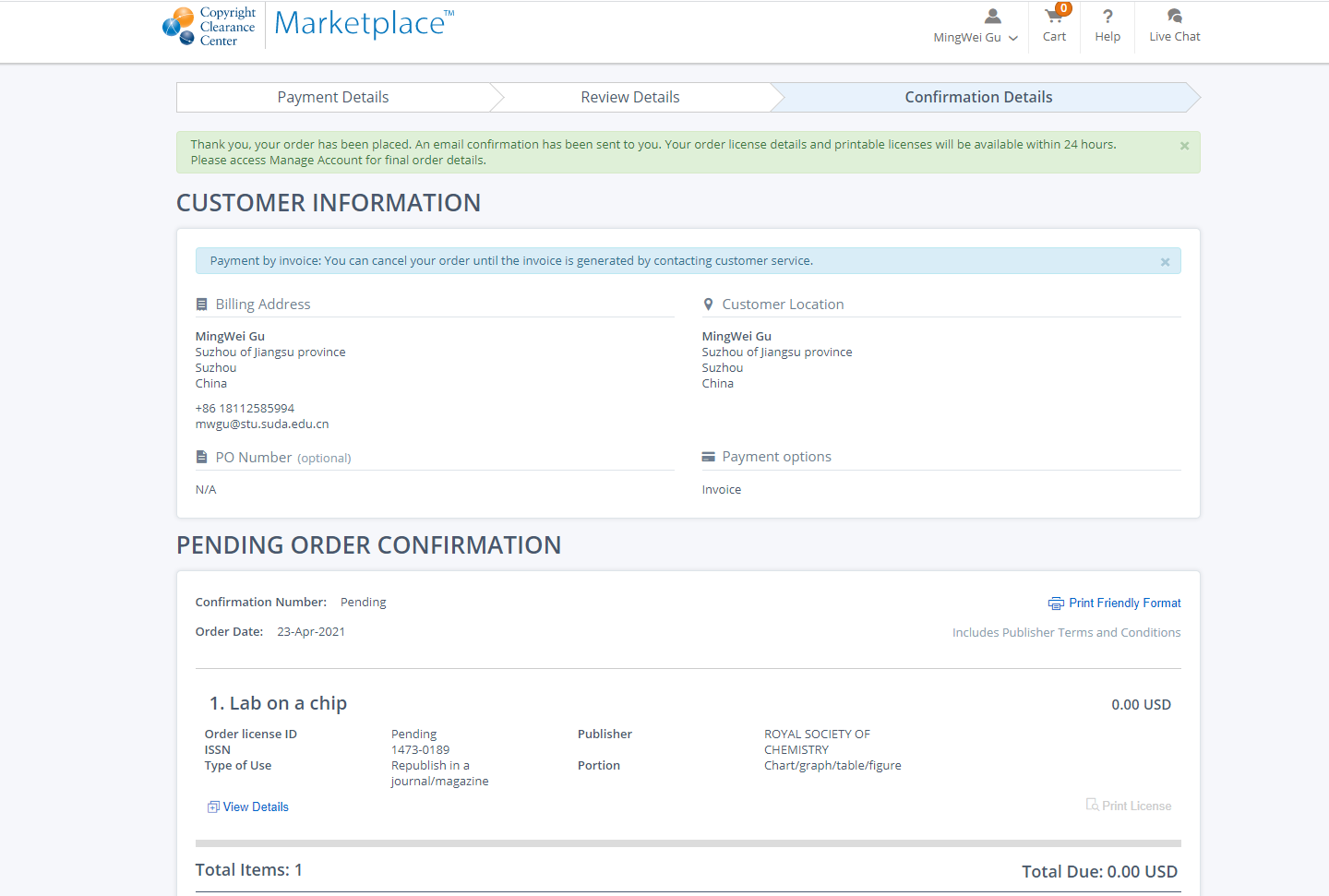

Supplement: Supplementary file 1 — Supplementary figures and tables. [file thnov11p7391s1.zip › Supplementary material/Figure copyright/16a.png]

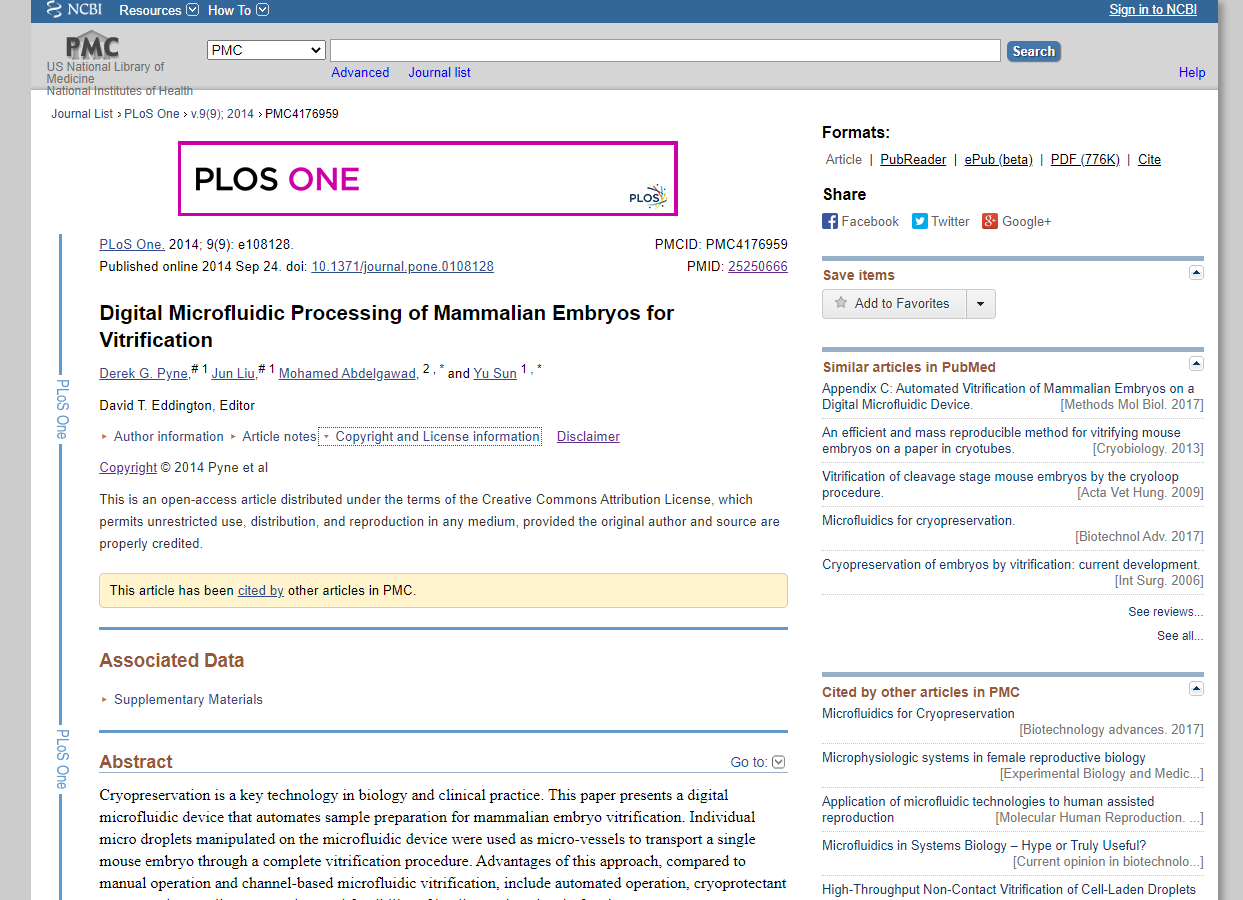

Supplement: Supplementary file 1 — Supplementary figures and tables. [file thnov11p7391s1.zip › Supplementary material/Figure copyright/16b.png]

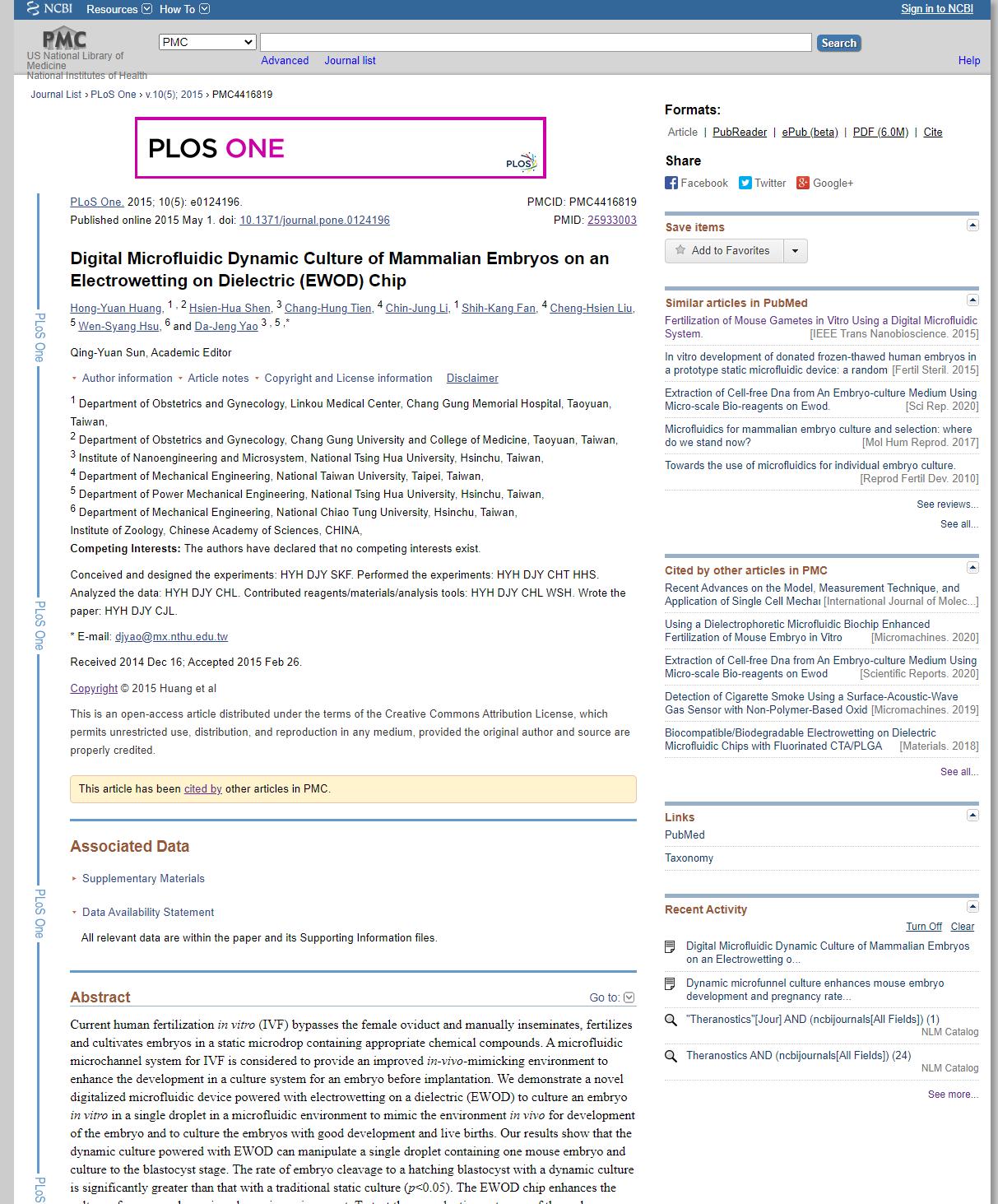

Supplement: Supplementary file 1 — Supplementary figures and tables. [file thnov11p7391s1.zip › Supplementary material/Figure copyright/16c.png]

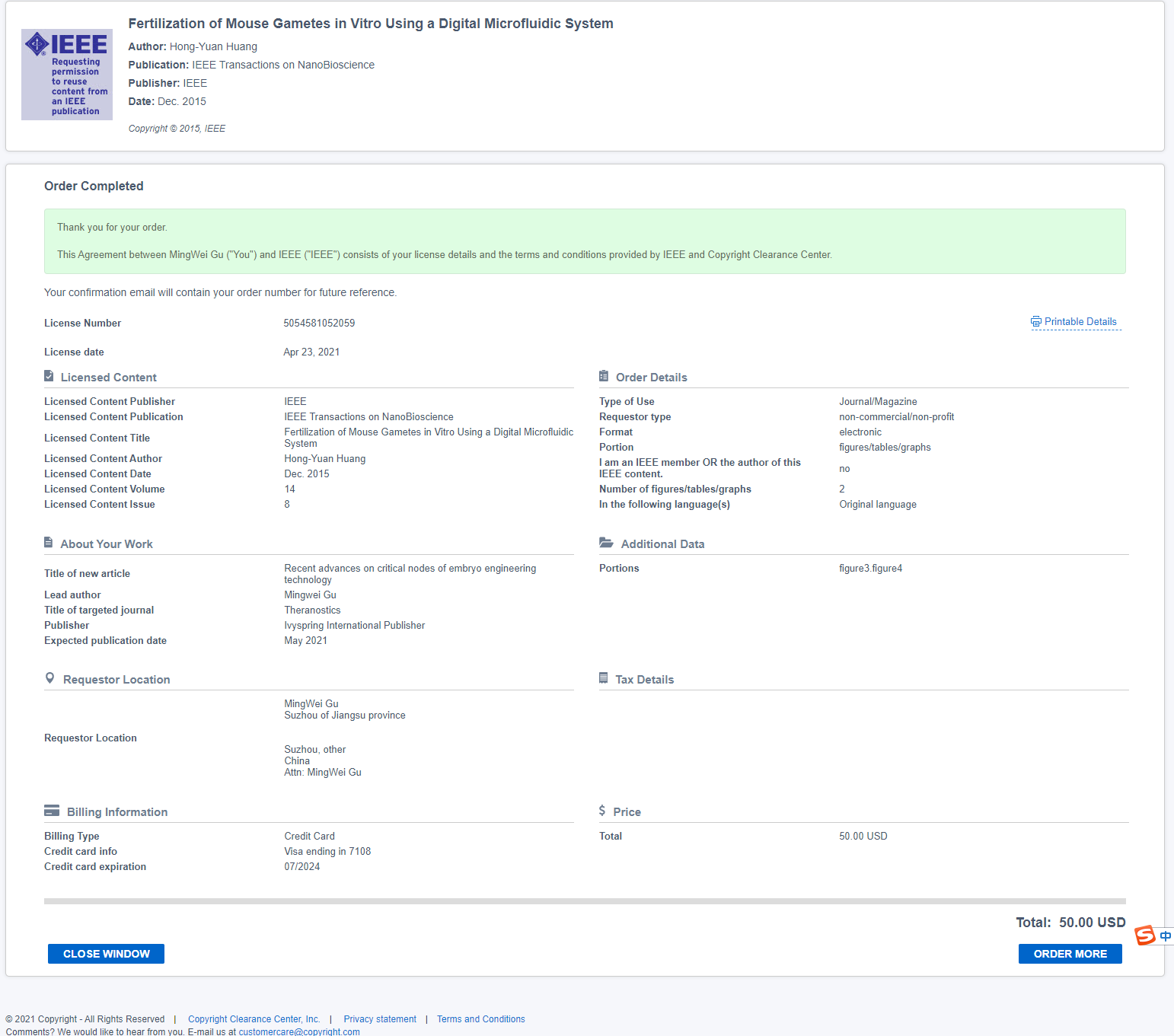

Supplement: Supplementary file 1 — Supplementary figures and tables. [file thnov11p7391s1.zip › Supplementary material/Figure copyright/16d.png]

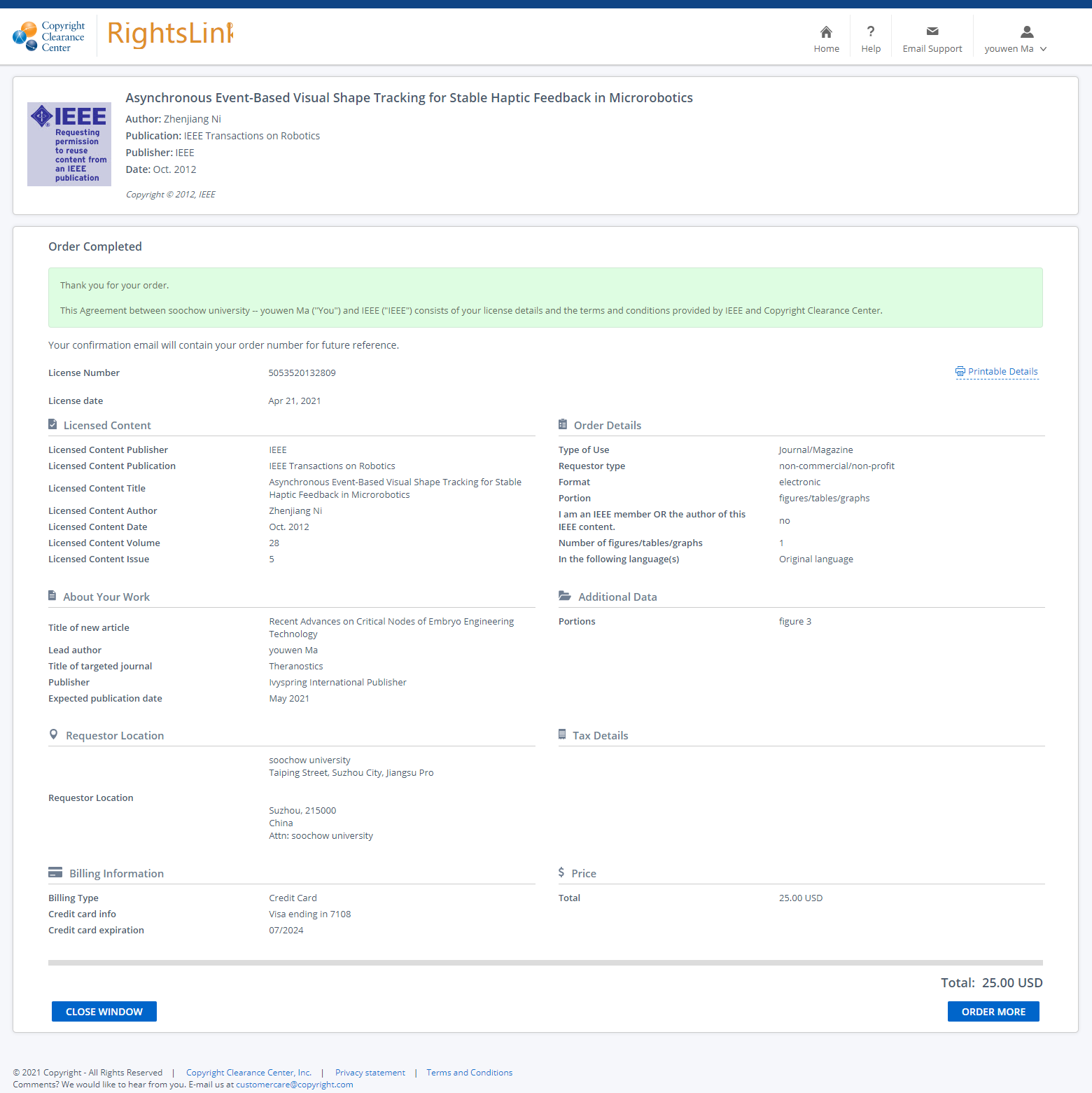

Supplement: Supplementary file 1 — Supplementary figures and tables. [file thnov11p7391s1.zip › Supplementary material/Figure copyright/2A.png]

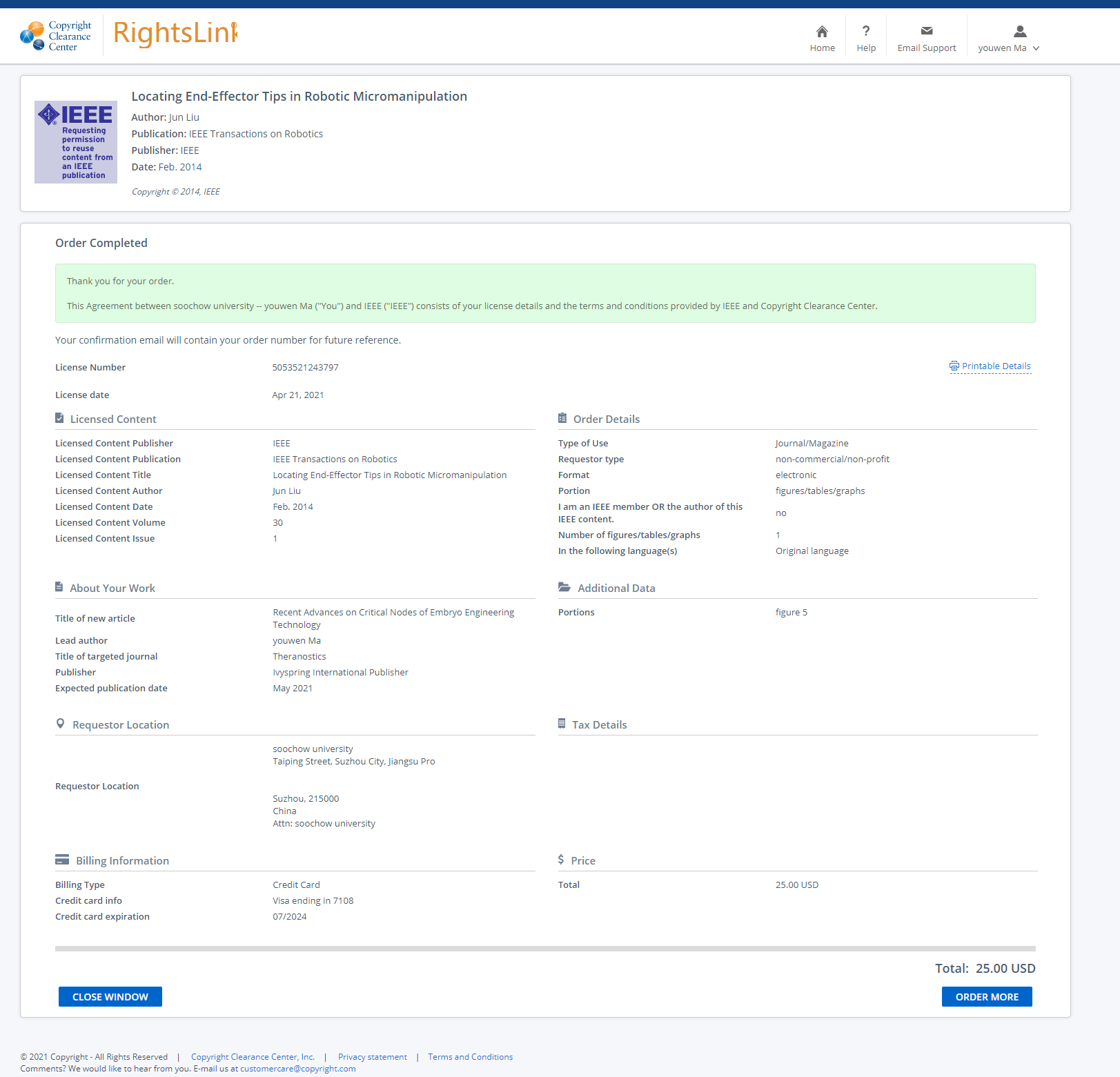

Supplement: Supplementary file 1 — Supplementary figures and tables. [file thnov11p7391s1.zip › Supplementary material/Figure copyright/2B.png]

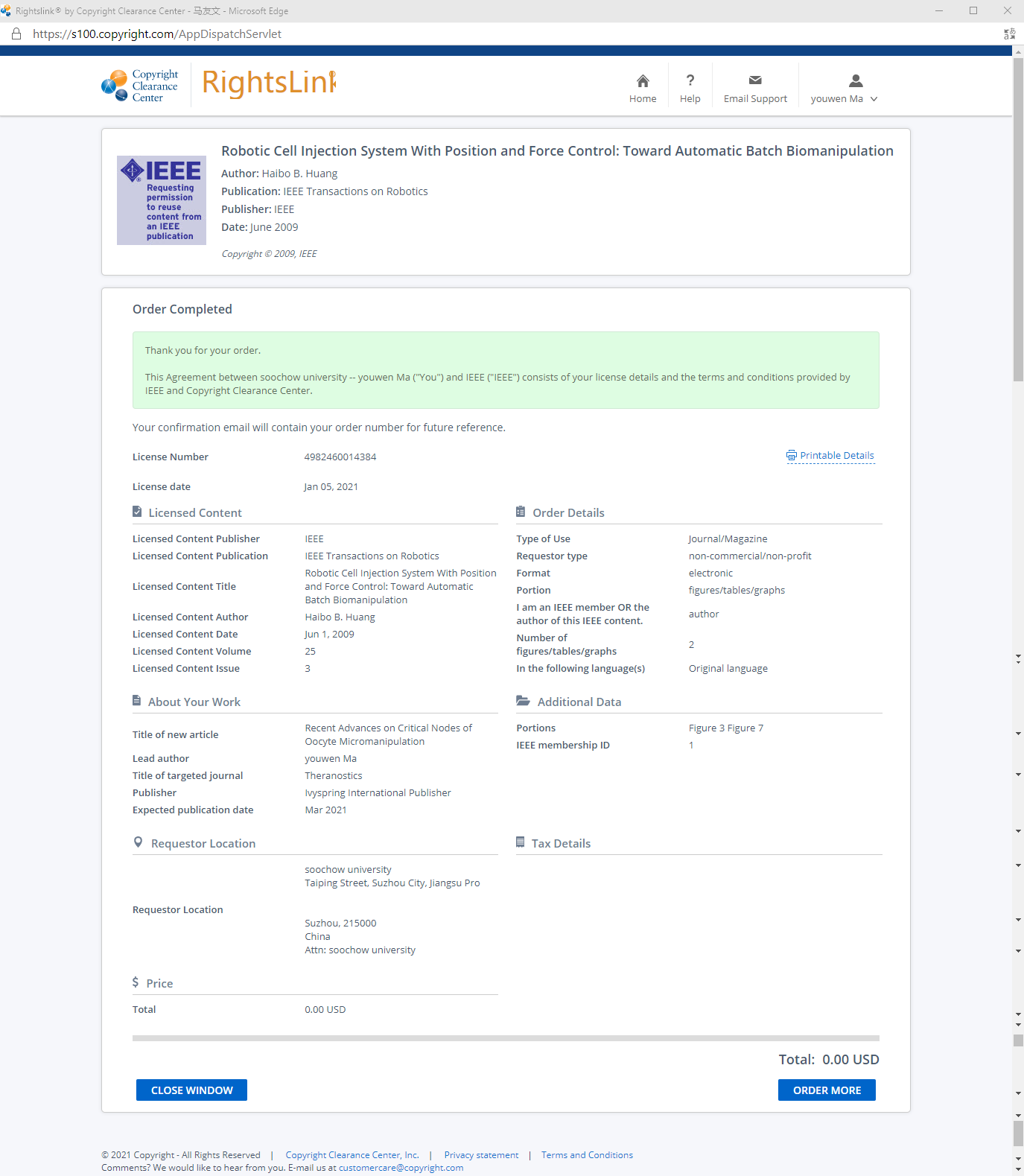

Supplement: Supplementary file 1 — Supplementary figures and tables. [file thnov11p7391s1.zip › Supplementary material/Figure copyright/3A&4A.png]

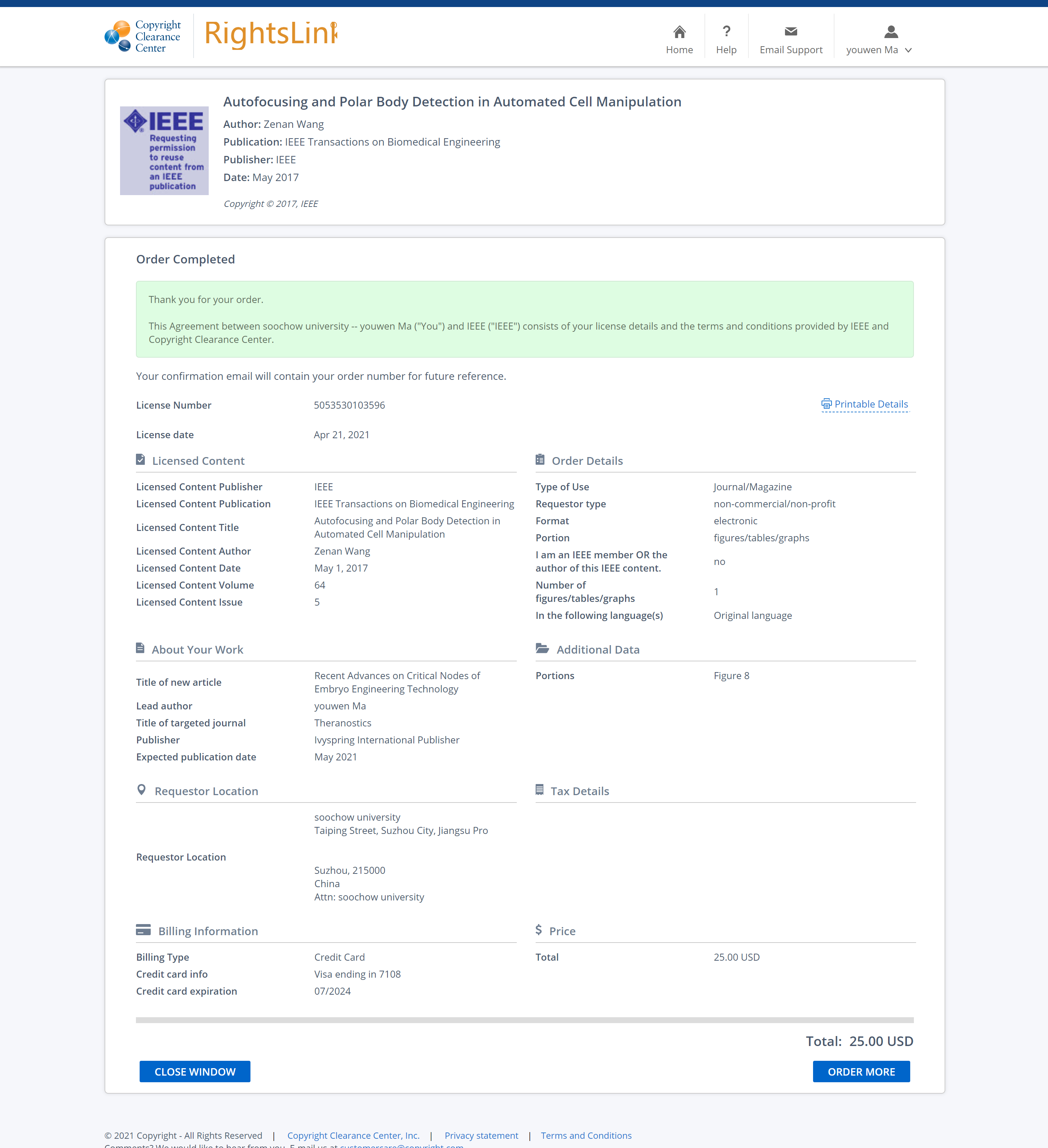

Supplement: Supplementary file 1 — Supplementary figures and tables. [file thnov11p7391s1.zip › Supplementary material/Figure copyright/3B.png]

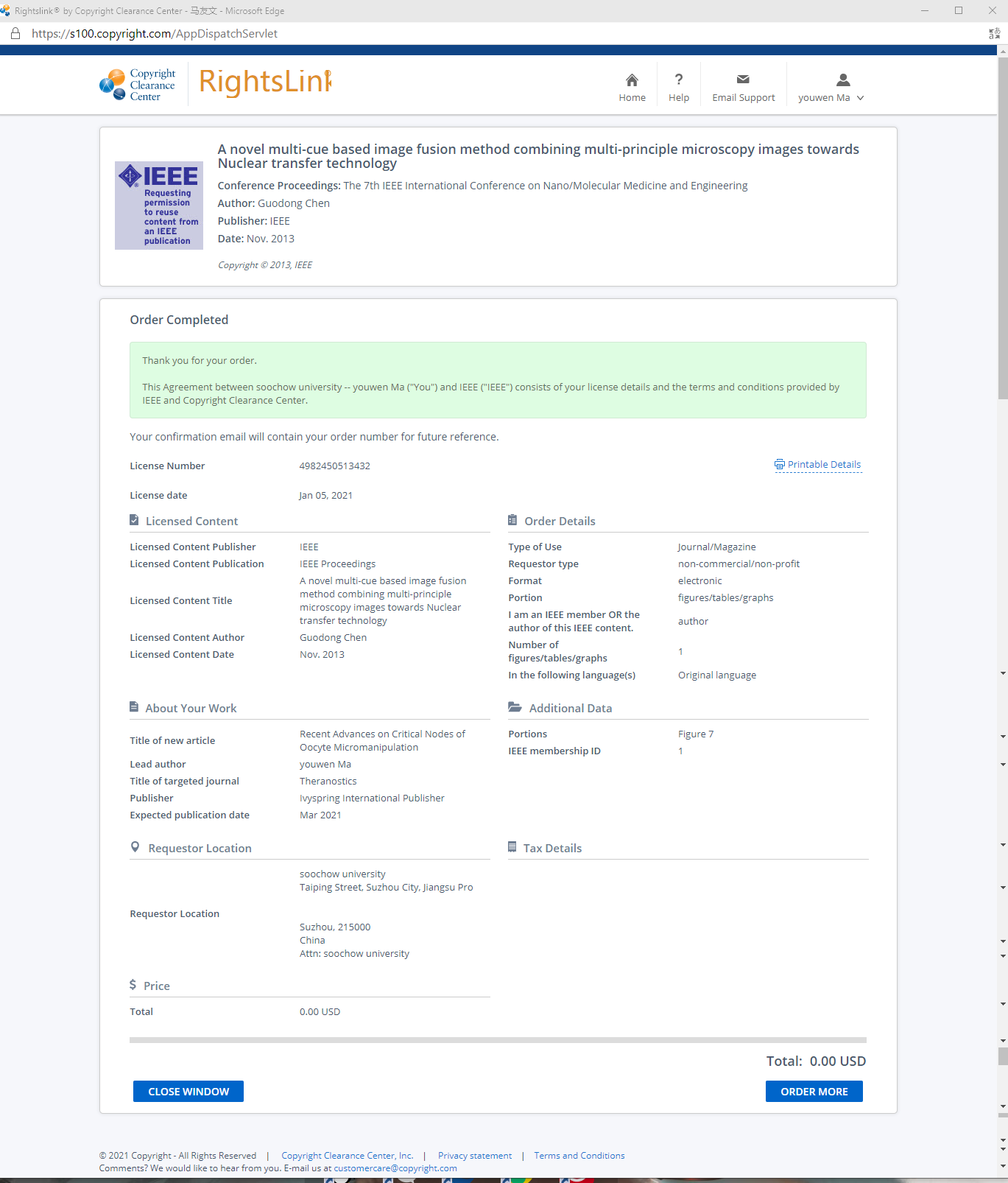

Supplement: Supplementary file 1 — Supplementary figures and tables. [file thnov11p7391s1.zip › Supplementary material/Figure copyright/3C.png]

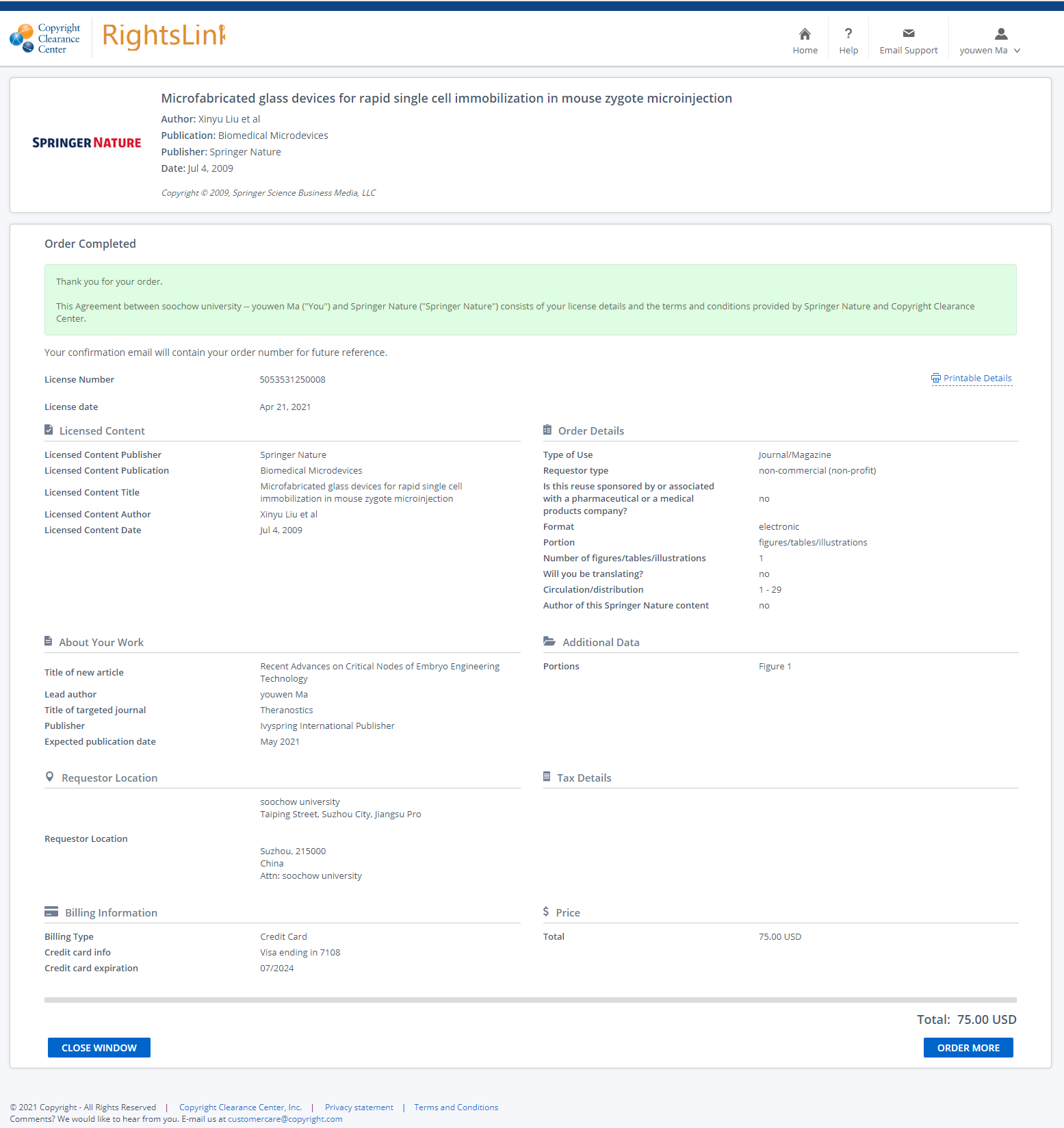

Supplement: Supplementary file 1 — Supplementary figures and tables. [file thnov11p7391s1.zip › Supplementary material/Figure copyright/4B.png]

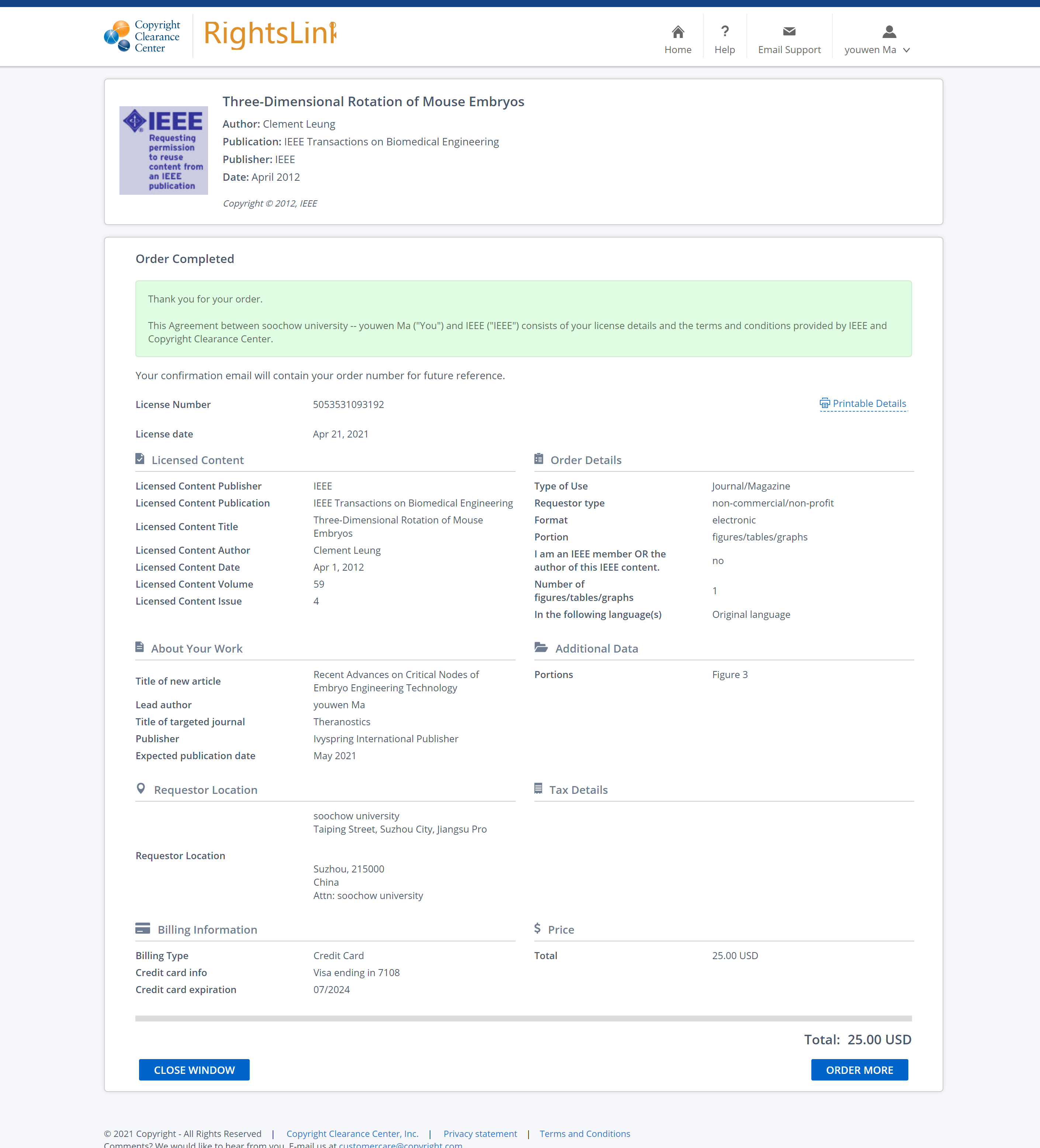

Supplement: Supplementary file 1 — Supplementary figures and tables. [file thnov11p7391s1.zip › Supplementary material/Figure copyright/5A.png]

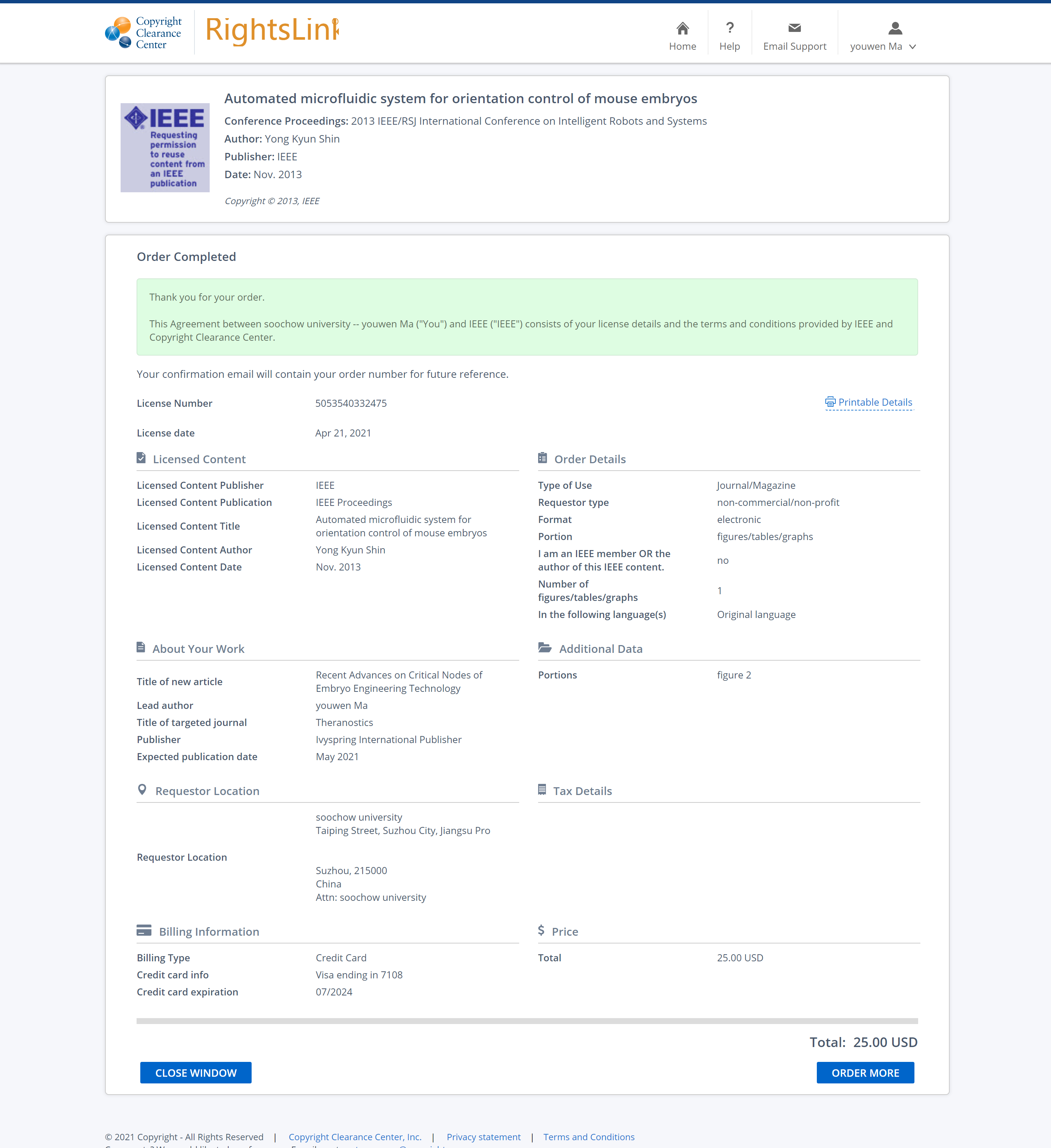

Supplement: Supplementary file 1 — Supplementary figures and tables. [file thnov11p7391s1.zip › Supplementary material/Figure copyright/5B.png]

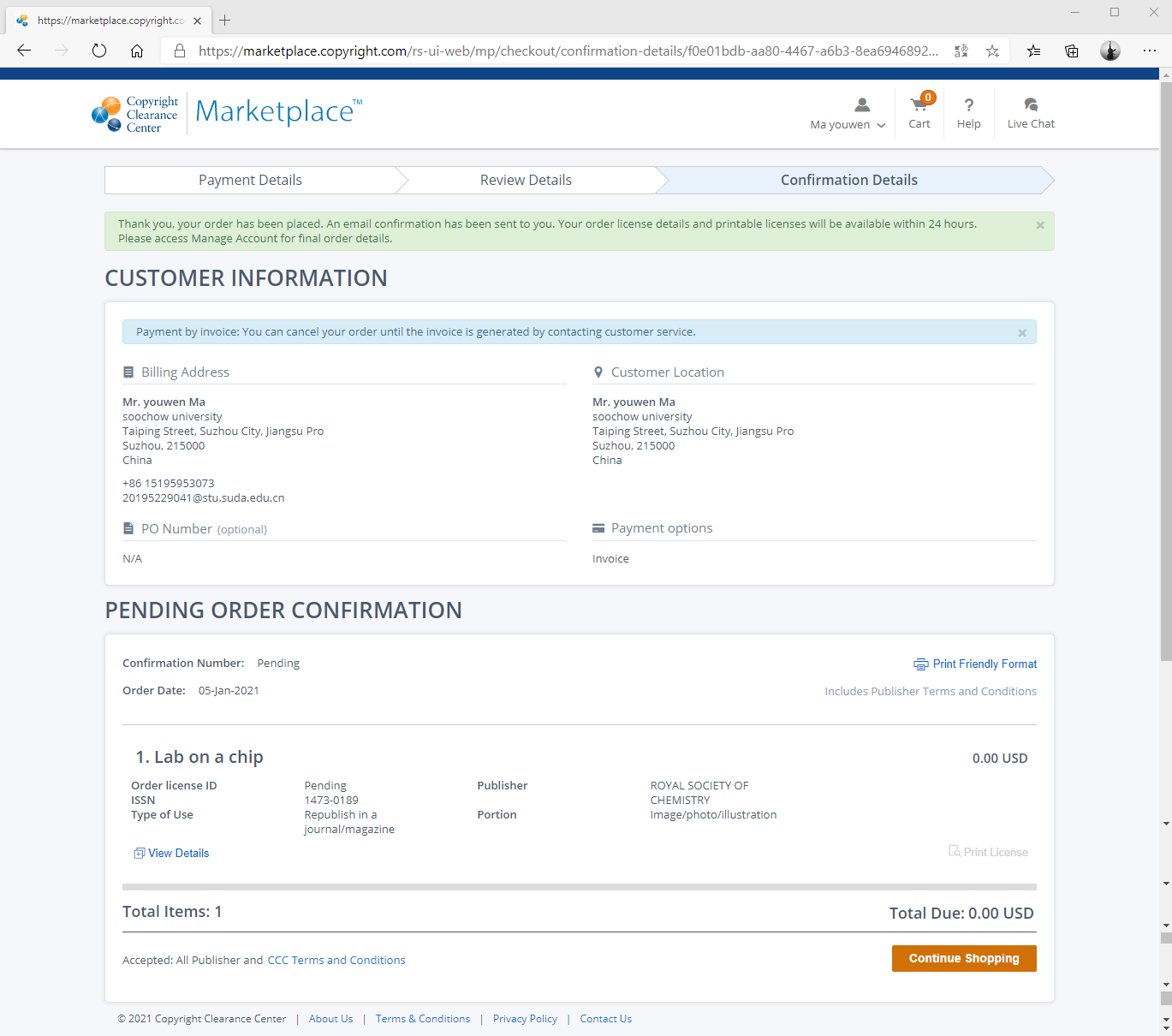

Supplement: Supplementary file 1 — Supplementary figures and tables. [file thnov11p7391s1.zip › Supplementary material/Figure copyright/6A.png]

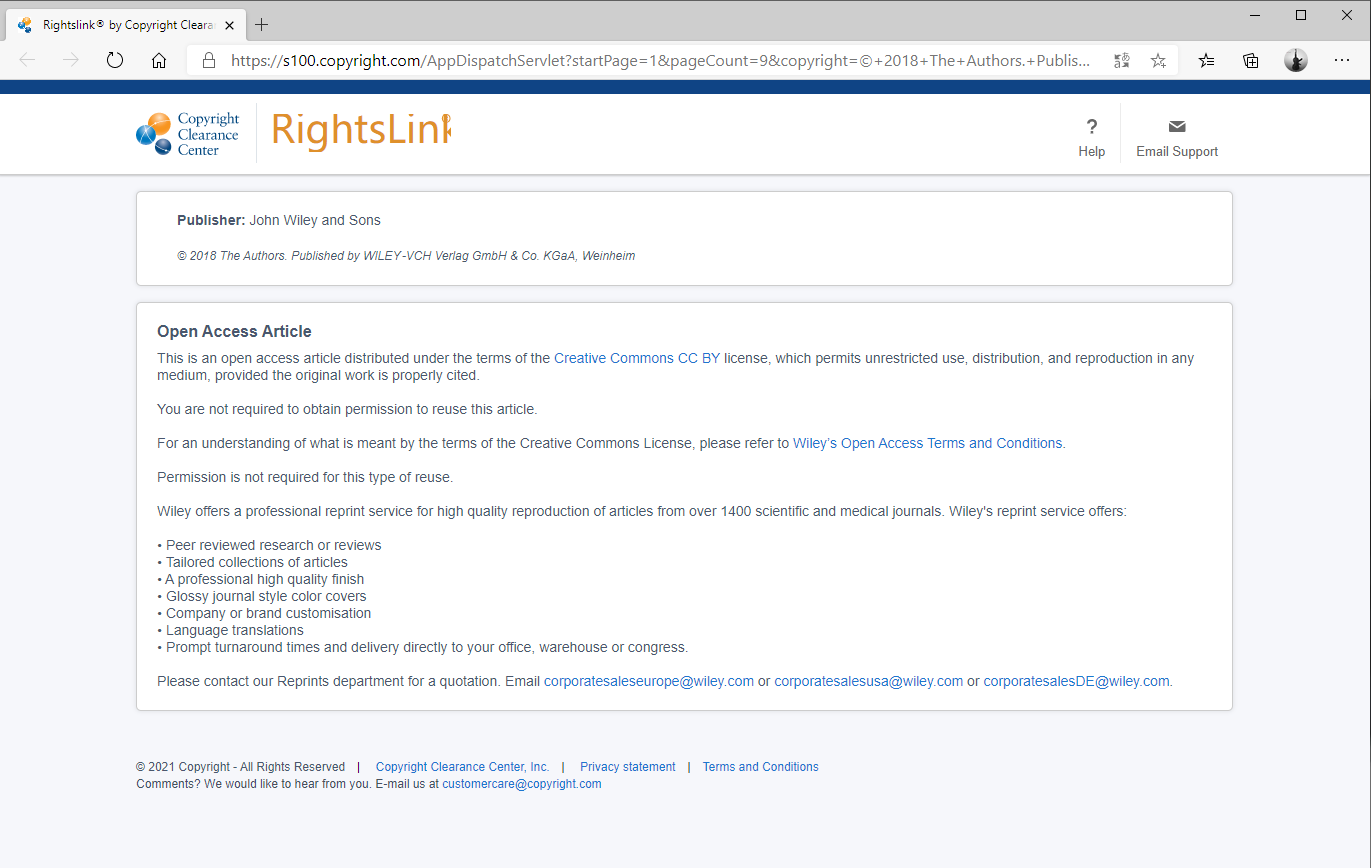

Supplement: Supplementary file 1 — Supplementary figures and tables. [file thnov11p7391s1.zip › Supplementary material/Figure copyright/6B.jpg]

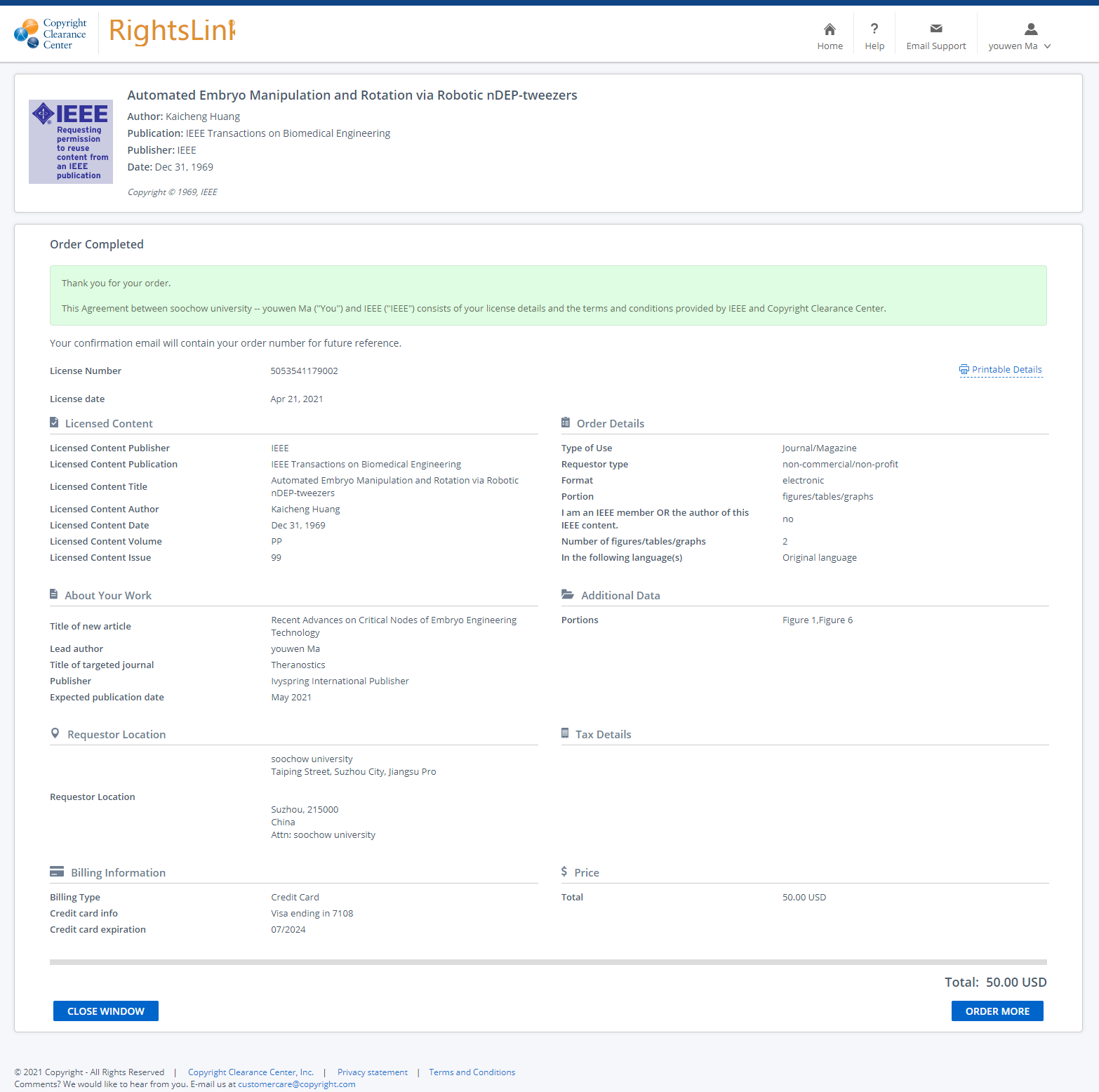

Supplement: Supplementary file 1 — Supplementary figures and tables. [file thnov11p7391s1.zip › Supplementary material/Figure copyright/6C.png]

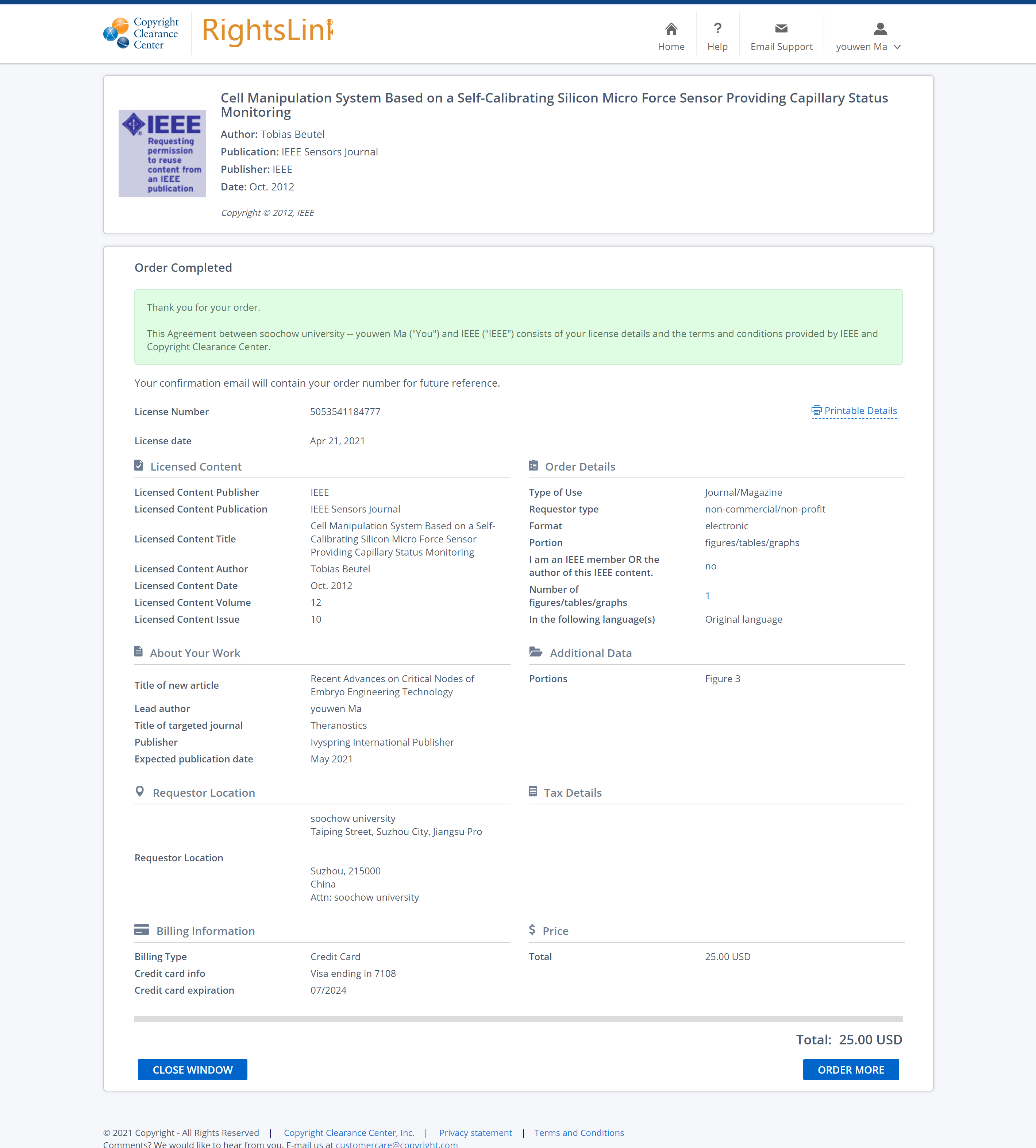

Supplement: Supplementary file 1 — Supplementary figures and tables. [file thnov11p7391s1.zip › Supplementary material/Figure copyright/7A.png]

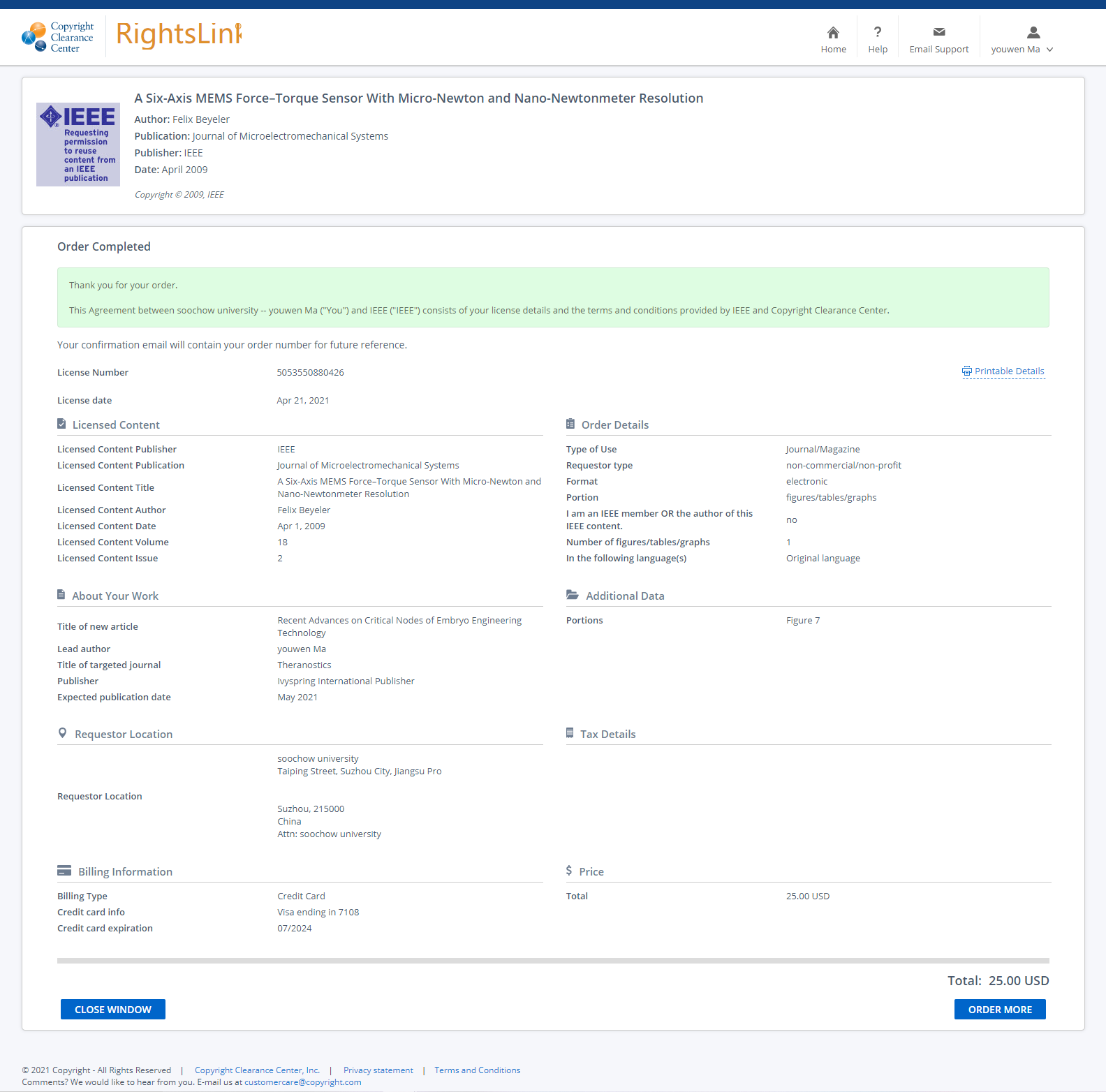

Supplement: Supplementary file 1 — Supplementary figures and tables. [file thnov11p7391s1.zip › Supplementary material/Figure copyright/7B.png]

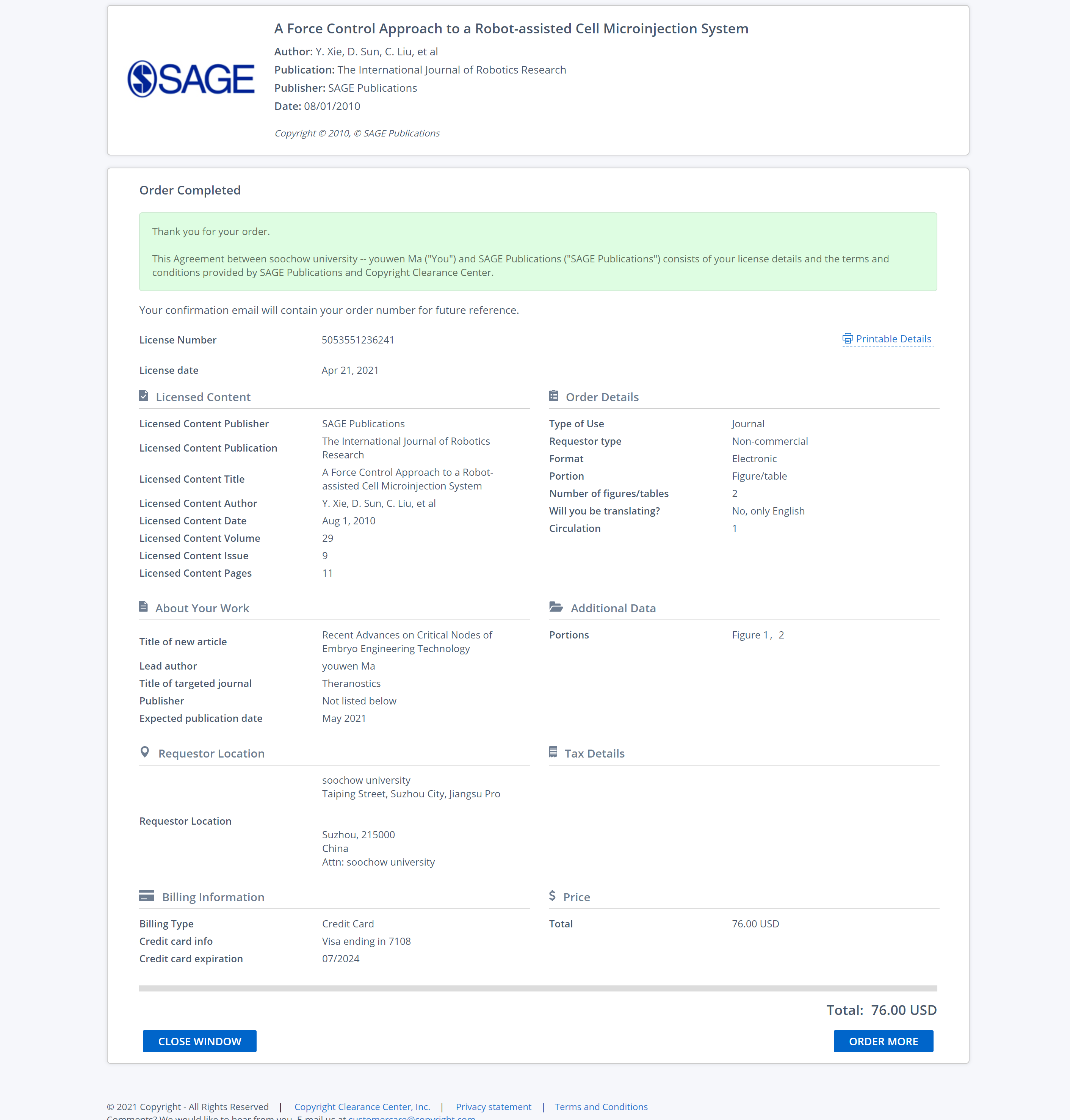

Supplement: Supplementary file 1 — Supplementary figures and tables. [file thnov11p7391s1.zip › Supplementary material/Figure copyright/7C&8A.png]

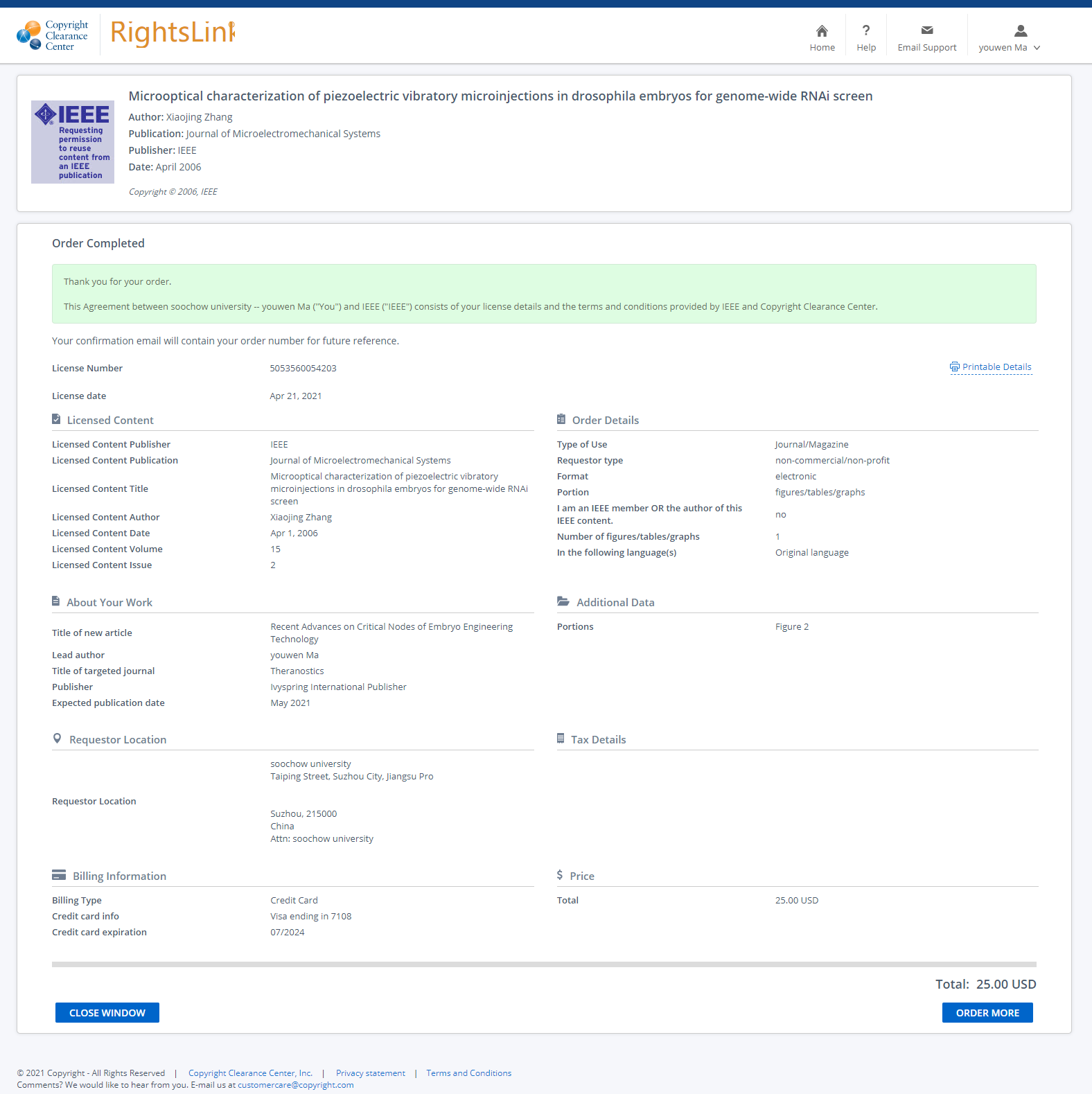

Supplement: Supplementary file 1 — Supplementary figures and tables. [file thnov11p7391s1.zip › Supplementary material/Figure copyright/7D.png]

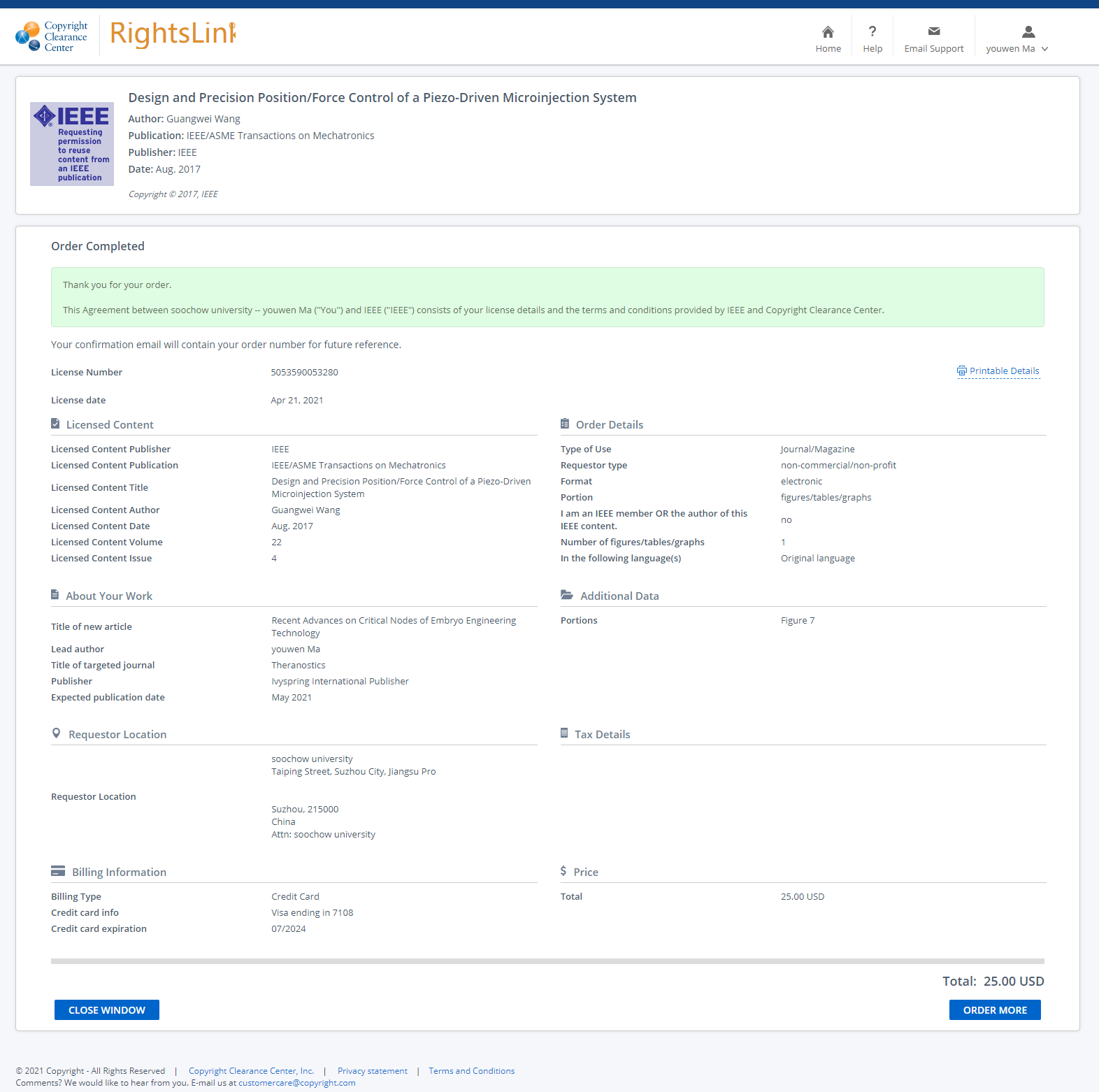

Supplement: Supplementary file 1 — Supplementary figures and tables. [file thnov11p7391s1.zip › Supplementary material/Figure copyright/8B.png]

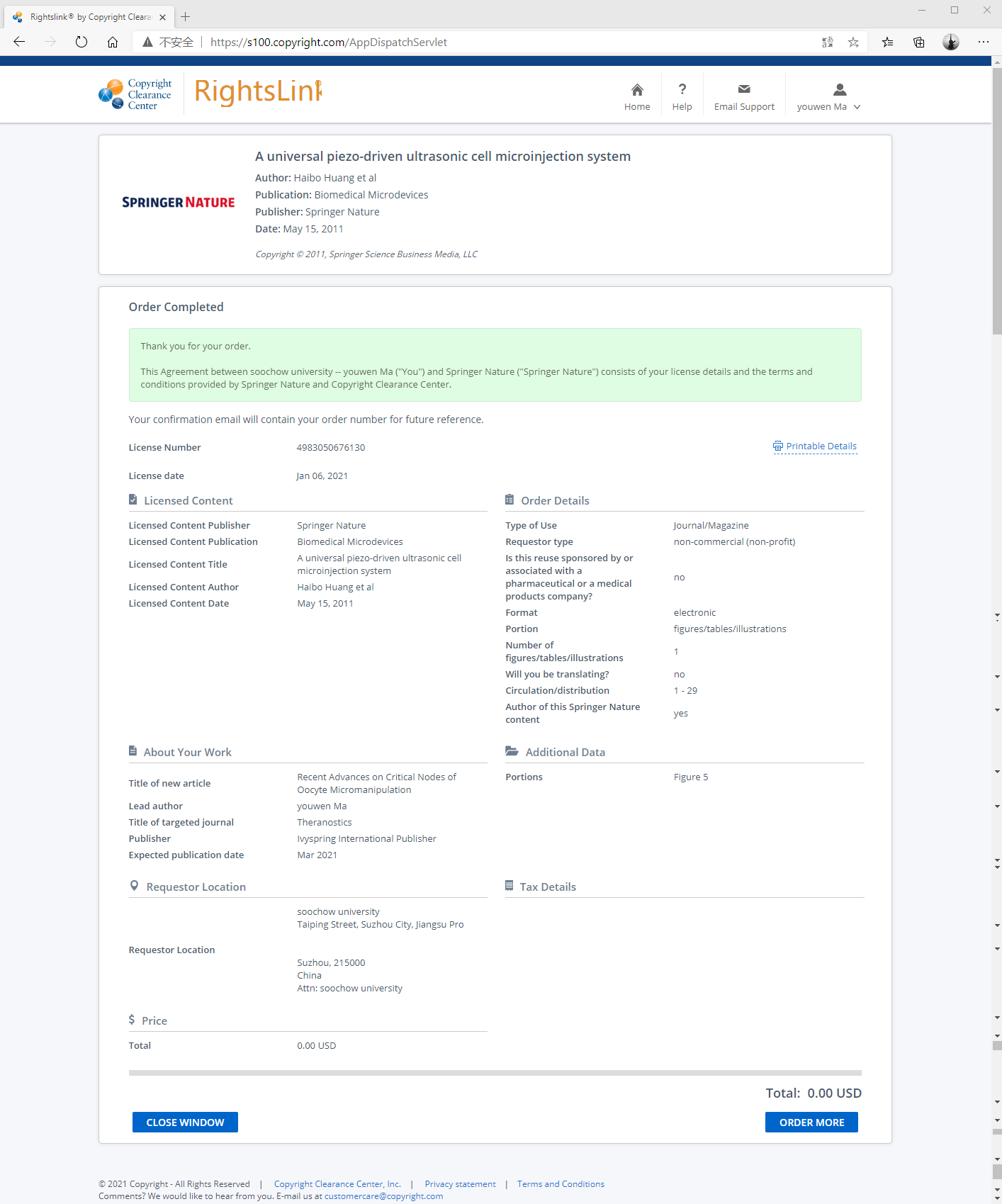

Supplement: Supplementary file 1 — Supplementary figures and tables. [file thnov11p7391s1.zip › Supplementary material/Figure copyright/9A.png]

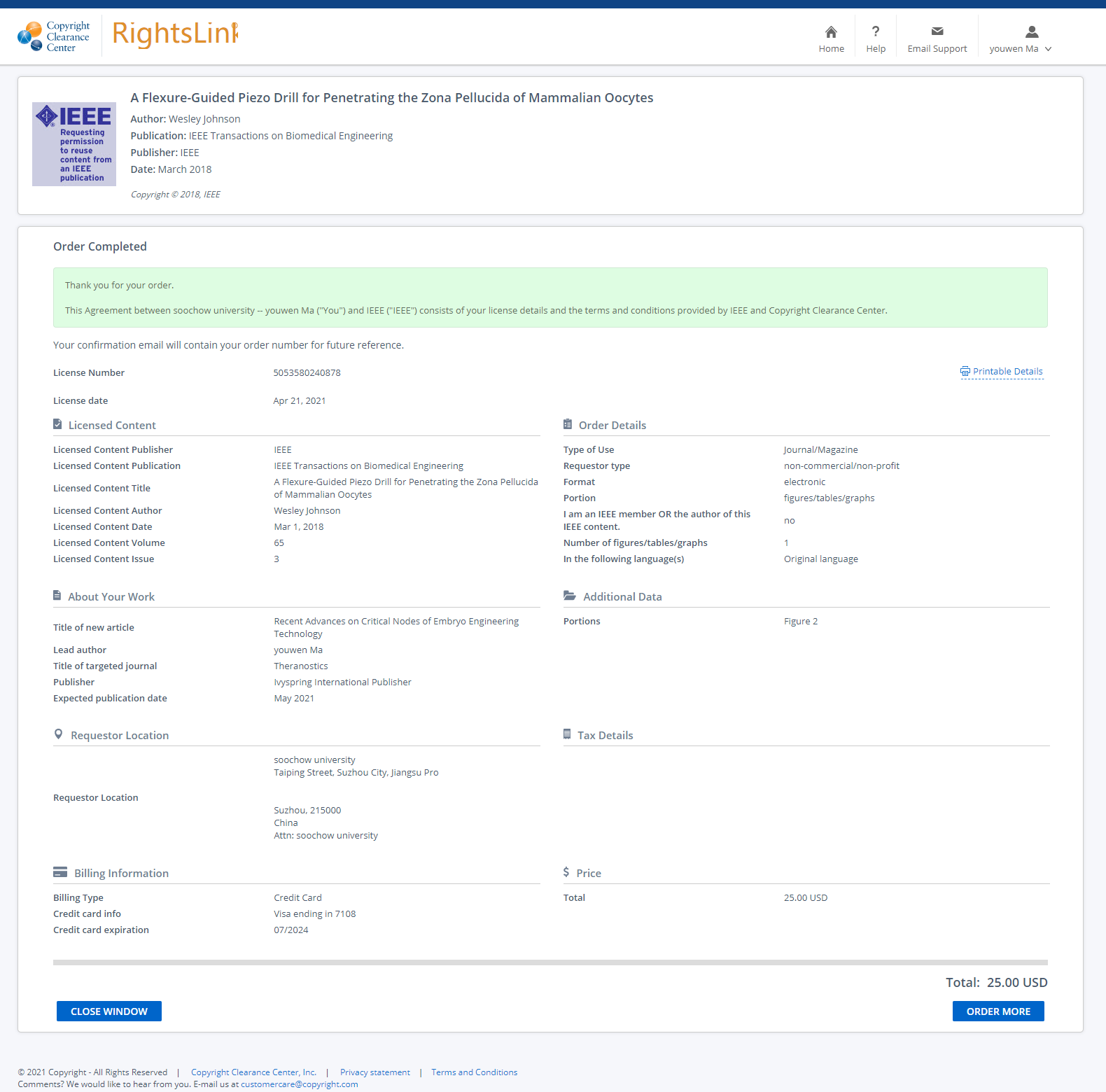

Supplement: Supplementary file 1 — Supplementary figures and tables. [file thnov11p7391s1.zip › Supplementary material/Figure copyright/9B.png]

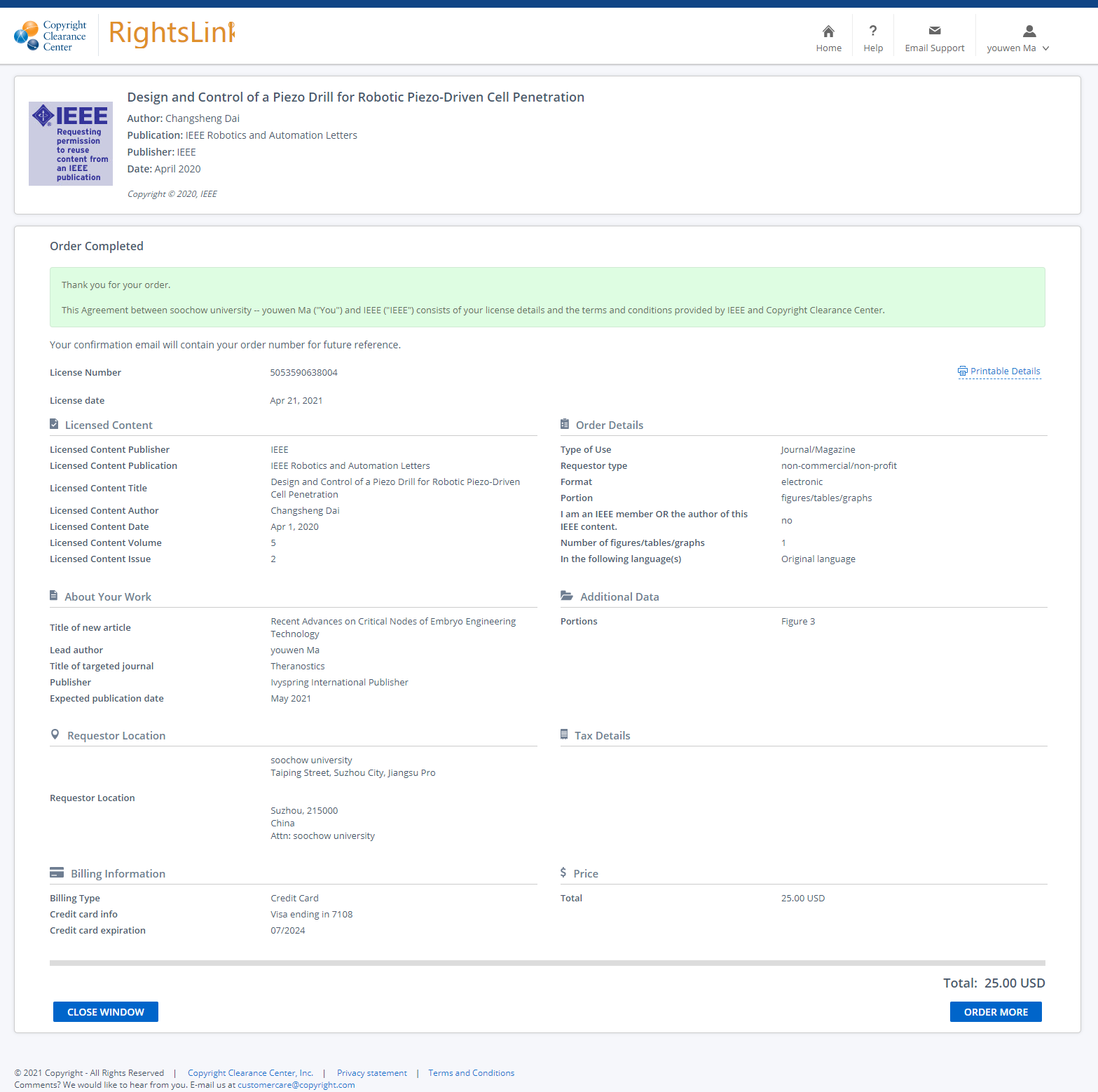

Supplement: Supplementary file 1 — Supplementary figures and tables. [file thnov11p7391s1.zip › Supplementary material/Figure copyright/9C.png]
